# Predispositional genome sequencing in healthy adults: design, participant characteristics, and early outcomes of the PeopleSeq Consortium

**DOI:** 10.1186/s13073-019-0619-9

**Published:** 2019-02-27

**Authors:** Emilie S. Zoltick, Michael D. Linderman, Molly A. McGinniss, Erica Ramos, Madeleine P. Ball, George M. Church, Debra G. B. Leonard, Stacey Pereira, Amy L. McGuire, C. Thomas Caskey, Saskia C. Sanderson, Eric E. Schadt, Daiva E. Nielsen, Scott D. Crawford, Robert C. Green, for the PeopleSeq Consortium

**Affiliations:** 1 Division of Genetics, Department of Medicine, Brigham and Women’s Hospital, 41 Avenue Louis Pasteur, Suite 301, Boston, MA 02115 USA; 2 Section of Preventive Medicine and Epidemiology, Department of Medicine, Boston University School of Medicine, 801 Massachusetts Avenue, Suite 470, Boston, MA 02118 USA; 3 Department of Computer Science, Middlebury College, McCardell Bicentennial Hall, Middlebury, VT 05753 USA; 4 Department of Genetics and Genomic Sciences, Icahn School of Medicine at Mount Sinai, One Gustave L. Levy Place, Box 1498, New York, NY 10029 USA; 5 Illumina, Inc, 5200 Illumina Way, San Diego, CA 92122 USA; 6 Geisinger National Precision Health, Geisinger, 6101 Executive Blvd, Suite 110, North Bethesda, MD 20852 USA; 7 Open Humans Foundation, Boston, MA USA; 8 Harvard Personal Genome Project, Harvard Medical School, Boston, MA USA; 9 Department of Genetics, Harvard Medical School, 77 Avenue Louis Pasteur, Room 238, Boston, MA 02115 USA; 10 Wyss Institute for Biologically Inspired Engineering, Harvard University, 3 Blackfan Circle, Boston, MA 02115 USA; 11 Department of Pathology and Laboratory Medicine, Robert Larner, M.D, College of Medicine of the University of Vermont, 89 Beaumont Avenue, Courtyard at Given S269, Burlington, VT 05405 USA; 12 Center for Medical Ethics and Health Policy, Baylor College of Medicine, One Baylor Plaza, Suite 310D, Houston, TX 77030 USA; 13 Molecular and Human Genetics, Baylor College of Medicine, One Baylor Plaza, Mail Stop BCM225, Houston, TX 77030 USA; 14 Department of Behavioural Science and Health, University College London, Gower Street, London, WC1E 6BT UK; 15 School of Human Nutrition, McGill University, 21111 Lakeshore Road, Ste-Anne-de-Bellevue, Quebec H9X 3V9 Canada; 16 SoundRocket, 950 Victors Way, Suite 50, Ann Arbor, MI 48108 USA; 17 The Broad Institute of MIT and Harvard, 415 Main Street, Cambridge, MA 02142 USA; 18 Harvard Medical School, Boston, MA USA; 19 Partners HealthCare Personalized Medicine, Boston, MA 02115 USA

**Keywords:** Personal genome sequencing, Return of results, Genomics, Test utility, Public health

## Abstract

**Background:**

Increasing numbers of healthy individuals are undergoing predispositional personal genome sequencing. Here we describe the design and early outcomes of the PeopleSeq Consortium, a multi-cohort collaboration of predispositional genome sequencing projects, which is examining the medical, behavioral, and economic outcomes of returning genomic sequencing information to healthy individuals.

**Methods:**

Apparently healthy adults who participated in four of the sequencing projects in the Consortium were included. Web-based surveys were administered before and after genomic results disclosure, or in some cases only after results disclosure. Surveys inquired about sociodemographic characteristics, motivations and concerns, behavioral and medical responses to sequencing results, and perceived utility.

**Results:**

Among 1395 eligible individuals, 658 enrolled in the Consortium when contacted and 543 have completed a survey after receiving their genomic results thus far (mean age 53.0 years, 61.4% male, 91.7% white, 95.5% college graduates). Most participants (98.1%) were motivated to undergo sequencing because of curiosity about their genetic make-up. The most commonly reported concerns prior to pursuing sequencing included how well the results would predict future risk (59.2%) and the complexity of genetic variant interpretation (56.8%), while 47.8% of participants were concerned about the privacy of their genetic information. Half of participants reported discussing their genomic results with a healthcare provider during a median of 8.0 months after receiving the results; 13.5% reported making an additional appointment with a healthcare provider specifically because of their results. Few participants (< 10%) reported making changes to their diet, exercise habits, or insurance coverage because of their results. Many participants (39.5%) reported learning something new to improve their health that they did not know before. Reporting regret or harm from the decision to undergo sequencing was rare (< 3.0%).

**Conclusions:**

Healthy individuals who underwent predispositional sequencing expressed some concern around privacy prior to pursuing sequencing, but were enthusiastic about their experience and not distressed by their results. While reporting value in their health-related results, few participants reported making medical or lifestyle changes.

**Electronic supplementary material:**

The online version of this article (10.1186/s13073-019-0619-9) contains supplementary material, which is available to authorized users.

## Background

Whole genome and exome sequencing have well-established clinical utility for rare disease diagnosis [1, 2] and personalized cancer treatment [3]. Driven by decreasing costs, sequencing is increasingly being used in other clinical, research, and commercial settings, including as a screening tool in apparently healthy individuals, termed predispositional personal genome sequencing (PPGS) [4]. Individuals pursuing PPGS are not using genome sequencing for diagnostic purposes, but nonetheless are interested in obtaining health-related results. For many participants and providers, there is an expectation that PPGS will eventually enable a more personalized and preventive approach to medicine, in which illness is anticipated or prevented through screening for genetic predispositions to disease [5]. However, the actual clinical utility in healthy individuals is unknown, and the risks, benefits, and costs both to the individual and society are unclear [6, 7]. Despite this uncertainty, there are increasing numbers of PPGS projects underway, both research- and industry-based [4].

Unlike screening tests that detect early signs of disease, PPGS screens for a potential predisposition to disease. Of particular concern are individuals who will be falsely identified as being at risk [6, 7] as a result of analytic errors, interpretation errors, and/or gaps in understanding of the penetrance of each variant, especially in the absence of family history of the condition [8]. These factors may present challenges for clinical management, which in turn, could create unnecessary anxiety and medical surveillance, and drive increased healthcare expenditures [9]. Previous and ongoing PPGS studies have reported numerous examples of successful predispositional identification of individuals with disease or increased disease risk [10, 11]. Recent reports show that as many as 20% of participants in predispositional sequencing cohorts may have a variant with monogenic disease risk [12–14]. At this early stage, however, the precise fraction of individuals who might benefit from sequencing due to disease prevention or early diagnosis is uncertain.

Multiple research studies have sought to improve our understanding of the possible clinical and personal utility of PPGS. The Harvard Personal Genome Project (PGP), launched in 2005, was arguably the first PPGS project, though the return of genomic results and the consequences thereof were not the main purpose [15]. More recent studies, conducted mostly as controlled research protocols in clinical settings, have explored the reactions to sequencing and the return of results to healthy individuals [11, 16–23]. For example, within Geisinger, an integrated healthcare system, there are plans for over 250,000 patient-participants to have their exomes sequenced, most without a specific indication, under the MyCode® Community Health Initiative, an expansive clinical research protocol [21]. The All of Us Research Program, part of the Precision Medicine Initiative (PMI), has publicly committed to providing participants access to the data gathered, which might include genomic information, although the details have not yet been finalized [24]. Multiple biotech companies have launched or announced broad physician-ordered predispositional sequencing panels [25–28] and more consumer-oriented research products [29–32]. These developments reflect the multiple forces, such as changing views on obligations to return study results to participants, disintermediation of traditional medical authorities, and large-scale sequencing efforts seeking pharmaceutical targets, which are accelerating the availability of PPGS.

The Personal Genome Sequencing Outcomes (PeopleSeq) Consortium is a collaborative effort among multiple academic and commercial PPGS projects designed to cost-effectively collect coherent survey data on the short- and long-term outcomes of apparently healthy individuals already undergoing sequencing through one of the Consortium projects. Thus far, the Consortium has enrolled individuals across four PPGS projects who have received a broad range of health-related genomic results from personal genome or exome sequencing. Additional sites are in the process of joining the Consortium, representing a range of both research and commercial sequencing providers. This Consortium then provides a mechanism to examine the outcomes of PPGS in the many different contexts in which this technology is now employed. PeopleSeq participants are innovators and early adopters [33], similar to initial users of other rapidly evolving technologies. Studying PPGS early adopters can provide valuable insights because these individuals have actually used these technologies, and thus can provide concrete evidence of both the risks and benefits [34]. Here we describe the design of the PeopleSeq Consortium and present descriptive findings from the four initial projects.

## Methods

### Overview

The PeopleSeq Consortium, formed in 2014, is a collaboration of PPGS projects designed to study participants’ motivations and experiences and the self-reported medical, behavioral, and economic outcomes of PPGS. Here we report on data collected from participants in the first four projects to contribute to the Consortium: the Harvard PGP [15], the Baylor College of Medicine’s Young Presidents’ Organization (YPO) and MD/PhD Genome Projects [17], Mt. Sinai’s HealthSeq project [19], and Illumina’s Understand Your Genome^Ⓡ^ (UYG) program [35]. All of the projects performed genome or exome sequencing on apparently healthy adults and returned a report of the individual genomic results back to participants. The consent, genetic counseling, and results disclosure processes varied by project (see Additional file 1: Table S1 for an overview of each project and Linderman et al. [4] for a more detailed review of these and similar projects). Clinical genome reports from a CLIA (Clinical Laboratory Improvement Amendments)-certified laboratory were only provided by UYG. UYG was also the only project to send a clinical report to a healthcare provider, while PGP publicly shared participants’ genomes online. Though the type of PPGS results varied by project, all of the projects returned monogenic disease findings in numerous genes (more than the genes specified in the American College of Genetics and Genomics recommendations for reporting of secondary findings) [36, 37]. The PeopleSeq Consortium itself did not perform sequencing of participants but administered common web-based surveys to participants in the collaborating projects. Consent for participation to receive surveys from the PeopleSeq Consortium was entirely separate from consent in the original PPGS project.

Depending on the site and timing of the invitation, participants were invited to enroll and complete PeopleSeq surveys either before or after disclosure of their genomic results. Those who enrolled into the PeopleSeq Consortium after disclosure of their PPGS results received one initial survey (the “catch-up” survey), while those who enrolled before disclosure of their PPGS results received two surveys: a survey after signing up for PPGS but before disclosure of their PPGS results (the pre-disclosure survey) and after disclosure (the post-disclosure survey). At the Consortium’s conception, all participants received the catch-up survey; however, since November 2015, the pre- and post-disclosure surveys were administered whenever possible. All participants received annual follow-up surveys. This longitudinal design allows for assessment of both short- and long-term outcomes. The Partners HealthCare Human Research Committee and the Baylor College of Medicine Institutional Review Board approved the entire study. Each site consulted their Institutional Review Board, as applicable, and received additional approval if necessary.

### Survey design and measures

The PeopleSeq Consortium surveys were developed by an interdisciplinary team of geneticists, genetic counselors, ethicists, psychologists, and survey design researchers. The surveys were adapted from the Impact of Personal Genomics (PGen) Study surveys [38], developed using expert consultation and cognitive interview techniques, with additional questions derived from qualitative interviews in the MedSeq Project [18]. The survey questions assessed sociodemographic characteristics, personal and family health information, prior use of genetic testing, motivations and concerns, psychological impacts, risk perceptions, perceived utility and harms, and behavioral and medical responses (see Additional file 1: Table S2 for a summary of the survey measures and Additional file 2 for copies of the study surveys). Where possible, the surveys utilized validated measures of psychological states [39–41] and decision regret [42], as well as published scales for genomics self-efficacy [43], genome sequencing knowledge [44], and other measures [45].

Genome sequencing knowledge was evaluated using an 11-item assessment with 5-level Likert scale responses (strongly disagree to strongly agree) [44]. Responses for negatively worded items were reversed to make “strongly agree” the correct response for all items. Decision regret was measured with a 5-item instrument with a 5-level Likert scale used for agreement with each item (scored 1–5 points) [42]. The mean score across items was calculated and then converted to a total score out of 100 with higher scores indicating greater regret.

Some survey questions were tailored to each project to reflect differences in the participant experience. For example, questions were populated with site-specific names and some questions only included response options corresponding to the genomic results provided by that project. Following continued analysis of preliminary data, the surveys have been revised over time, refining the wording of some of the questions that were not part of validated measures and adding or deleting questions as necessary. While this refinement has improved comprehension and shortened the surveys, these changes have resulted in some missing data for early participants.

### Participant recruitment and data collection

The PeopleSeq Consortium recruited adults aged 18 years or older who independently decided to pursue PPGS through one of the collaborating projects and, when required by the collaborating project, consented to be contacted about related research projects. All eligible individuals who were previously enrolled in PGP, HealthSeq, and the YPO and MD/PhD Genome Projects received an invitation to participate in the Consortium via email with a description of the study and a link directing them to the web-based survey. Potential participants from UYG were initially approached in person, by email, or via announcement at a UYG program event before receiving the PeopleSeq invitation email.

The invitation email to complete the catch-up survey was sent at least 2 months after receipt of PPGS results. For participants enrolled prior to their PPGS results disclosure, an email invitation to complete the pre-disclosure survey was sent 1 to 3 months before disclosure of genomic results, and the post-disclosure survey was sent 2 to 3 months after receipt of their genomic results. The timing of these emails was coordinated between the collaborating site and SoundRocket (Ann Arbor, MI). If a participant did not complete the pre-disclosure survey prior to receiving their PPGS results, an invitation to complete the catch-up survey was sent. If, at the start of the post-disclosure or catch-up survey, a participant indicated that they had not viewed their PPGS results, they were not permitted to complete the survey until they had done so. Participants were invited to complete an annual follow-up survey. To improve the response rate, each survey invitation email was followed by up to four reminder emails for nonresponders. Participants were also entered into a random drawing to win an Apple Watch.

Informed consent was obtained electronically before participants were given access to the initial study survey (either the pre-disclosure or catch-up survey). The surveys were administered on SoundRocket’s secure web-based platform. Each participant was assigned a unique master identifier, which allowed the participant to securely access the survey system. Participants could freely navigate forward and backward through the survey. All items in the survey were optional and could be skipped. All responses were saved, allowing for partially completed surveys and permitting participants to complete the survey in multiple sessions. At the end of the post-disclosure and catch-up surveys, all participants (except for those from the YPO and MD/PhD Genome Projects) were further asked to consent to share their genomic results with the Consortium. For those participants who consented to share their results, the study coordinated with each collaborating site to obtain this information. PGP participants were asked to provide their PGP identifier linked to their already publicly available genomic data.

### Data analyses

The main analyses presented in this report were limited to the 543 participants who completed the catch-up survey or both the pre- and post-disclosure surveys and responded to the referenced question. A comparison of responders and nonresponders to the initial Consortium invitation was conducted in a substudy of 1093 individuals invited to participate from the two projects, PGP (352 invitees) and UYG (741 invitees), for which case-level nonresponder demographic data were available (data limited to age, gender, and race only). Chi-square tests were used to assess demographic differences by response status.

In the main analyses, for participants completing both the pre- and post-disclosure surveys, sociodemographic characteristics, motivations and concerns when deciding to pursue sequencing, and previous genetic testing were reported on the pre-disclosure survey while all other data were from the post-disclosure survey. Descriptive statistics including means with standard deviations and counts with percentages were computed for participant demographics, psychological outcomes, downstream behavioral and medical actions, perceived utility, and attitudes regarding genome sequencing. Differences in sociodemographic characteristics by project were compared by ANOVA for continuous variables and chi-square or Fisher’s exact test for categorical variables. Psychological, behavioral, and medical responses, perceived utility, and attitudes were compared by survey (pre-/post-disclosure surveys and catch-up survey), given the differences in timing of survey administration. To test differences by survey, the Wilcoxon rank sum test was used for continuous and ordinal measures and chi-square or Fisher’s exact tests were used for categorical measures. Minimal statistically significant differences were observed by survey (Additional file 1: Tables S3 and S4), so the results are presented for all surveys combined. In an exploratory analysis, a sign test was used to compare perceived present and future personal or clinical utility of the genomic results. All statistical tests were two-sided with *p* < 0.05 considered statistically significant. The data were analyzed using SAS software (version 9.4; SAS Institute, Cary, NC). The statistical code is available from the authors upon request.

## Results

**Fig. 1:**
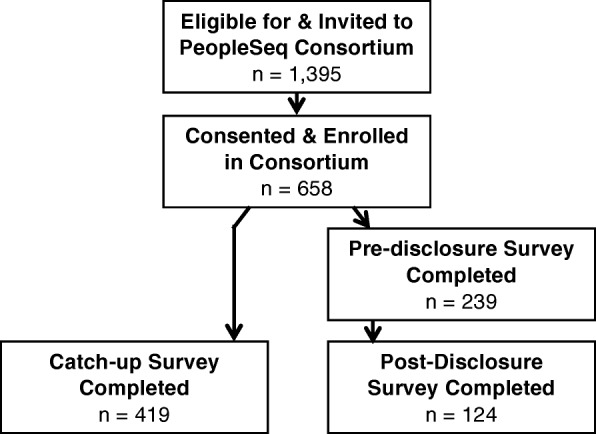
PeopleSeq Consortium enrollment and data collection

A total of 1395 eligible individuals from four projects were invited to participate in the PeopleSeq Consortium between October 2014 and July 2017 (see Fig. 1 for an enrollment flowchart and Additional file 1: Figure S1 for a flowchart by project). A total of 658 individuals consented and enrolled in the study for an initial response rate of 47.2%. A total of 543 participants completed a survey after receiving their genomic results (419 completed the catch-up survey and 124 completed both the pre- and post-disclosure surveys), for a response rate of 38.9%. The post-disclosure survey is pending for 115 participants who either have received but not yet returned the survey or have not yet received their sequencing results.

In a substudy of responders and nonresponders from UYG and PGP in which case-level demographic nonresponder data were available, the initial response rate was 30.7%. Females were overrepresented among the responders compared to nonresponders (39.6% versus 32.2%, respectively, *p* = 0.018; see Additional file 1: Figure S2). There were borderline statistically significant differences in the distributions of race and age by response status in which a higher percentage of white and younger adults were responders, particularly for PGP.

The sociodemographic characteristics of the 543 participants who completed a survey after receiving their genomic results are summarized in Table 1. Nearly two thirds of participants were male and most self-identified as white. Almost all participants had at least a college degree, and most participants had an annual household income of $100,000 or greater. When asked about their occupation, 29.1% reported that they were healthcare professionals (including healthcare providers and clinical researchers). Almost all participants described themselves as having good health or better; only 4.0% self-reported fair or poor health. About half of participants reported receiving some form of personal genetic testing prior to undergoing PPGS for this project. More than 70% of these individuals reported using direct-to-consumer (DTC) genetic testing (genotyping rather than whole genome sequencing), and “curiosity about health and traits predicted by my genetic make-up” and ancestry were the most common reasons for using DTC genetic testing. While there were statistically significant differences in the sociodemographic characteristics across the four projects, participants in each of the projects were predominantly older, well-educated, wealthy, and in good health (Additional file 1: Table S5).

**Table 1:** Characteristics of participants with completed post-disclosure or catch-up surveys in the PeopleSeq Consortium (*n* = 543)

Characteristic	No. (%)^a^
Age, mean (± SD; range), years	53.0 (12.8; 22–91)
Gender	
Female	202 (38.0)
Male	326 (61.4)
Other	3 (0.6)
Race	
African American or Black	3 (0.6)
Asian	15 (2.8)
White	485 (91.7)
More than one race or other race	26 (4.9)
Hispanic or Latino	16 (3.0)
Education	
Less than college degree	24 (4.5)
College degree	67 (12.7)
Some graduate school	160 (30.3)
Doctoral or professional degree	278 (52.6)
Annual income	
< $40,000	36 (7.0)
$40,000–$99,999	82 (15.9)
≥ $100,000	397 (77.1)
Marital status	
Married	383 (72.0)
Widowed, divorced, or separated	71 (13.4)
Never married	78 (14.7)
Biological children	370 (69.6)
US resident	469 (88.3)
Self-reported health	
Excellent	167 (35.2)
Very good	210 (44.3)
Good	78 (16.5)
Fair	18 (3.8)
Poor	1 (0.2)
Prior genetic testing	247 (49.8)
Project	
Illumina’s Understand Your Genome	329 (60.6)
The Harvard Personal Genome Project	167 (30.8)
Baylor Young Presidents’ Organization and MD/PhD Genome Projects	28 (5.2)
Mount Sinai’s HealthSeq project	19 (3.5)

*SD* standard deviation

^a^Percentages may not sum to 100 due to rounding. Percentages and means are not all based on total of 543 participants because of missing responses to some survey items. The percent of missing responses ranges between 0 and 12.7% (median = 2.4% missing)

**Fig. 2:**
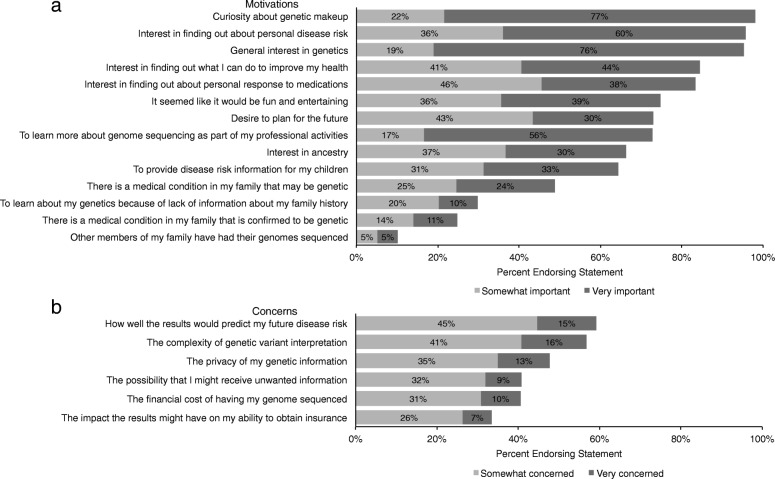
Motivations and concerns when deciding to pursue personal genome sequencing. **a** Motivations for pursuing personal genome sequencing. The light gray indicates the percentage of participants who endorsed the motivation as being somewhat important, and the dark gray indicates the percentage of participants who endorsed the motivation as being very important. **b** Concerns participants had when deciding to pursue sequencing. The light gray indicates the percentage of participants who reported being somewhat concerned about the issue, and the dark gray indicates the percentage of participants who reported being very concerned about the issue. Motivations and concerns were reported on the pre-disclosure and catch-up surveys. Percentages are not all based on denominator of 543 because of missing responses to some survey items. The percent of missing responses ranges between 3.3–23.9% (median = 4.4% missing)

Figure 2 presents the proportion of participants endorsing each motivation for pursuing PPGS from a provided list. When asked what was the most important factor in deciding to pursue PPGS, “curiosity about my genetic make-up” and “interest in finding out about my personal disease risk” ranked highest with about 15% of participants endorsing each (data not shown). When provided with a list of possible concerns when deciding to pursue PPGS, “how well the results would predict my future risk” (59.2%) and “the complexity of genetic variant interpretation” (56.8%) were most frequently selected as factors that participants were somewhat or very concerned about (Fig. 2). Privacy of the genetic information was a concern for 47.8% of participants, but only 12.8% (range 2.5–22.2% across projects) of participants were very concerned about privacy; this was lowest among participants sequenced through PGP and ranged from 16.8% for UYG to 22.2% for the HealthSeq project participants. Only 7.1% (range 3.1–11.1% across projects) of participants were very concerned, while 26.4% (range 18.9–44.4% across projects) were somewhat concerned, about the impact of the PPGS results on their ability to obtain insurance. Strong concerns regarding insurance discrimination were lowest among participants in PGP and highest among participants in the HealthSeq project.

The initial survey after PPGS results disclosure was completed a median of 8.0 months after disclosure (15.2 months after disclosure for participants completing the catch-up survey and 3.8 months for those completing the post-disclosure survey). During this time, most participants (86.8%) reported discussing their results with someone; 81.1% of participants reported sharing their results with a family member (Table 2). Half (51.2%) of participants reported discussing their PPGS results with a healthcare provider, although fewer reported that they had made (13.5%; range 10.6–16.7% across projects) or planned to make (5.8%; range 0–6.9% across projects) an additional appointment with a healthcare provider specifically because of their genomic results. Reporting discussion of the PPGS results with a provider was lowest among PGP participants (41.2%) and highest among participants in the YPO and MD/PhD Genome Projects (79.2%). Of those participants discussing their results with a provider, a primary care physician was the most frequently cited type of provider consulted (81.1%), followed by a genetics specialist (27.9%). The majority of these individuals (81.8%) were somewhat or very satisfied with the discussion of their PPGS results with their healthcare provider, while 6.2% reported that their healthcare provider was unwilling to discuss the meaning of their PPGS results. Dissatisfaction with the discussion of their results was highest for the PGP participants (12.9% not at all satisfied) and lowest for participants from the HealthSeq project and YPO and MD/PhD Genome Projects (0% not at all satisfied), with 6.5% of UYG participants not at all satisfied with the discussion. Due to their PPGS results, 12.5% (range 0–17.4% across projects) of participants reported that they had one or more tests, medical exams, or procedures, and it was only in the HealthSeq project that no one reported having any tests, medical exams, or procedures because of their PPGS results. Of those individuals reporting any tests, exams, or procedures, 13.3% reported undergoing one or more genetic tests to confirm their PPGS findings.

**Table 2:** Reported responses following disclosure of genome sequencing results

	No. (%)^a^
Psychological response	
Decision regret score, mean (± SD; range)^b^	6.6 (13.4; 0–100)
Behavioral and medical responses because of sequencing results	
Communication of test results	
Family	399 (81.1)
Healthcare provider	252 (51.2)
Made appointment with healthcare provider	65 (13.5)
Sought out more information about health or medical topics related to results	237 (48.5)
Made changes to diet	45 (9.4)
Made changes to exercise routine	41 (8.6)
Made changes to medications	29 (7.2)
Made changes to insurance coverage	2 (0.4)

*SD* standard deviation

^a^Percentages and means are not all based on total of 543 participants because of missing responses to some survey items. The percent of missing responses ranges between 8.7 and 25.8% (median = 11.0% missing)

^b^5-item decision regret scale provides a score from 0 to 100

Few participants (12.4%) reported making any lifestyle changes because of their PPGS results: 9.0% and 8.6% reported eating a healthier diet and exercising more, respectively, while less than 1.0% and 0% reported eating a less healthy diet and exercising less. Less than 1.0% of participants reported making changes to any of their insurance coverage including health, life, long-term care, and disability insurance.

Decision regret following PPGS results disclosure was rare with 60.3% of participants reporting no decision regret (score of 0/100) and 95.0% of participants having a score of 25/100 or less. Fewer than 3.0% of participants directly reported regretting their decision to pursue PPGS or experiencing harm due to this decision. When asked how valuable they felt the PPGS experience was, 88.5% reported that the experience was somewhat (44.0%) or very (44.5%) valuable. In ranking the perceived utility of their PPGS results on a scale from 1 to 10, participants believed that the information would be more useful in the future (median = 8.0) than now (median = 6.0), a statistically significant difference (*P* < 0.0001). More than one third of participants (39.5%) somewhat or strongly agreed that they believed they learned something to improve their health that they did not know before, and 58.4% somewhat or strongly agreed that having PPGS made them feel like they had more control over their health (Table 3). However, more than half of participants (54.6%) were disappointed that their PPGS results did not tell them more information. This was not for lack of understanding, as 79.3% of participants agreed that they felt confident that they understood their PPGS results, which corresponded with an 11-item genome sequencing knowledge assessment in which > 70% of participants answered 8 of the 11 items correctly. Almost all participants (96.2%) were somewhat (28.8%) or very (67.4%) satisfied with their decision to obtain PPGS.

**Table 3:** Degree of agreement/disagreement on perceived utility and general attitudes regarding genome sequencing

	No. (%)^a^				
	Strongly disagree	Somewhat disagree	Neither agree nor disagree	Somewhat agree	Strongly agree
Perceived utility of genome sequencing					
I learned something to improve my health that I did not know before	70 (14.6)	86 (18.0)	133 (27.8)	105 (22.0)	84 (17.6)
Having personal genome sequencing made me feel like I have more control over my health	42 (8.7)	44 (9.1)	115 (23.8)	181 (37.5)	101 (20.9)
What I learned from my personal genome sequencing will help reduce my chances of getting sick	83 (17.2)	104 (21.6)	175 (36.3)	78 (16.2)	42 (8.7)
The information that I received about my genome will influence how I manage my health in the future	64 (13.3)	50 (10.4)	137 (28.4)	171 (35.5)	60 (12.5)
I am disappointed that my results did not tell me more information	68 (14.2)	63 (13.1)	87 (18.1)	156 (32.5)	106 (22.1)
Attitudes regarding genome sequencing					
Personal genomic information should be part of a standard medical record	7 (1.5)	34 (7.2)	54 (11.4)	152 (32.2)	225 (47.7)
Health insurance should cover personal genome sequencing	26 (5.5)	56 (11.8)	95 (20.0)	128 (26.9)	171 (35.9)
Personal genome sequencing should only be available to people through their doctor	177 (37.3)	109 (23.0)	62 (13.1)	65 (13.7)	61 (12.9)

^a^Percentages may not sum to 100 due to rounding. Percentages are not all based on denominator of 543 because of missing responses to some survey items. The percent of missing responses ranges between 11.0 and 13.1% (median = 11.8% missing)

Following PPGS, 84.2% of participants felt somewhat (25.5%) or very (58.7%) comfortable with the idea of sharing their entire genome sequence in general. As long as their identity remained anonymous, over 65% of participants across the projects would be willing to share their entire genome publicly. Most participants were also in agreement that personal genomic information should be part of the standard medical record (79.9%) and that health insurance should cover PPGS (62.8%) (Table 3). A minority of participants agreed that PPGS should only be available to people through their doctor (26.6%). Almost a quarter of participants (23.4%) both strongly agreed that personal genomic information should be part of the medical record and strongly disagreed that sequencing should only be available to people through their doctor.

## Discussion

While an increasing number of healthy individuals are receiving PPGS results, the balance of benefits, harms, and downstream costs are still unclear. The PeopleSeq Consortium was formed to systematically collect short- and long-term medical, behavioral, and economic outcomes of PPGS. In this report, we have described the design and implementation of the PeopleSeq Consortium surveys, and the early data from four academic and industry PPGS projects.

Consistent with other PPGS studies, such as the NIH ClinSeq study [46], and with descriptions of users of DTC genetic testing [38, 47], most PeopleSeq participants are white and well-educated with high annual household incomes. Participants were motivated to undergo PPGS because of a general curiosity about their genetic make-up and disease risk, despite considering themselves to be healthy. Many of these participants had previously used DTC genetic testing for similar reasons. These early adopters were also highly knowledgeable about basic genomics concepts. Unsurprisingly, among individuals who all chose to undergo PPGS, concerns regarding privacy and insurance discrimination, which are often cited as serious deterrents [48, 49], were modest. Additionally, strong privacy and insurance discrimination concerns were lowest for PGP participants whose genomes were shared publicly, and these concerns were not relatively high for UYG participants whose clinical genome reports were sent directly to their physicians.

The PeopleSeq Consortium is a self-selected group of early adopters of genome sequencing for whom the perceived risks of PPGS were not necessarily a deterrent. Similarly, in the ClinSeq study, which enrolled middle-aged adults from the general population who consented to undergo genome sequencing in a clinical research setting, participants were found to be high in dispositional optimism and resilience, personality traits that could make these individuals more accepting of this new technology and their genomic information despite the psychological risks [46]. The risk-taking behavior and demographics of the PeopleSeq Consortium is characteristic of innovators and early adopters of a new technology, who are the first to try an innovation and often provide the evidence needed before adoption by the majority, as described by the Diffusion of Innovations Theory [33]. These characteristics may be most apparent among participants from PGP who were the least concerned about privacy and insurance discrimination despite their genomic information being made publicly available online as part of the PGP’s unique approach to genomic research. [15] While individuals enrolled in PeopleSeq across all of the projects are clearly non-representative, the general population also appears highly receptive to learning about their genomic information. As part of focus groups conducted among a sample of Geisinger patient-participants in establishing the MyCode® Community Health Initiative, it was reported that a majority of participants were in favor of the return of genomic results despite possible anxiety or lack of clinical actionability [21]. In a population-based sample of U.S. adults surveyed to inform the design of the PMI’s All of Us Research Program, there was a high level of willingness to participate in the proposed study, and most respondents were interested in receiving their personal health information, including genetic information [50]. Despite the apparent enthusiasm from the general public, these responses may not translate into actual participation [51]. Thus, while the PeopleSeq Consortium may not be representative of the larger populations that may undergo PPGS in the future, information reported by these innovators and early adopters may provide insights that will inform future policies and potentially promote wider adoption of the technology. The outcomes reported by the current users of PPGS contribute to the evidence needed for (or against) the utility of sequencing and provide information on the medical resources that may be necessary as the use of sequencing expands both commercially and clinically.

The homogenous sociodemographic characteristics of these early adopters of genome sequencing, who are predominantly white, wealthy, and well-educated, are similar to the demographics of innovators and early adopters of many new technologies [33], but should still be considered. This Consortium did not provide sequencing but enrolled participants who independently sought genome sequencing through established programs, thus reflecting the lack of diversity that has long been acknowledged in the research and genomics community [52–54]. Limited representation can restrict the possible benefits of genomics in underrepresented ethnic and racial populations due to greater variant misclassification in these populations, perpetuating health disparities [52, 55, 56]. These findings reinforce the need for improved efforts for inclusivity in research and access to genome sequencing, which is an aim of the PMI’s All of Us Research Program [57]. The early adopters in the PeopleSeq Consortium, who are pursuing sequencing in its current state, may provide the much needed data to address the concerns about PPGS, such as the costs of follow-up care, that may limit participation by underrepresented groups [54].

After a median of 8 months from disclosure of their PPGS results, only 13.5% of PeopleSeq participants reported making an additional appointment with a healthcare provider specifically because of their results, and this was similar across projects despite differences in the PPGS reports provided. Slightly more than 50% of participants, however, reported that they discussed their results with their healthcare provider, suggesting that most of these individuals were integrating this information into their regular care rather than making specific appointments to do so. These results are consistent with prior studies of the medical and behavioral impact of commercial DTC genetic testing of common polygenic conditions: the PGen Study found that 35% of respondents reported sharing their results with a healthcare provider [58], and similar proportions were reported in other DTC genetic testing studies [59, 60]. This is slightly lower than the 51.2% who reported discussing their results with a healthcare provider in the current study, and could be due to differences in the complexity and scope of results that may be returned with genome sequencing compared to genetic testing for common conditions or the role of healthcare providers in ordering sequencing in some of the collaborating projects. An even higher percentage of participants (79.2%) from the YPO and MD/PhD Genome Projects reported discussing their results with a healthcare provider compared to the other projects, and this could be due to more encouragement from the study stuff to have these discussions [17]. While most providers were willing to discuss the PPGS results, longer follow-up will reveal if and how the results are integrated into longer-term care and clinical decision-making. We found some dissatisfaction in participants’ discussions about their PPGS results with their provider, even for participants from UYG which sent clinical genome reports directly to the ordering providers. Prior research has revealed that non-geneticist physicians feel unprepared for sequencing, unsure of their genomic knowledge and ability to interpret results [61–63]. A major concern is that the complexities of genome sequencing could result in a substantial number of individuals being falsely identified as at-risk for disease [6, 7]. This could create distress or a fatalistic response on the individual level, iatrogenic harm from unnecessary surveillance or procedures, or overutilization of healthcare resources [9]. Alternatively, individuals without significant findings may misinterpret those negative results as an indication that they are at no risk, and could have a false sense of security and not seek appropriate medical care.

Participant-reported personal utility or value of PPGS was high, with the perceived utility in the future significantly outranking current utility. Most participants reported satisfaction with their sequencing experience, and very few reported any decisional regret. Additionally, almost a quarter of participants both strongly agreed that personal genomic information should be part of the medical record and strongly disagreed that sequencing should only be available through a doctor, indicating comfort with access to sequencing outside of the healthcare system, but a perceived utility of the results for their medical care. Despite high overall satisfaction with sequencing, more than half of participants were disappointed that their results did not provide them with more information, a finding also observed in clinical settings and with DTC genetic testing [64, 65]. These high expectations of sequencing may reflect a lack of understanding of the current capabilities of this technology and the role of genetics (compared to lifestyle and environmental factors) on health. Though concerns have often focused on disclosure of unanticipated findings, these unrealistically high expectations of genomics should also be appropriately addressed in advance of disclosure of genomic results.

While experts and professional societies recommend caution around incorporating PPGS into standard clinical practice as a screening tool, sequencing is more economically and logistically feasible, and commercial ventures are responding to public interest by providing both true DTC, consumer-facing physician mediated, and traditional provider-mediated genomic products. As interest in PPGS grows, the need to address the gaps in our understanding of the implications of PPGS in healthy individuals becomes more urgent. Results from the PeopleSeq Consortium thus far do not appear to support many of the current concerns regarding the negative consequences of PPGS. Participants reported feelings of empowerment, and very few reported any distress or regret after receiving their personal genomic information. The participants reported sharing their genomic results with their healthcare providers, and the Consortium continues to collect data on the medical responses to quantify the utility or disutility of sequencing.

The strengths of the PeopleSeq Consortium include its longitudinal design, which allows for the collection of both short- and long-term outcomes of PPGS, and the breadth of behavioral and medical data collected. Furthermore, these outcomes are being examined across both research and commercial PPGS projects that have not been previously explored in the literature. As the Consortium is a collaboration of multiple PPGS projects with different protocols, there is a heterogeneous sequencing experience; however, this captures the current access points of PPGS and enables the exploration of PPGS experiences across different approaches to the return of genomic results. These findings then provide a summary of the outcomes of sequencing across the currently available means by which individuals can pursue PPGS. There are limitations to the study. The response rate is currently below 50%, but methods are being implemented to increase the proportion of responders moving forward. The results could be affected by nonresponder bias; however, the demographic differences observed between responders and nonresponders, while statistically significantly different, were not large and were similar to trends found in other studies of nonresponse [66]. Additionally, we currently do not have information on the individual genomic results that each participant received, and responses may vary depending on the content of the personal genomic reports. Thus, these findings are a summary of the responses and attitudes of individuals to PPGS results in general. Lastly, these results may not be generalizable, as early adopters of PPGS, like innovators and early adopters of most technologies, are different than the general public [33], notably being less diverse and potentially having greater risk-taking tendencies. Yet, little is currently known about the outcomes or attitudes of anyone undergoing PPGS and receiving their personal genomic results, and these individuals are likely to be representative of the innovators and early adopters in our society who are shaping the use of genomic technologies and much can be learned from their experience.

The Consortium is continuing to add additional projects, enroll additional participants, and administer annual follow-up surveys beyond the initial results reported on here. As participants report sequencing-related healthcare use over time, the associated costs will also be estimated and evaluated. This will answer questions on the economic impact of sequencing in healthy individuals, given concerns regarding the possible burden of sequencing on the healthcare system. Furthermore, participants’ genomic result reports are being collected by the Consortium, which will provide for rich analyses of outcomes as they relate to the actual genomic results the participants received. Additional strategies are being incorporated to improve the participant response rate and avoid attrition over longer-term follow-up.

## Conclusions

Here we have reported on the design and implementation of the PeopleSeq Consortium, a collaboration collecting and examining the experiences and medical, behavioral, and economic outcomes of PPGS in healthy individuals. These healthy individuals who underwent predispositional sequencing were not deterred by concerns of privacy of their genomic information or possible insurance discrimination. Participants were enthusiastic about their experience and not distressed by their results. Many participants reported value in their health-related results, and approximately half reported discussing their results with a healthcare provider; though few participants reported making medical or lifestyle changes. The participants in the PeopleSeq Consortium are early adopters of PPGS and are providing novel information on the attitudes and outcomes of current users of PPGS.

## Additional files

Additional file 1: **Table S1.** Overview of projects in the PeopleSeq Consortium included in this analysis. **Table S2.** Study measures by time point in the PeopleSeq Consortium included in this analysis. **Figure S1.** PeopleSeq Consortium enrollment and data collection by project. **Table S3.** Reported psychological, behavioral, and medical reactions following disclosure of genome sequencing results by respondents to the post-disclosure survey and catch-up survey. **Table S4.** Degree of agreement/disagreement on perceived utility and general attitudes regarding genome sequencing by respondents to the post-disclosure survey and catch-up survey. **Figure S2.** Distributions of demographic characteristics by responders and nonresponders in a substudy of the PeopleSeq Consortium. **Table S5.** Characteristics of participants with completed post-disclosure or catch-up surveys in the PeopleSeq Consortium by project. (PDF, 631.7 KB). (PDF 631 kb)

Additional file 2: The PeopleSeq Consortium surveys (pre-disclosure, post-disclosure, and catch-up surveys). (PDF, 1.4 MB). (PDF 1465 kb)

## Abbreviations

CLIA: Clinical Laboratory Improvement Amendments
DTC: Direct-to-consumer
PeopleSeq: Personal Genome Sequencing Outcomes Consortium
PGen: Impact of Personal Genomics Study
PGP: Harvard Personal Genome Project
PMI: Precision Medicine Initiative
PPGS: Predispositional personal genome sequencing
UYG: Illumina’s Understand Your Genome^Ⓡ^
YPO: Baylor College of Medicine’s Young Presidents’ Organization Genome Project
