# Supplementary material for: Predispositional genome sequencing in healthy adults: design, participant characteristics, and early outcomes of the PeopleSeq Consortium
Source: Genome Med. 2019 Feb 27;11:10. doi: 10.1186/s13073-019-0619-9 (PMC6391825; doi:10.1186/s13073-019-0619-9)
Supplement: Supplementary file 1 — Table S1. Overview of projects in the PeopleSeq Consortium included in this analysis. Table S2. Study measures by time point in the PeopleSeq Consortium included in this analysis. Figure S1. PeopleSeq Consortium enrollment and data collection by project. Table S3. Reported psychological, behavioral, and medical reactions following disclosure of genome sequencing results by respondents to the post-disclosure survey and catch-up survey. Table S4. Degree of agreement/disagreement on perceived utility and general attitudes regarding genome sequencing by respondents to the post-disclosure survey and catch-up survey. Figure S2. Distributions of demographic characteristics by responders and nonresponders in a substudy of the PeopleSeq Consortium. Table S5. Characteristics of participants with completed post-disclosure or catch-up surveys in the PeopleSeq Consortium by project. (PDF, 631.7 KB). (PDF 631 kb) [file 13073_2019_619_MOESM1_ESM.pdf]

Additional file 1

Predispositional Genome Sequencing in Healthy Adults: Design, Participant Characteristics, and Early Outcomes of the PeopleSeq Consortium

Supplementary Tables and Figures

Table S1. Overview of projects in the PeopleSeq Consortium included in this analysis

Table S2. Study measures by time point in the PeopleSeq Consortium included in this analysis

Figure S1. PeopleSeq Consortium enrollment and data collection by project

Table S3. Reported psychological, behavioral, and medical reactions following disclosure of genome sequencing results by respondents to the post-disclosure survey and catch-up survey

Table S4. Degree of agreement/disagreement on perceived utility and general attitudes regarding genome sequencing by respondents to the post-disclosure survey and catch-up survey

Figure S2. Distributions of demographic characteristics by responders and nonresponders in a substudy of the PeopleSeq Consortium

Table S5. Characteristics of participants with completed post-disclosure or catch-up surveys in the PeopleSeq Consortium by project

Table S1. Overview of projects in the PeopleSeq Consortium included in this analysis

Site	Setting	Overview	Platform	Results returned	Raw data returned	Pre-test genetic counseling	Method of results disclosure	Cost (median; 10th-90th percentile)
**HealthSeq** (Mount Sinai) [1-3]	Academic	Longitudinal cohort study of 40 adults recruited from the general population at the Mount Sinai Medical Center in New York City between 2012-2015.	WGS	• Monogenic disease risk for rare disease-associated variants<br>• Common disease risk<br>• Pharmacogenomics for response to 3 drugs<br>• Non-health-related results<br>Participants could decline classes of results	BAM files of aligned sequence reads, VCF files of variants	Provided by GC as part of research protocol	By study MG, GC with non-clinical report	None
**Personal Genome Project** (Harvard Medical School) [4-7]	Academic	Longitudinal cohort study which began recruitment in 2005 with over 5,400 adults enrolled; approximately 351 have had their genome sequenced and returned to date. Participants must correctly complete an enrollment examination of human subjects research and basic genetics. Participants must also agree to an open consent in which any genome and health record data provided can be included in an open access public database with no guarantee on anonymity.	WGS	Filtered variants with literature [8]	Variants	None	Online profile with semi-automated non-clinical report	None (donation suggested)
**Understand Your Genome**® (Illumina) [9]	Industry	Provides commercial sequencing in conjunction with a symposium at sites around the world. A participant's physician orders sequencing and then the participant attends an educational symposium.	WGS, CLIA-certified laboratory	• Monogenic disease risk for over 1,200 medical conditions<br>• Pharmacogenomics for response to 16 drugs<br>Participants could minimally customize results they received and were provided access to an online genome visualization tool	VCF files of variants (returned at discretion of ordering HCP or by request without HCP)	Provided by ordering HCP	Online tool introduced at UYG symposium. Clinical report sent to ordering HCP.	\$3,000 (\$2,000-\$5,000)
**Young Presidents' Organization and MD/PhD Genome Projects** (Baylor College of Medicine) [10]	Academic	A cohort of 130 adults recruited from educational genomics seminars in Houston, Texas in 2011 and 2013.	WES	• Monogenic disease risk for rare disease-associated variants found in HGMD and predicted to be damaging to protein function	None	Provided by MD as part of research protocol	By study MD, MG with non-clinical report	None

WGS, whole genome sequencing; GC, genetic counselor; MG, medical geneticist; CLIA, Clinical Laboratory Improvement Amendments; HCP, healthcare provider; UYG, Understand Your Genome; WES, whole exome sequencing; HGMD, Human Genome Mutation Database; MD, physician

Supplementary Table S1 References

1. Sanderson SC, Linderman MD, Suckiel SA, Diaz GA, Zinberg RE, Ferryman K, et al. Motivations, concerns and preferences of personal genome sequencing research participants: Baseline findings from the HealthSeq project. Eur J Hum Genet. 2016;24(1):14-20.
2. Sanderson SC, Linderman MD, Suckiel SA, Zinberg R, Wasserstein M, Kasarskis A, et al. Psychological and behavioural impact of returning personal results from whole-genome sequencing: the HealthSeq project. Eur J Hum Genet. 2017;25(3):280-92.
3. Suckiel SA, Linderman MD, Sanderson SC, Diaz GA, Wasserstein M, Kasarskis A, et al. Impact of genomic counseling on informed decision-making among ostensibly healthy individuals seeking personal genome sequencing: the HealthSeq Project. J Genet Couns. 2016;25(5):1044-53.
4. Church GM. The personal genome project. Mol Syst Biol. 2005;1:2005.0030.
5. Lunshof JE, Bobe J, Aach J, Angrist M, Thakuria JV, Vorhaus DB, et al. Personal genomes in progress: from the human genome project to the personal genome project. Dialogues Clin Neurosci. 2010;12(1):47-60.
6. Ball MP, Bobe JR, Chou MF, Clegg T, Estep PW, Lunshof JE, et al. Harvard Personal Genome Project: lessons from participatory public research. Genome Med. 2014;6(2):10.
7. Personal Genome Project. http://personalgenomes.org/. Accessed 20 Aug 2018.
8. Ball MP, Thakuria JV, Zaranek AW, Clegg T, Rosenbaum AM, Wu X, et al. A public resource facilitating clinical use of genomes. Proc Natl Acad Sci U S A. 2012;109(30):11920-7.
9. Understand Your Genome. https://www.understandyourgenome.com/. Accessed 20 Aug 2018.
10. Gonzalez-Garay ML, McGuire AL, Pereira S, Caskey CT. Personalized genomic disease risk of volunteers. Proc Natl Acad Sci U S A. 2013;110(42):16957-62.

Table S2. Study measures by time point in the PeopleSeq Consortium included in this analysis

	Pre-disclosure survey	Post-disclosure survey	Catch-up survey
Personal characteristics			
Sociodemographic characteristics	X		X
Genome sequencing knowledge	X	X	X
Personal health information			
Personal & family health history	X	X	X
Prior use of genetics/genomics	X		X
Genomic test results[a]		X	X
Decision to pursue predispositional sequencing			
Decision process	X		X
Motivations	X		X
Psychological impact			
Anxiety	X	X	X
Test-related distress or regret	X	X	X
Risk perceptions			
Risk perceptions	X	X	X
Comprehension of results		X	X
Personal utility			
Fulfilled expectations		X	X
Satisfaction with information		X	X
Behavioral responses			
Communication of results		X	X
Information-seeking		X	X
Health behavior changes		X	X
Insurance changes		X	X
Medical responses			
Discussion with healthcare providers		X	X
Medical tests or exams		X	X
Medication changes		X	X

[a]Information also obtained directly from collaborating projects

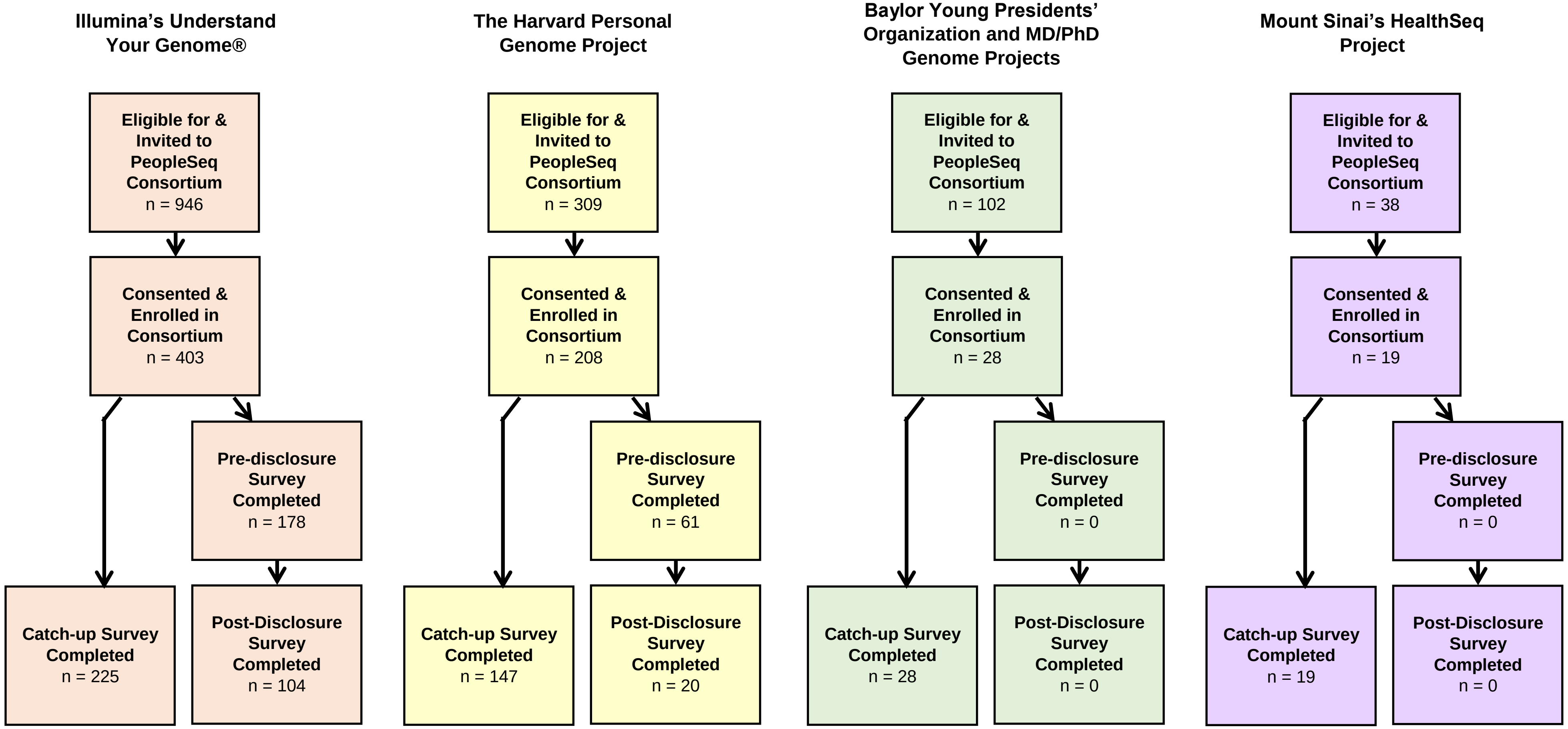

Figure S1. PeopleSeq Consortium enrollment and data collection by project

Table S3. Reported psychological, behavioral, and medical reactions following disclosure of genome sequencing results by respondents to the post-disclosure survey and catch-up survey

	**No. (%)[a]**	
	Post-disclosure survey[b]	Catch-up survey[b]
**Psychological reaction**		
Decision regret score, mean (±SD; range)[c]	6.3 (10.8; 0-75)	6.7 (14.1; 0-100)
**Behavioral and medical responses because of sequencing results**		
Communication of test results		
Family	91 (85.9)	308 (79.8)
Healthcare provider	55 (51.9)	197 (51.0)
Made appointment with healthcare provider	13 (12.4)	52 (13.8)
Sought out more information about health or medical topics related to results	50 (47.2)	187 (48.8)
Made changes to diet	6 (5.7)	39 (10.4)
Made changes to exercise routine	6 (5.8)	35 (9.4)
Made changes to medications	7 (6.6)	22 (7.4)
Made changes to insurance coverage	0 (0)	2 (0.5)

SD, standard deviation

[a]Percentages and means are not all based on total number of participants (124 for the post-disclosure survey and 419 for the catch-up survey) because of missing responses to some survey items. The percent of missing responses ranges between 14.5-16.1% (median=14.5% missing) for the post-disclosure survey and 6.7-29.1% (median=9.8%) for the catch-up survey.

[b]There were no statistically significant differences in any of the variables by survey (post-disclosure survey and catch-up survey) using the Wilcoxon rank sum test for the continuous variable and Chi square test or Fisher's exact test for categorical variables ($p>0.05$ for all).

[c]5-item decision regret scale provides a score from 0-100.

Table S4. Degree of agreement/disagreement on perceived utility and general attitudes regarding genome sequencing by respondents to the post-disclosure survey and catch-up survey

	No. (%)[a]				
	Strongly disagree	Somewhat disagree	Neither agree nor disagree	Somewhat agree	Strongly agree
**Post-disclosure survey**					
**Perceived utility of genome sequencing**					
I learned something to improve my health that I didn't know before	13 (12.4)	23 (21.9)	36 (34.3)	25 (23.8)	8 (7.6)
Having personal genome sequencing made me feel like I have more control over my health	3 (2.9)	10 (9.5)	22 (21.0)	51 (48.6)	19 (18.1)
What I learned from my personal genome sequencing will help reduce my chances of getting sick	10 (9.6)	33 (31.7)	35 (33.7)	21 (20.2)	5 (4.8)
The information that I received about my genome will influence how I manage my health in the future	6 (5.8)	16 (15.4)	39 (37.5)	38 (36.5)	5 (4.8)
I am disappointed that my results did not tell me more information	14 (13.3)	19 (18.1)	18 (17.1)	40 (38.1)	14 (13.3)
**Attitudes regarding genome sequencing**					
Personal genomic information should be part of a standard medical record[b]	1 (1.0)	8 (7.7)	19 (18.3)	35 (33.7)	41 (39.4)
Health insurance should cover personal genome sequencing	4 (3.9)	9 (8.7)	26 (25.0)	32 (30.8)	33 (31.7)
Personal genome sequencing should only be available to people through their doctor	28 (26.9)	29 (27.9)	16 (15.4)	18 (17.3)	13 (12.5)
**Catch-up survey**					
**Perceived utility of genome sequencing**					
I learned something to improve my health that I didn't know before	57 (15.3)	63 (16.9)	97 (26.0)	80 (21.5)	76 (20.4)
Having personal genome sequencing made me feel like I have more control over my health	39 (10.3)	34 (9.0)	93 (24.6)	130 (34.4)	82 (21.7)
What I learned from my personal genome sequencing will help reduce my chances of getting sick	73 (19.3)	71 (18.8)	140 (37.0)	57 (15.1)	37 (9.8)
The information that I received about my genome will influence how I manage my health in the future	58 (15.3)	34 (9.0)	98 (25.9)	133 (35.2)	55 (14.6)
I am disappointed that my results did not tell me more information	54 (14.4)	44 (11.7)	69 (18.4)	116 (30.9)	92 (24.5)
**Attitudes regarding genome sequencing**					
Personal genomic information should be part of a standard medical record[b]	6 (1.6)	26 (7.1)	35 (9.5)	117 (31.8)	184 (50.0)
Health insurance should cover personal genome sequencing	22 (5.9)	47 (12.6)	69 (18.6)	96 (25.8)	138 (37.1)
Personal genome sequencing should only be available to people through their doctor	149 (40.3)	80 (21.6)	46 (12.4)	47 (12.7)	48 (13.0)

[a]Percentages may not sum to 100 due to rounding. Percentages are not all based on denominator of 124 for the post-disclosure survey and 419 for the catch-up survey because of missing responses to some survey items. The percent of missing responses ranges between 15.3-16.1% (median=16.1% missing) for the post-disclosure survey and 9.8-12.2% (median=10.7% missing) for the catch-up survey.

[b]There was a statistically significant difference in the distribution of this variable between the post-disclosure survey and catch-up survey using the Wilcoxon rank sum test (p=0.040). There were no statistically significant differences in the distribution of any of the other variables by survey (p>0.05).

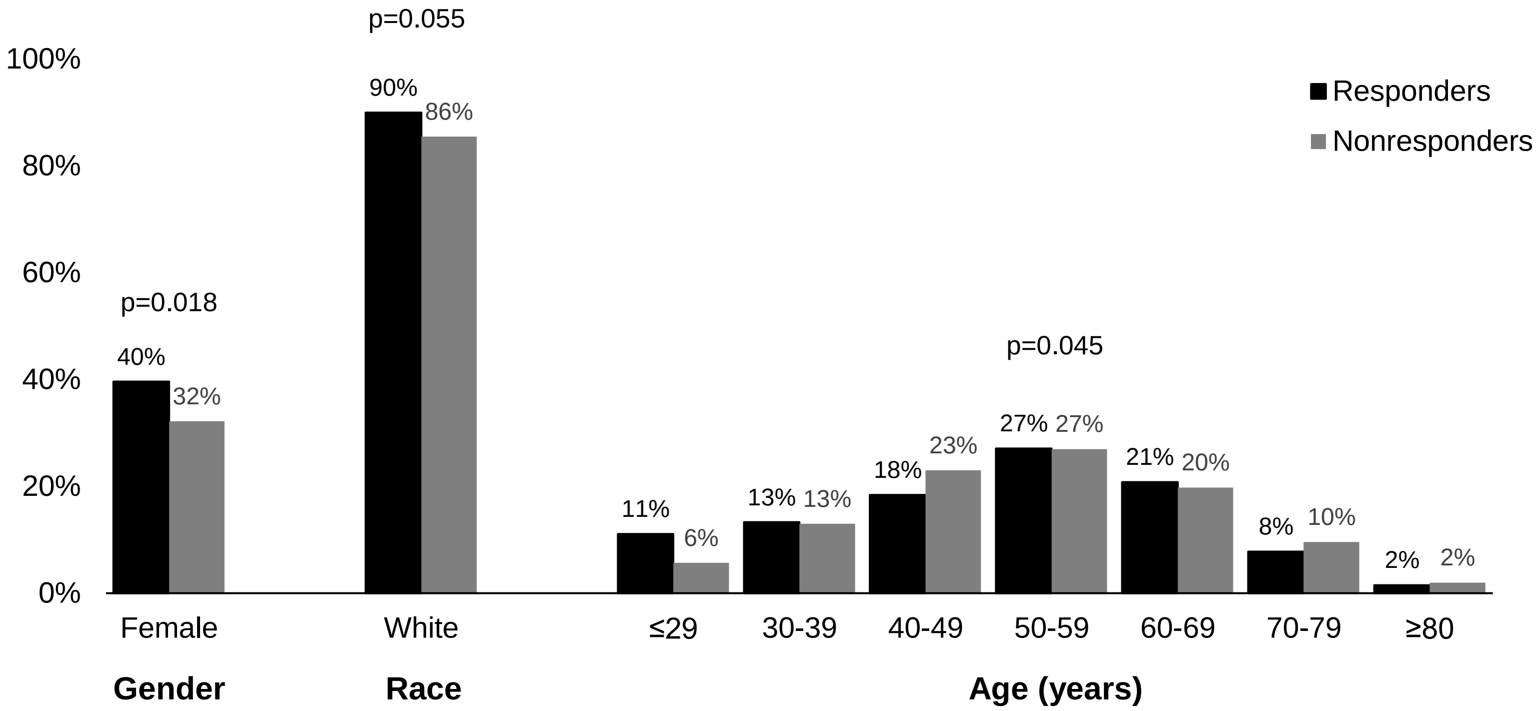

Figure S2. Distributions of demographic characteristics by responders (n=336, in black) and nonresponders (n=757, in gray) in a substudy of the PeopleSeq Consortium. *P* values for the differences in the distributions of gender, race, and age by response status (responders and nonresponders) from Chi-square tests.

Table S5. Characteristics of participants with completed post-disclosure or catch-up surveys in the PeopleSeq Consortium by project (n=543)[a]

Characteristic	Illumina's Understand Your Genome	Harvard Personal Genome Project	Baylor Young Presidents' Organization and MD/PhD Genome Projects	Mount Sinai's HealthSeq project	*P* value[b]
N	329	167	28	19	
Age, mean (SD), years	53.5 (11.6)	50.9 (14.9)	62.0 (11.1)	52.4 (11.2)	<0.001
Female, %	38.8	38.0	29.6	36.8	0.824
White, %	90.7	93.2	96.3	89.5	0.671
Hispanic or Latino, %	3.4	2.5	0	5.3	0.675
> College education, %	88.2	69.6	88.9	94.7	<0.001
Annual income ≥$100,000, %	84.3	59.5	100.0	73.7	<0.001
Married, %	80.8	55.6	96.4	26.3	<0.001
Biological children, %	75.2	54.9	100.0	52.6	<0.001
United States resident, %	81.4	98.8	100.0	100.0	<0.001
Self-reported good health or better, %	97.9	92.0	100.0	94.1	0.018
Prior genetic testing, %	47.4	62.8	4.2	41.2	<0.001

SD, standard deviation

[a]Percentages may not sum to 100 due to rounding. Percentages and means are not all based on total n for each project because of missing responses to some survey items. The percent of missing responses ranges between 0-12.7% (median=2.4% missing).

[b]*P* value for differences across projects using ANOVA for continuous variables and Chi-square test or Fisher's exact test for categorical variables.
