# Supplementary material for: Predispositional genome sequencing in healthy adults: design, participant characteristics, and early outcomes of the PeopleSeq Consortium
Source: Genome Med. 2019 Feb 27;11:10. doi: 10.1186/s13073-019-0619-9 (PMC6391825; doi:10.1186/s13073-019-0619-9)
Supplement: Supplementary file 2 — The PeopleSeq Consortium surveys (pre-disclosure, post-disclosure, and catch-up surveys). (PDF, 1.4 MB). (PDF 1465 kb) [file 13073_2019_619_MOESM2_ESM.pdf]

Additional file 2

Predispositional Genome Sequencing in Healthy Adults: Design, Participant Characteristics, and Early Outcomes of the PeopleSeq Consortium

PeopleSeq Consoritum Surveys

1. PeopleSeq pre-disclosure survey
2. PeopleSeq post-disclosure survey
3. PeopleSeq catch-up survey

PeopleSeq Consortium
Pre-disclosure Survey

PEOPLESEQ PRE-DISCLOSURE QUESTIONNAIRE SPECIFICATIONS MASTER

Short URL to direct towards production survey	https://www.ssgresearch.com/peopleseq
Support email address to include in header	peopleseq@ssgresearch.com
Support phone number to include in header (if needed)	

Logo to use if other than SSG logo
Please list network location of other logo to use: GDrive/S15002 PeopleSeq 2015/DEFLOG.gif

---

Mandatoriness (check the appropriate setting)

X All questions are optional unless otherwise noted
All questions are optional with a soft prompt included if no answer is provided
[OTHER SPECIFY FIELDS SHOULD ALSO HAVE SOFT PROMPTS]
All questions are mandatory

Please provide text to use for Mandatoriness prompt if being used (Default text to use is provided below):

GENERAL SOFT PROMPT:

We noticed that you did not answer a question on the previous page. It is important to us that we get a complete set of responses from you. Please return to the previous page by clicking "Previous" and select an answer for each question. If you would rather not select an answer, you may instead continue to the next page by clicking "Next."

OTHER SPECIFY SOFT PROMPT:

You selected 'Other' but did not specify your answer. Please return to the last question by clicking "Previous" and type in your specific answer. If you would rather not specify an answer, you may instead continue to the next page by clicking "Next."

Section Headers

Section Label	Section Start Page #
1. **About You**	5
2. **Your Decision**	12
3. **Your Genome Results Report: Information**	17
4. **Your Genome Results Report: Attitudes**	23
5. **Your Entire Genome Sequence**	25
6. **General Attitudes Towards Genomics**	26
7. **A Few More Questions About You**	27
8. **Wrap-Up**	39

Survey Title to Appear in Header (appears above the section header bar)

The Personal Genome Sequencing Outcomes Study

Welcome Page Text (modify the following as needed)

Welcome to the Personal Genome Sequencing Outcomes Study!

This is one of the first large-scale systematic studies to examine the experiences, attitudes, and outcomes of healthy adults who have chosen to pursue personal genome sequencing. Our hope is that the knowledge gained through this study will play an integral role in shaping the development of genome sequencing policy and its integration into health care.

This is the first survey for this study!

{PRG: IF AUTO-LOGIN PROTOCOL IS BEING USED, INSERT THE FOLLOWING TEXT}
Please click Start Survey to begin!

Resume Page Text (modify the following as needed)

Thank you for returning to the survey. Please click “resume” to begin where you last left off…

Terminate Text (modify the following as needed)

Thank you for considering participation in this study. Participation requires that you provide consent. We will not contact you again about this study. If you believe you received this message in error, please contact us at the email in the upper right corner of the screen so that we may reset your case.

End Page Text

Thank you for your participation! This concludes the first study survey. We will email you with a link to the second study survey after you receive your personal genome sequencing results.

For information about the Personal Genome Sequencing Outcomes Study, including contact details, please refer to the study website at: http://www.genomes2people.org/the-peopleseq-study/ {PRG: Set link to open in new window.}

Results of the Personal Genome Sequencing Outcomes Study will be posted on the study website as they become available.

We really appreciate the time you have taken to complete this survey. You will now be entered into a sweepstakes drawing for an Apple Watch upon completion of this study, and we will be in touch to let you know if you are the winner!

You may now close your browser.

Survey Title Appearing in Browser Window

Personal Genome Sequencing Outcomes Study

GENERAL PROGRAMMING NOTES

For confidentiality purposes, this questionnaire will be programmed as three separate surveys. Survey A will be the consent form, some basic contact information, and a few demographics. Survey B will be the main body of the questionnaire. And Survey C will be asking for additional contact information.

Please pass all demographics collected in Survey A into Survey B and store them in a calculation there – to facilitate data processing/review. Do NOT pass identifiable data, except for DATSTAT_ALTPID.

All emphasis should be programmed in black, all caps text instead of lowercase blue text.

Lines indicate page breaks throughout the survey and show which should questions appear together.

PRELOADS

PRE_1 Genome Sequencing Program

1 UYG
2 PGP
3 HealthSeq
4 CEO Genome Project
5 MD/PhD Genome Project
6 SOAPS
7 Pioneer 100
8. UVM UYG
9. Invitae
10 Hudson Alpha

{NOTE: PRE_0 IS A CALCULATION DRIVEN OFF OF PRE_1 - **IT SHOULD NOT BE PRELOADED}**
PRE_0 Genome Sequencing Program (with Acronym)

1 Understand Your Genome (UYG)
2 The Harvard Personal Genome Project (PGP)
3 the Whole Genome Sequencing in the Clinical Setting (HealthSeq) Study at Mount Sinai
4 CEO Genome Project
5 MD/PhD Genome Project
6 UNLV Survey of Opinions about Personalized-Medicine Services (SOAPS)
7 Pioneer 100
8. UVM UYG
9. Invitae
10 Hudson Alpha

{PRG: START SURVEY A}

CONSENT

{NOTE: Center first two lines on screen; NO scrolling text box for consent; add PDF link at top of screen (separate PDFs for each company)}

Welcome to the Personal Genome Sequencing Outcomes Study!

Please read this consent form, and then make one of the choices below.

INSERT CONSENT LANGUAGE HERE {PRG: Final Consent language coming after initial programming. Consent form will dynamically change depending on the value of PRE_1}

{NOTE: Add space between consent options}
{PRG: TIMESTAMP UPON QCONSENT SUBMISSION}
{PRG: QCONSENT REQUIRED}

QCONSENT. Please read the consent form above, and then select one of the choices below. Please enter your first and last name below as your signature confirming your selection, and then click "Next" to proceed.

1 I have read this consent form, and I agree to the study procedures described above. I confirm that I am 18 years or older. I confirm that I have signed up for personal genome sequencing through {DISPLAY PRE_0}, and I have received an email inviting me to participate in this research study.
2 I do NOT wish to participate in this study.

DEM1C: Title

2 Dr.
3 Ms.
4 Mrs.
5 Mr.
6 Miss
7 Mx.
8 Hon

{DEM1A & DEM1B ARE REQUIRED IF QCONSENT=1}

DEM1A: First Name

DEM1B: Last Name

{PRG: IF QCONSENT=2, SUBMIT CASE AS TERMINATE. DO NOT SEND THANK YOU EMAIL NOTED ON NEXT PAGE}

{Note: Section Header 1: About You}

SECTION INTRO: *First, we would like to ask a few questions about you to help us analyze the results of the survey, and so that we can contact you again for future surveys. Please note that the first five questions are required in order to participate. We will only use your email address to contact you as part of this study; we will never use this information for any other purpose.*

{PRG: SAME SCREEN DEM2-DEM5}
{PRG: MAKE DEM2-DEM5 REQUIRED – ADD RED STAR TO INDICATE REQUIREMENT}
{PRG: VALIDATE EMAIL ADDRESSES CONTAINS @ and}

DEM2. What is your email address? *(Please enter in the format email@address.com)*

Email address: [EMAIL ADDRESS]

DEM3. What year were you born in?

[NUMERIC 1894-1997]

DEM4a. Which gender do you identify as?

1 Female
2 Male
3 Other *(Please specify)*: [TEXT RESPONSE]

DEM4b. What was your anatomical sex at birth?

1 Female
2 Male
3 Intersex or other *(Please specify):* [TEXT RESPONSE]
4 Choose not to answer

DEM5. Where do you currently live?

1 Within the United States
2 Outside the United States *(Please specify country)*: [TEXT RESPONSE]

Invitae Participant Note {PRG: DO NOT SHOW}
{PRG: SHOW ON ALL PAGES IF PRE_1=9 (INVITAE)}
Invitae Participants: In this survey, ***genome*** refers to ***genetic***, ***genome sequencing*** refers to ***genetic testing***, and ***genome reports*** refer to ***genetic test reports***.

FQ1. Have you received your {DISPLAY PRE_0} personal genome sequencing results?

1 Yes
2 No

{PRG: SHOW FQ2 IF FQ1=1, OTHERWISE SKIP TO SURVEY B}

FQ2. When did you first receive your {RESPONSE:PRE_1} results (i.e., your personal genome sequencing results report)? *(If you are unsure, please provide your best estimate.)*

AD1_MONTH: Drop-Down: (1=Jan through 12=Dec)
AD1_YEAR: Drop-Down: (2000 through 2015)

{PRG: IF FQ1=2 SEND ON TO PRE SURVEY. SHOW FOLLOWING STATEMENT IF FQ1=1 AND DATE IS PRIOR TO CURRENT DATE}

Thank you for agreeing to participate in the Personal Genome Sequencing Outcomes Study! Since you have already received your personal genome sequencing results we would like to follow-up with you in a few months time to complete the rest of this survey.

{SUBMIT SURVEY A AND EMAIL ALERT} Send email to R thanking them for agreeing to participate and giving them a direct link to access the main survey instrument. This is to allow Rs to leave main survey and return later if desired. Include link to a copy of the consent form.

TEXT. *Next, we would like to ask a few more questions about you.*

Invitae Intro Note {PRG: DO NOT SHOW}
{PRG: SHOW ONLY IF PRE_1=9 (INVITAE)}

NOTE TO INVITAE PARTICIPANTS:

Some terminology used in the survey questions may differ from terms you and your health care professional have used when discussing your Invitae genetic testing and/or your test results. A note is provided at the top of each page of the survey that describes how different terms should be defined; please refer to this note if any terms in the questions are unfamiliar to you.

{PRG: K2 USE GENERAL SOFT PROMPT}

K2. What is your marital status?

1 Married
2 Widowed
3 Divorced
4 Separated
5 Never married

{PRG: SHOW K2A IF K2=2,3,4,5}

K2a. Are you currently living with a boyfriend/girlfriend or spouse/partner?

1 Yes
2 No

{PRG: K1 USE GENERAL SOFT PROMPT}

K1. Are you adopted?

1 Yes
0 No
2 Not sure

{PRG: K3 USE GENERAL SOFT PROMPT}

K3. How many children (including both biological and non-biological) do you have? *(Enter "0" if you have none.)*

[NUMERIC ENTRY 0-30]

{PRG: SHOW K3a IF K3>0, OTHERWISE SKIP TO K5}
{PRG: K3a USE GENERAL SOFT PROMPT}

K3a. How many biological children do you have? *(Enter "0" if you have none.)*

[NUMERIC ENTRY 0-30]

{PRG: SHOW K3b if K3a>0, OTHERWISE SKIP TO K5}
{PRG: K3b USE GENERAL SOFT PROMPT}

K3b. Are any of your biological children less than 18 years old?

1 Yes
0 No

{PRG: SHOW K3B_1 ONLY IF PRE_1=9 (INVITAE); OTHERWISE GO TO FILTERS BEFORE K5}

K3b_1. Including yourself, how many people are in your **nuclear family**? *A nuclear family is a partnered adult couple or a single adult and all dependent children who live together at the same address.*

[DISCRETE NUMERIC RESPONSE 1-100]

K3b_2. Including yourself, how many people are in your **extended family**? *An extended family includes a nuclear family plus grandparents, aunts, uncles and/or other relatives who live together in the same household.*

[DISCRETE NUMERIC RESPONSE 1-100]

K3b_3. Do most of your family members who do not live in your household, reside within one hour's drive of your home?

1 Yes
2 No
3 Not sure

K3b_4. Do you typically share your personal health information with any other individuals in your nuclear or extended family?

1 Yes
2 No

{PRG: IF K3b_3=1, ASK K3b_3a; OTHERWISE GO TO K5}

K3b_5. With whom do you typically share your personal health information? *(Select all that apply.)*

1 Spouse/partner
2 Children
3 Parents
4 Other relatives
5 Other (Specify): [TEXT RESPONSE]

{PRG: K5 - SELECT ALL THAT APPLY}
{PRG: K5 USE GENERAL SOFT PROMPT}
{PRG: K5.8.TEXT USE OTHER SPECIFY SOFT PROMPT}
{DESIGN: K5/K4 ON SAME PAGE}

K5. Which one or more of the following would you say is your race? (*Please select all that apply*.)

1 American Indian or Native Alaskan
2 Black or African American
3 East Asian
4 Hawaiian or other Pacific Islander
6 South Asian
7 White or Caucasian
8 Other *(Please specify):* [TEXT RESPONSE]

{PRG: K4 USE GENERAL SOFT PROMPT}

K4. Do you consider yourself Hispanic or Latina/o?

1 Yes
0 No

{PRG: SHOW QUESTION NOTE ON TOP OF PAGE IF PRE_1=9 (INVITAE)}
QUESTION NOTE: Due to the topic of the PeopleSeq study, this survey includes some questions that are of a sensitive nature. We greatly appreciate your open and honest responses to all questions - all data remains strictly confidential and anonymous; however, you are not required to respond to all questions.

K10. How religious do you consider yourself to be?

1 Not at all religious
2 Not very religious
3 Somewhat religious
4 Very religious

K11. Which of the following best describes your religion or worldview?

1 Agnostic
2 Atheist
3 Buddhist
4 Christian
5 Hindu
6 Jewish
7 Muslim
8 Protestant
9 Other *(Please specify):* [TEXT RESPONSE]

{PRG: K6 USE GENERAL SOFT PROMPT}

K6. What is the highest level of education you have completed?

1 Never attended school
2 Grade school (grades 1 to 8)
3 Some high school (grades 9 to 12)
4 High school graduate or GED
5 Post high school training other than college (vocational, technical, or other types of training)
6 Some college
7 Associate's degree
8 Bachelor's degree or equivalent
9 Some graduate school
10 Master's degree (MS, MBA, MFA, etc.)
11 Some doctoral work
12 Doctoral or other professional degree (MD, PhD, JD, or other)

{PRG: K7 USE GENERAL SOFT PROMPT}
{PRG: K7 SELECT ALL THAT APPLY}

K7. What is your current work situation? *(Please select all that apply.)*

1 Working full-time
2 Working part-time
3 Temporarily laid off, sick leave, or maternity leave
4 Retired
5 Self-employed
6 Looking for work, unemployed
7 Disabled, permanently or temporarily
8 Homemaker
9 Student

{PRG: K8 USE GENERAL SOFT PROMPT}
{PRG: K8.11.TEXT USE OTHER SPECIFY SOFT PROMPT}

K8. In which field do you now or did you most recently work? *(Please select all that apply.)*

1 Business, Financial, Management, Sales, and Related Occupations
2 Computer, Engineering, and Mathematical Science
3 Life, Physical, and Social Science
4 Legal
5 Education, Training, and Library
6 Arts, Design, Entertainment, Sports, and Media
7 Healthcare Provider or Clinical Researcher
8 Office and Administrative Support
9 Construction, Maintenance, and Natural Resources
10 Production and Transportation
11 Other *(Please specify):* [TEXT RESPONSE]

{PRG: SHOW IF K8=7; OTHERWISE GOTO K9}
{DESIGN: SHOW K8a & K8b ON SAME PAGE}
{PRG: K8a USE GENERAL SOFT PROMPT}
{PRG: K8a.9.TEXT USE OTHER SPECIFY SOFT PROMPT}

K8a. What is your educational background? *(Please select all that apply.)*

1 MD
2 PysD
3 PharmD
4 PhD
5 Master's in Genetic Counseling
6 Registered Nurse
7 Nurse Practitioner
8 Physician Assistant
9 Other *(Please specify):* [TEXT RESPONSE]

{PRG: K8b USE GENERAL SOFT PROMPT}
{PRG: K8b.11.TEXT USE OTHER SPECIFY SOFT PROMPT}

K8b. What is your primary clinical specialty?

1 Primary care
2 Genetics specialist (e.g., genetic counselor, clinical geneticist)
3 Anesthesiology
4 Nutrition
5 Obstetrics/gynecology
6 Oncology
7 Internal medicine
8 Surgery
9 Pediatrics
10 I do not have a specialty
11 Other specialty *(Please specify):* [TEXT RESPONSE]

K9. What is your household's total combined income during the past 12 months? *(This includes money from pensions, social security payments, jobs, net income from business, farm or rent, dividends, interest, and any other income received by family members who are 15 years of age or older. Options listed are in U.S. Dollars.)*

1 Less than $20,000
2 $20,000 – $39,999
3 $40,000 – $69,999
4 $70,000 – $99,999
5 $100,000 – $199,999
6 $200,000 – $500,000
7 More than $500,000

{PRG: OPTION 10 IS MUTUALLY EXCLUSIVE}

H1. Do you have health insurance or a health coverage plan? *(Please select all that apply.)*

1 Yes, through my employer
2 Yes, through someone else's employer
3 Yes, a plan that I or someone else buys
4 Yes, through Medicare
5 Yes, through Medicaid or Medical Assistance
6 Yes, through the military, CHAMPUS, or the VA
7 Yes, through the Indian Health Service or the Alaska Native Health Service
11 Yes, through other national or government sponsored health service
8 Yes, through some other source *(Please specify):* [TEXT RESPONSE]
9 No, I don't have any coverage
10 I don't know

H2-H4. Do you have…

1 Yes
0 No
99 I don't know

H2. Life insurance?
H3. Disability insurance?
H4. Long-term care insurance?

{PRG: SHOW H3a IF H3=1}

H3a. Is your disability insurance short term, long term, or both?

1 Short term
2 Long term
3 Both short and long term
99 I don't know

L1. Which of the following best applies to you? *(Please note cigarettes refer to tobacco and not electronic cigarettes.)*

1 I smoke cigarettes (including hand-rolled) every day.
2 I smoke cigarettes (including hand-rolled), but not every day.
3 I do not smoke cigarettes at all, but I do smoke tobacco of some kind (e.g., pipe or cigar).
4 I have stopped smoking completely in the last year.
5 I stopped smoking completely more than a year ago.
6 I have never been a smoker (i.e., smoked for a year or more).

{PRG: SHOW IF L1=1 OR 2, ELSE GOTO Q1}
L2. Are you seriously thinking of quitting smoking?

1 Yes, within the next 30 days
2 Yes, within the next 6 months
3 No, not thinking of quitting

{Note: Section Header 2: Your Decision}

Invitae Participant Note {PRG: DO NOT SHOW}
{PRG: SHOW ON ALL PAGES IF PRE_1=9 (INVITAE)}
Invitae Participants: In this survey, ***genome*** refers to ***genetic***, ***genome sequencing*** refers to ***genetic testing***, and ***genome reports*** refer to ***genetic test reports***.

SECTION INTRO: *In this section, we will be asking you some questions about the personal genome sequencing that you had done through {DISPLAY PRE_0}.*

First, please think about the process you went through when deciding whether or not to pursue personal genome sequencing through {DISPLAY PRE_1}.

Q1. How long did it take you to decide to pursue personal genome sequencing through {DISPLAY PRE_1}?

1 One day or less
2 More than one day but less than a week
3 Between one and two weeks
4 Several weeks (more than two weeks but less than a month)
5 Between one and two months
6 More than two months
7 Not sure

Q2. When making your decision, did you talk with anyone about whether or not to pursue personal genome sequencing through {DISPLAY PRE_1}?

1 Yes
0 No

{PRG: SHOW Q2a IF Q2=1; OTHERWISE SKIP TO Q6}
{PRG: SELECT ALL THAT APPLY}

Q2a. Whom did you talk to about whether or not to pursue personal genome sequencing through {DISPLAY PRE_1}? *(Please select all that apply.)*

1 Family members
2 Friends
3 Co-workers/colleagues
4 Primary care provider
5 Genetics specialist (e.g., genetic counselor or clinical geneticist)
6 Other healthcare provider(s)
7 Representative from [DISPLAY PRE_1]
8 Other *(Please specify)*: [TEXT RESPONSE]

MD1. Were you required to have a physician or healthcare provider order this personal genome sequencing through {DISPLAY PRE_1} for you?

1 Yes
2 No
3 I don't know

{PRG: SHOW MD2 IF MD1=1, OTHERWISE SKIP TO FILTER BEFORE B3b}

MD2. Did you have to approach more than one physician about ordering this personal genome sequencing through {DISPLAY PRE_1} for you?

1 Yes
2 No

MD3. How willing was the {PRG: IF MD2=1 SHOW "first"} physician or healthcare provider you approached to order this personal genome sequencing through {DISPLAY PRE_1} for you?

1 Not at all willing
2 Somewhat willing
3 Very willing

{PRG: SHOW MD4 IF MD2=1, OTHERWISE SKIP TO FILTER BEFORE B3b}

MD4. How willing was your ordering physician or healthcare provider to order this personal genome sequencing through {DISPLAY PRE_1} for you?

1 Not at all willing
2 Somewhat willing
3 Very willing

{PRG: SHOW B3b IF Q2a=4 OR 5 OR 6, OTHERWISE SKIP TO Q6}

B3b. Are you required to consult with a healthcare provider before you receive your personal genome sequencing results from {DISPLAY PRE_1}?

1 Yes
2 No
3 I don't know

Q6. Did you pay for the personal genome sequencing you will receive through {DISPLAY PRE_1}?

1 Yes
2 No

{PRG: SHOW IF Q6=1; OTHERWISE GOTO B1}

Q6a. How much did you pay for the personal genome sequencing you will receive through {DISPLAY PRE_1}? *(Please estimate your genetic testing cost in in U.S. Dollars.)*

$[NUMERIC RANGE 1-99999] U.S. Dollars

{DESIGN: GRID B1_1- B1_9}

B1. *People pursue personal genome sequencing for many different reasons. For each of the following statements listed here and on the next page, please select the response that is most appropriate for you.*

How important were the following factors in your decision to pursue personal genome sequencing through {DISPLAY PRE_1}?

1 Not at all important
2 Somewhat important
3 Very important
4 Not applicable

B1_1. Curiosity about my genetic make-up
B1_2. Interest in finding out about my personal disease risk
B1_3. Interest in finding out what I can do to improve my health
B1_4. {PRG: Do not show if PRE_1=4 OR 5} Interest in finding out about my personal response to medications
B1_5. Desire to plan for the future
B1_6. General interest in genetics
B1_7. It seemed like it would be fun and entertaining
B1_19. Desire to participate in research to help others
B1_8. {PRG: Show if PRE_1=2 OR 4} Desire to contribute my genome sequence data to research
B1_9. {PRG: Show if PRE_1=2} Desire to learn about my genetic make-up without going through a physician
B1_18. {PRG: Do not show if PRE_1=4 OR 5} Interest in my ancestry

---

{DESIGN: GRID B1_10- B1_17}
{DESIGN: B1 & B1_OTH ON SAME PAGE}

B1. *People pursue personal genome sequencing for many different reasons. For each of the following statements, please select the response that is most appropriate for you.*

How important were the following factors in your decision to pursue personal genome sequencing through {DISPLAY PRE_1}?

1 Not at all important
2 Somewhat important
3 Very important
4 Not applicable

B1_10. Other members of my family have had their genomes sequenced
B1_11. To learn more about my genetics because I lack information about my family history
B1_12. To provide disease risk information for my children
B1_13. There is a medical condition in my family that may be genetic
B1_14. There is a medical condition in my family that has been confirmed to be genetic
B1_15. {PRG: Show if K1=1} To learn more about my genetics because I am adopted
B1_16. To learn more about genome sequencing as part of my professional activities
B1_17. To contribute to the advancement of science
B1_20. Interest in learning as much about myself as possible
B1_21. It seemed like a novel opportunity

B1_OTH. If any other factors were important in your decision of whether or not to pursue personal genome sequencing through {DISPLAY PRE_1}, please specify here.

[TEXT RESPONSE] {PRG: 300 CHARACTER LIMIT}

{PRG: HIDE ANY OPTION TO WHICH THE CORRESPONDING B1_ ROW = 1 or 4}

B1_MOST. Which factor was the **MOST** important in your decision to pursue personal genome sequencing through {DISPLAY PRE_1}?

1 Curiosity about my genetic make-up
2 Interest in finding out about my personal disease risk
3 Interest in finding out what I can do to improve my health
4 Interest in finding out about my personal response to medications
5 Desire to plan for the future
6 General interest in genetics
7 It seemed like it would be fun and entertaining
19 Desire to participate in research to help others
8 PRG: Show if PRE_1=2} Desire to contribute my genome sequence data to research
9 {PRG: Show if PRE_1=2} Desire to learn about my genetic make-up without going through a physician
18 Interest in my ancestry
10 Other members of my family have had their genomes sequenced
11 To learn more about my genetics because I lack information about my family health history
12 To provide disease risk information for my children
13 There is a medical condition in my family that may be genetic
14 There is a medical condition in my family that has been confirmed to be genetic
15 {PRG: Show if K1=1} To learn more about my genetics because I am adopted
16 To learn more about genome sequencing as part of my professional activities
17 To contribute to the advancement of science
20 Interest in learning as much about myself as possible
21 It seemed like a novel opportunity
98 [TEXT RESPONSE FROM B1_OTH]

{PRG: SHOW IF B1_14=2 OR 3}

B1_14a. What confirmed genetic condition(s) do you have in your family?

[MEMO RESPONSE]

{DESIGN: GRID B2_1-B2_7}
{DESIGN: SAME SCREEN B2-B2_OTH}

B2. *People have different concerns about personal genome sequencing. For each of the following statements, please select the response that is most appropriate for you.*

How concerned were you about each of the following factors when deciding whether or not to pursue personal genome sequencing through {DISPLAY PRE_1}?

1 Not at all concerned
2 Somewhat concerned
3 Very concerned

B2_1. How well the results would predict my future disease risk
B2_2. The privacy of my genetic information
B2_4. The possibility that I might receive unwanted information
B2_5. The financial cost of having my genome sequenced
B2_6. The complexity of genetic variant interpretation
B2_7. The impact the results might have on my ability to obtain insurance

B2_OTH. If you were concerned about any other factors when deciding whether or not to pursue personal genome sequencing, please specify here.

[OPEN END RESPONSE]

{DESIGN: GRID F7a-F7e}

F7. *Please reflect on the decision that you made to pursue personal genome sequencing.*

To what extent do you agree or disagree with the following statements?

1 Strongly disagree
2 Somewhat disagree
3 Neither agree nor disagree
4 Somewhat agree
5 Strongly agree

F7a. It was the right decision.
F7b. I regret the decision that I made.
F7c. I would make the same decision again.
F7d. The decision did me a lot of harm.
F7e. The decision was a wise one.

F3. In general, how satisfied are you with your decision to obtain personal genome sequencing through {DISPLAY PRE_1}?

1 Not at all satisfied
2 Not very satisfied
3 Somewhat satisfied
4 Very satisfied

{Note: Section Header 3: Your Genome Results Report: Information}

Invitae Participant Note {PRG: DO NOT SHOW}
{PRG: SHOW ON ALL PAGES IF PRE_1=9 (INVITAE)}
Invitae Participants: In this survey, ***genome*** refers to ***genetic***, ***genome sequencing*** refers to ***genetic testing***, and ***genome reports*** refer to ***genetic test reports***.

TEXT. *Next, we'd like to ask you some questions about your thoughts on the personal genome sequencing results you will receive through {DISPLAY PRE_0}.*

B3. How many health conditions do you expect to learn about through your personal genome sequencing? *(We know you cannot predict how this genetic test will look, but please provide your best estimate.)*

[NUMERICAL RESPONSE 0-200]

B3a. Please specify what types of conditions you expect to learn about.

[OPEN END RESPONSE]

B6. Please answer the following questions about what you expect to learn from your personal genome sequencing results.

I expect my genetic test results to:

1 Strongly Disagree
2 Disagree
3 Neither Agree nor Disagree
4 Agree
5 Strongly Agree

B6_1. Help explain a condition that I have
B6_2. Help explain a family history of disease
B6_3. Reassure me that I am healthy
B6_4. Give me information about specific diseases that I am concerned about
B6_5. Help tailor treatment(s) to me specifically
B6_6. Help me prevent future diseases
B6_7. Help me learn more about the risk of passing on a disease to my children or other descendants

{PRG: SHOW IF B6_4=4 OR 5; OTHERWISE SKIP TO B7}

B6b. Please specify which disease(s) you are concerned about:

[TEXT RESPONSE]

B7. Please indicate how much you agree or disagree with the following statements.

1 Strongly Disagree
2 Disagree
3 Neither Agree Nor Disagree
4 Agree
5 Strongly Agree

B7a. I am concerned about receiving genome sequencing results with uncertain meaning.
B7b. I am concerned that I will not be able to understand my genome sequencing results.
B7c. I am concerned about receiving information that I cannot do anything about.
B7d. I am concerned about my ability to cope with receiving my genome sequencing results.
B7e. I am not at all concerned about receiving my genome sequencing results.

{DESIGN: GRID C0}
{PRG: SHOW C0_8 IF PRE_1=3}

C0. Please indicate whether you ***were offered*** each of the following types of results as part of your personal genome sequencing report (i.e., your {RESPONSE:PRE_1} results).

1 Yes
2 No

C0_1. Information about my risk of a disease or diseases
C0_2. How my body responds to drugs or medications
C0_3. My carrier status
C0_4. Information about my ancestry
C0_5. Information explaining the cause of a disease I already knew I had
C0_6. My *APOE* genotype
C0_8. Physical traits (e.g. bitter tasting type)
C0_7. Other

{PRG: SHOW C0_OTH IF C0.7=1; OTHERWISE SKIP TO C13}

C0_OTH. What other types of results were you offered as part of your personal genome sequencing report?

[TEXT RESPONSE] {300 CHARACTER LIMIT}

{PRG: ASK C13 GRID IF ANY OF C0 ARE SELECTED, AND PRESENT ONLY CORRESPONDING ROWS WHERE C0 = 1.}

C13. Please indicate how interested you are in receiving each type of result as part of your personal genome sequencing report (i.e., your {RESPONSE:PRE_1} results)?

1 Not at all interested
2 Somewhat interested
3 Very interested

C13_1. Information about my risk of a disease or diseases
C13_2. How my body responds to drugs or medications
C13_3. My carrier status
C13_4. Information about my ancestry
C13_5. Information explaining the cause of a disease I already knew I had
C13_6. My *APOE* genotype
C13_8. Physical traits (e.g. bitter tasting type)
C13_7. [TEXT RESPONSE FROM C0_OTH]

A1. Do you plan to discuss your {DISPLAY PRE_1} results with anyone?

1 Yes
0 No

{PRG: SHOW A1_A IF A1 = 0 OTHERWISE GOTO FILTER BEFORE A2}

A1_A. Why do you plan to **NOT** discuss your [DISPLAY PRE_1] results with anyone?

[OPEN ENDED RESPONSE]

{PRG: SHOW IF A1=1; OTHERWISE SKIP TO A3_A}

A2. With whom do you plan to discuss your {DISPLAY PRE_1} results? *(Please select all that apply.)*

1 Family members
2 Friends
3 Co-workers/colleagues
4 Healthcare provider {PRG: IF PRE_1=4 OR 5, DISPLAY "(other than Dr. Caskey)"}
5 Contacts on social networking services (e.g., Facebook, Twitter)
6 Contacts on health- or disease-based social networking services (e.g., {PRG: IF PRE_1=1, DISPLAY "UYG Community,"} PatientsLikeMe, Cure Together, disease-specific patient networks)
7 Other *(Please specify):* [TEXT RESPONSE]

{PRG: SHOW A2_A IF A2 DOES NOT EQUAL 1, OTHERWISE GOTO A2_1A}

A2_A. Why do you **NOT** plan to discuss your [DISPLAY PRE_1] results with family members? *(Please select all that apply.)*

1 I don't feel that my results will be important enough to share with my family members.
2 I don't think family members will be interested in my results.
3 I am concerned about how my family members might react to my results.
5 Other *(Please specify):* [TEXT RESPONSE]

{PRG: SHOW IF A2=1; OTHERWISE SKIP TO A3_A}
{PRG: SELECT ALL THAT APPLY}

A2_1a. With which family member(s) do you plan to talk about your {DISPLAY PRE_1} results? *(Please select all that apply.)*

1 Spouse/significant other/partner
2 Children
3 Brothers or sisters
4 Parents
5 Other relatives *(Please specify):* [TEXT RESPONSE]

{PRG: SHOW IF A2 DOES NOT=4 OR A1=0; OTHERWISE GO TO FILTER BEFORE A3_C}

A3_a. Why do you **NOT** plan to discuss your [DISPLAY PRE_1] results with a healthcare provider {PRG: IF PRE_1=4 OR 5, DISPLAY "(other than Dr. Caskey)"}? *(Please select all that apply.)*

1 I have concerns about my genome sequencing results being placed in my medical record.
2 I don't feel that my results will be important enough to share with a healthcare provider.
4 I am confident in my ability to understand my results without the aid of a healthcare provider.
5 I do not think my healthcare provider is knowledgeable enough about genomic sequencing to use my results in my medical care.
6 I do not think I will receive any results that will require medical follow-up.
7 Other *(Please specify):* [TEXT RESPONSE]

TEXT: *Now we would like to ask you a few questions about various health screens.*

C1. Blood cholesterol is a fatty substance found in the blood. Blood can be taken and used to determine your cholesterol level.

Have you ever had a blood test to check your cholesterol?

1	Yes, within the past year
2	Yes, more than a year ago
0	No
99	I don't know

C2. A blood glucose test is a blood test that measures the amount of sugar in your blood.

Have you ever had your blood sugar tested?

1	Yes, within the past year
2	Yes, more than a year ago
0	No
99	I don't know

C3_a. A colonoscopy/sigmoidoscopy is when a tube is inserted in the rectum to view the bowel for signs of cancer or other health problems. In this exam, all or part of the colon is checked. Anesthesia or pain medication is usually required.

Have you ever had a colonoscopy or sigmoidoscopy?

1	Yes, within the past year
2	Yes, more than a year ago
0	No
99	I don't know

C3_b. A fecal occult blood test (FOBT) or fecal immunochemical test (FIT) are two other tests used to screen for signs of colorectal cancer. For these tests, a stool sample is collected and checked for the presence of blood. There is no invasive testing involved, and no anesthesia or pain medications are required.

Have you ever had a FOBT or FIT test?

1	Yes, within the past year
2	Yes, more than a year ago
0	No
99	I don't know

C4. Have you ever had any tests in which a physician or healthcare provider looked for signs of heart disease, for example, an EKG (electrocardiogram) or a stress test?

1	Yes, within the past year
2	Yes, more than a year ago
0	No
99	I don't know

{PRG: SHOW C5-C7 IF DEM4b=1 OR 3 OR 4; OTHERWISE SKIP TO FILTER BEFORE C8}

C5. A mammogram is an x-ray of each breast to look for early signs of breast cancer.

Have you ever had a mammogram?

1	Yes, within the past year
2	Yes, more than a year ago
0	No
99	I don't know

C5a. MRI scans use magnets and radio waves instead of x-rays to produce very detailed, cross-sectional images of the body. MRI scans can take a long time---often up to an hour. You have to lie inside a narrow tube. For breast imaging, doctors inject a dye into a small vein in the arm before or during the exam.

Have you ever had a breast MRI scan?

1	Yes, within the past year
2	Yes, more than a year ago
0	No
99	I don't know

C6. A clinical breast exam is when a physician, nurse, or other healthcare provider feels your breasts for lumps.

Have you ever had a clinical breast exam?

1	Yes, within the past year
2	Yes, more than a year ago
0	No
99	I don't know

C7. A Pap test, also called a Pap smear, is when a physician or other healthcare provider uses a special stick or brush to take a few cells from inside and around the cervix.

Have you ever had a Pap test?

1	Yes, within the past year
2	Yes, more than a year ago
0	No
99	I don't know

{PRG: SHOW IF DEM4b=2 OR 3 OR 4; OTHERWISE SKIP TO C12}

C8. A Prostate-Specific Antigen test, also called a PSA test, is a blood test used to check men for prostate cancer.

Have you ever had a PSA test?

1	Yes, within the past year
2	Yes, more than a year ago
0	No
99	I don't know

C8a. A digital rectal exam (DRE) is when a physician or other healthcare provider inserts a gloved finger into the rectum to examine the prostate for irregularities in size, shape, or texture.

Have you ever had a DRE?

1 Yes, within the past year
2 Yes, more than a year ago
0 No
99 I don't know

C12. Have you ever had skin cancer (melanoma) screening (e.g., a physical examination of your skin to check for signs of cancer)?

1 Yes, within the past year
2 Yes, more than a year ago
0 No
99 I don't know

C9. Have you ever had any blood tests, imaging studies, or exams in which a physician or healthcare provider looked for signs of cancer, **other than those already listed above**?

1 Yes, within the past year
2 Yes, more than a year ago
0 No
99 I don't know

{PRG: SHOW C9a IF C9 = 1 OR 99 OTHERWISE GO TO C11}

C9a. What other cancer screening tests/exams have you had?

[MEMO RESPONSE]

C11. In total, how many medical visits with a physician or other healthcare provider have you had in the past year? *(If you are unsure, please provide your best estimate.)*

[NUMERIC RANGE 0-50] visits

G4a. Please list any medications (including prescription and non-prescription) and/or supplements you currently are taking.

G4a_1 [TEXT RESPONSE]
G4a_2 [TEXT RESPONSE]
G4a_3 [TEXT RESPONSE]
G4a_4 [TEXT RESPONSE]
G4a_5 [TEXT RESPONSE]
G4a_6 [TEXT RESPONSE]
G4a_7 [TEXT RESPONSE]
G4a_8 [TEXT RESPONSE]
G4a_9 [TEXT RESPONSE]
G4a_10 [TEXT RESPONSE]

{DESIGN: G4b_1 –G4b_10}
{PRG: ONLY DISPLAY ROWS BELOW WHICH WERE ANSWERED IN G4a}

G4b. What type of medication/supplement is this?

1 Prescription medication
2 Non-prescription medication
3 Nutritional supplement
4 Alternative medicine
5 Other

G4b_1. {RESPONSE: G3a_1}
G4b_2. {RESPONSE: G3a_2}
G4b_3. {RESPONSE: G3a_3}
G4b_4. {RESPONSE: G3a_3}
G4b_5. {RESPONSE: G3a_3}
G4b_6. {RESPONSE: G3a_3}
G4b_7. {RESPONSE: G3a_3}
G4b_8. {RESPONSE: G3a_3}
G4b_9. {RESPONSE: G3a_3}
G4b_10. {RESPONSE: G3a_3}

G2. Do you think you will use your {DISPLAY PRE_1} results to guide your **future** use of medications/supplements?

1 Yes
0 No
99 I don't know

{Note: Section Header 4: Your Genome Results Report: Attitudes}

Invitae Participant Note {PRG: DO NOT SHOW}
{PRG: SHOW ON ALL PAGES IF PRE_1=9 (INVITAE)}
Invitae Participants: In this survey, ***genome*** refers to ***genetic***, ***genome sequencing*** refers to ***genetic testing***, and ***genome reports*** refer to ***genetic test reports***.

{DESIGN: GRID F5a-F5e}

F5. To what extent do you agree or disagree with the following statements?

1 Strongly disagree
2 Somewhat disagree
3 Neither agree nor disagree
4 Somewhat agree
5 Strongly agree

F5a. The information I receive about my genome will likely influence how I manage my health in the future.
F5b. Having personal genome sequencing makes me feel like I have more control over my health.
F5d. Having personal genome sequencing will help me to get a better perspective on my health status.
F5e. What I will learn from my personal genome sequencing will help reduce my chances of getting sick.

B4. *Please read the following questions and for each one select the response that best applies to you:*

Do you feel the information you will receive through your personal genome sequencing results with {DISPLAY PRE_1} will…

1 No, definitely not
2 No, probably not
3 Yes, probably
4 Yes, definitely

B4b. …influence what treatment you receive for current or future medical problems?
B4c. …influence decisions you make about your medical care?
B4d. …influence your reproductive decisions?
B4e. …influence what medications you take?
B4f. …influence your end of life planning (e.g., whether you get or change your advanced directive/living will)?
{PRG: SHOW B4g IF K8=7}
B4g. ...influence your professional practice?

{PRG: SHOW IF K3a>0}

INTRO: *The next questions are about some feelings you may have before receiving your personal genome sequencing results.*

PG1. Do you think you may have passed on any genetic diseases or conditions to your children and/or grandchildren?

1 Yes
0 No
2 I don't know

{PRG: SHOW PG9 IF PG1=1, OTHERWISE SKIP TO B5}

PG9. How guilty do you feel about possibly passing on genetic risk to your children or grandchildren?

1 Not at all guilty
2 Not very guilty
3 Somewhat guilty
4 Very guilty
5 Not applicable

{PRG: USE LIKERT-TYPE SCALE FORMAT FOR B5-B6 AND PLACE ON THE SAME PAGE}

B5. On a scale of 1 to 10, how useful do you think your personal genome sequencing results through {DISPLAY PRE_1} will be to you **when you first receive them**?

1 Not at all useful – 10 Extremely useful

B6. On a scale of 1 to 10, how useful do you think your personal genome sequencing results through {DISPLAY PRE_1} will be to you **in the future**?

1 Not at all useful – 10 Extremely useful

{NOTE: SECTION HEADER 5: Your Entire Genome Sequence}

Invitae Participant Note {PRG: DO NOT SHOW}
{PRG: SHOW ON ALL PAGES IF PRE_1=9 (INVITAE)}
Invitae Participants: In this survey, ***genome*** refers to ***genetic***, ***genome sequencing*** refers to ***genetic testing***, and ***genome reports*** refer to ***genetic test reports***.

DS1. In general, how comfortable are you with the idea of sharing your entire genome sequence (not just the report or interpretation)?

1 Very comfortable
2 Somewhat comfortable
3 Somewhat uncomfortable
4 Very uncomfortable

{DESIGN: GRID DS2}

DS2. Would you be willing to share your entire genome sequence…

1 Yes
2 No
3 Not sure

DS2a. …with scientific researchers, as long as your identity remained anonymous?
DS2b. …with scientific researchers, with your identity attached to it?
DS2c. …publicly, as long as your identity remained anonymous?
DS2d. …publicly, with your identity attached to it?

DS3. If someone had access to your entire genome sequence (not just the report or interpretation), and nothing else, how easy or difficult do you think it would be for them to discover your identity?

1 Very easy
2 Quite easy
3 Quite difficult
4 Very difficult
5 Impossible

{Note: Section Header 6: General Attitudes Towards Genomics}

Invitae Participant Note {PRG: DO NOT SHOW}
{PRG: SHOW ON ALL PAGES IF PRE_1=9 (INVITAE)}
Invitae Participants: In this survey, ***genome*** refers to ***genetic***, ***genome sequencing*** refers to ***genetic testing***, and ***genome reports*** refer to ***genetic test reports***.

Intro: *In this section, we are interested in learning about your attitudes and thoughts about genome sequencing in general.*

{PRG: GRID F6e – F6p}

F6. Please indicate whether you agree or disagree with each of the following statements.

1 Strongly disagree
2 Somewhat disagree
3 Neither agree nor disagree
4 Somewhat agree
5 Strongly agree

F6e. Health insurance should cover personal genome sequencing.
F6f. Personal genomic information should be part of a standard medical record.
F6g. Parents should be able to get personal genome sequencing for their children if they want to.
F6n. The government should put more effort into regulating personal genome sequencing.
F6o. Personal genome sequencing should only be available to people through their doctor.
F6p. People have a right to access their own personal genomic information without going through a healthcare provider.

M4. Please indicate whether you agree or disagree with each of the following statements.

1 Strongly disagree
2 Somewhat disagree
3 Neither agree nor disagree
4 Somewhat agree
5 Strongly agree

M4a. Once a variant in a gene that affects a person's risk of a disease is found, that disease can always be prevented or cured.
M4b. A healthcare provider can tell a person their exact chance of developing a disease based on the results from genome sequencing.
M4c. Scientists know how all variants of genes will affect a person's chances of developing diseases.
M4d. Even if a person has a variant in a gene that affects their risk of disease, they may not develop that disease.
M4e. Genome sequencing is a routine test that most people can have through their physician's office.
M4f. Genome sequencing may find variants in a person's genes that they can pass on to their children.
M4g. Genome sequencing may give a person information about their chances of developing several different diseases.
M4h. Genome sequencing may find variants in a person's genes that will increase their chance of developing a disease in their lifetime.
M4i. Genome sequencing may find variants in a person's genes that will decrease their chance of developing a disease in their lifetime.
M4j. Genome sequencing may find variants in a person's genes that may determine how they respond to certain medicines.
M4k. A person's health habits, such as diet and exercise, can affect whether or not their genes cause diseases such as heart disease and cancer.

{Section Header 7: A Few More Questions About You}

Invitae Participant Note {PRG: DO NOT SHOW}
{PRG: SHOW ON ALL PAGES IF PRE_1=9 (INVITAE)}
Invitae Participants: In this survey, ***genome*** refers to ***genetic***, ***genome sequencing*** refers to ***genetic testing***, and ***genome reports*** refer to ***genetic test reports***.

Q3. Have you ever received personal genetic testing from another source (e.g., 23andMe, carrier screening, BRCA1 or BRCA2 testing)?

1 Yes, one other source
3 Yes, multiple sources
0 No
2 I don't know

{PRG: SHOW Q3b and Q3c IF Q3=1 OR 3; OTHERWISE SKIP TO Q3d}

Q3b. Why did you receive personal genetic testing from another source? (*Please select all that apply.)*

1 Because my doctor recommended it
2 Because my partner or I were pregnant/planning to get pregnant and we wanted to know if there were any conditions or diseases we might pass on to our child
3 Because I was concerned about a condition or disease that runs in my family
4 To diagnose a condition or disease that I already have
5 Because I was curious about health and traits predicted by my genetic make-up
6 Because I was curious about my ancestry
7 To tailor medications to my genetic information
8 Other *(Please specify):* [TEXT RESPONSE]

Q3c. What type(s) of personal genetic testing have you previously received? *(Please select all that apply.)*

1 Carrier screening through a physician (e.g., testing if you are a carrier of cystic fibrosis)
2 Prenatal diagnosis through a physician (e.g., amniocentesis)
3 Testing for a specific gene or genetic condition through a physician (e.g., BRCA1 sequencing)
4 Direct-to-consumer ancestry testing (e.g., Family Tree DNA, 23andMe ancestry report)
5 Direct-to-consumer health testing (e.g., 23andMe health report)
6 Other *(Please specify):* [TEXT RESPONSE]

Q3d. Have you received personal genome sequencing from any source other than {DISPLAY PRE_1}?

1 Yes
2 No

{PRG: SHOW Q3e AND Q3f IF Q3d=1, OTHERWISE SKIP TO K12}
{PRG: DISPLAY Q3e AND Q3f ON SAME PAGE}

Q3e. From what other company or institution did you receive personal genome sequencing?

[OPEN RESPONSE]

Q3f. Please summarize any differences between these services.

[OPEN RESPONSE]

K12. Would you say that in general your health is:

1 Excellent
2 Very good
3 Good
4 Fair
5 Poor

{DISPLAY: J1A & J1B SIDE BY SIDE ON THE SCREEN}
{DISPLAY: J1 & J2 ON SAME PAGE}

J1. What is your height?

J1a. [NUMERIC RANGE 4-7] feet J1b. [NUMERIC RESPONSE, 0-11] inches

J2. What is your weight?

[NUMERIC RANGE 70-400] pounds

C10. In what calendar year did you have your last general physical check-up?

[NUMERIC RANGE 1950-2015]

{DESIGN: GRID C3_1 TO C3_14}

C3. Has a healthcare provider ever told you that you have (or had) any of the following medical conditions?

1 Yes
0 No

C3_1. Arthritis
C3_2. Asthma
C3_3. Cancer
C3_4. Chronic kidney disease
C3_5. Diabetes
C3_6. Eye conditions
C3_7. Gastrointestinal (GI) conditions
C3_8. Heart conditions
C3_9. High cholesterol
C3_10. Lupus
C3_11. Mental illness/psychiatric conditions
C3_12. Neurological conditions (e.g., Alzheimer's disease, Multiple sclerosis, Parkinson's disease)
C3_13. Psoriasis
C3_14. Bleeding disorder

{PRG: SHOW C3_1_1-13 IF ANY C3_1-C3_13=1, OTHERWISE SKIP TO FILTER BEFORE C3_1a}

C3_1_1-13. Do you **currently** have any of the following medical conditions?

1 Yes
0 No

{PRG: SHOW C3_1_1 IF C3_1=1}	C3_1_1.	Arthritis
{PRG: SHOW C3_1_2 IF C3_2=1}	C3_1_2.	Asthma
{PRG: SHOW C3_1_3 IF C3_3=1}	C3_1_3.	Cancer
{PRG: SHOW C3_1_4 IF C3_4=1}	C3_1_4.	Chronic kidney disease
{PRG: SHOW C3_1_5 IF C3_5=1}	C3_1_5.	Diabetes
{PRG: SHOW C3_1_6 IF C3_6=1}	C3_1_6.	Eye conditions
{PRG: SHOW C3_1_7 IF C3_7=1}	C3_1_7.	Gastrointestinal (GI) conditions
{PRG: SHOW C3_1_8 IF C3_8=1}	C3_1_8.	Heart conditions
{PRG: SHOW C3_1_9 IF C3_9=1}	C3_1_9.	High cholesterol
{PRG: SHOW C3_1_10 IF C3_10=1}	C3_1_10.	Lupus
{PRG: SHOW C3_1_11 IF C3_11=1}	C3_1_11.	Mental illness/psychiatric conditions
{PRG: SHOW C3_1_12 IF C3_12=1}	C3_1_12.	Neurological conditions (e.g., Alzheimer's disease, ALS, Multiple sclerosis, Parkinson's disease)
{PRG: SHOW C3_1_13 IF C3_13=1}	C3_1_13.	Psoriasis
{PRG: SHOW C3_1_14 IF C3_14=1}	C3_1_14.	Bleeding disorder

{PRG: SHOW C3_1a IF C3_1=1; OTHERWISE SKIP TO FILTER BEFORE C3_3a}

C3_1a. Which of the following types of **arthritis** has a healthcare provider told you that you have/had? (*Please select all that apply.)*

1 Osteoarthritis ("wear and tear" on joints)
2 Rheumatoid arthritis (joint swelling and stiffness)
3 Other *(Please specify):* [TEXT RESPONSE]

{PRG: SHOW C3_3a IF C3_3=1; OTHERWISE SKIP TO FILTER BEFORE C3_4a}

C3_3a. Which of the following types of **cancer** has a healthcare provider told you that you have/had? (*Please select all that apply.)*

{PRG: SHOW C3_3a.1 IF DEM4B=1 | 3 | 4}
1 Breast cancer
2 Colorectal cancer
3 Esophageal cancer
4 Leukemia
5 Lung cancer
{PRG: SHOW C3_3a.6 IF DEM4B=2 | 3 | 4}
6 Prostate cancer
7 Skin cancer (Melanoma)
8 Stomach cancer
9 Other *(Please specify):* [TEXT RESPONSE]

{PRG: SHOW C3_5a IF C3_5=1; OTHERWISE SKIP TO FILTER BEFORE C3_6a}

C3_5a. Which of the following types of **diabetes** has a healthcare provider told you that you have/had? (*Please select all that apply.)*

1 Type 1 (insulin dependent, juvenile onset)
2 Type 2 (non-insulin dependent)
3 I don't remember

{PRG: SHOW C3_6a IF C3_6=1; OTHERWISE SKIP TO FILTER BEFORE C3_7a}

C3_6a. Which of the following **eye conditions** has a healthcare provider told you that you have/had? (*Please select all that apply.)*

1 Age-related macular degeneration
2 Glaucoma
3 Cataracts
4 Other *(Please specify):* [TEXT RESPONSE]

{PRG: SHOW C3_7a IF C3_7=1; OTHERWISE SKIP TO FILTER BEFORE C3_8a}

C3_7a. Which of the following **gastrointestinal (GI) conditions** has a healthcare provider told you that you have/had? (*Please select all that apply.)*

1 Celiac disease
2 Crohn's disease
3 Ulcerative colitis
4 Other *(Please specify):* [TEXT RESPONSE]

{PRG: SHOW C3_8a IF C3_8=1; OTHERWISE SKIP TO FILTER BEFORE C3_7a}

C3_8a. Which of the following **heart conditions** has a healthcare provider told you that you have/had? (*Please select all that apply.)*

1 Irregular heartbeat (Atrial fibrillation)
2 Coronary artery disease
3 Peripheral arterial disease
4 Blood clotting (Venous thromboembolism)
5 Other *(Please specify):* [TEXT RESPONSE]

{PRG: SHOW C3_11a IF C3_11=1; OTHERWISE SKIP TO FILTER BEFORE C3_12a}

C3_11a. Which of the following **mental illness/psychiatric conditions** has a healthcare provider told you that you have/had? *(Please select all that apply.)*

3 Anxiety disorder
1 Bipolar disorder
2 Depression
4 Other *(Please specify):* [TEXT RESPONSE]

{PRG: SHOW C3_12a IF C3_12=1; OTHERWISE SKIP TO C4}

C3_12a. Which of the following **neurological conditions** has a healthcare provider told you that you have/had? (*Please select all that apply.)*

1 Alzheimer's disease
2 ALS (Lou Gehrig's disease)
3 Multiple sclerosis
4 Parkinson's disease
5 Other *(Please specify):* [TEXT RESPONSE]

{PRG: SHOW C3_14a IF C3_14=1; OTHERWISE SKIP TO C4}

C3_14a. Which of the following bleeding disorders has a healthcare provider told you that you have/had? *(Select all that apply.)*

1 Von Willebrand Disease
2 Hemophilia A (Factor VIII Deficiency)
3 Hemophilia B (Congenital Factor IX Deficiency)
4 Congenital Fibrinogen (Factor I) Deficiency
5 Congenital Prothrombin (Factor II) Deficiency
6 Congenital Factor VII Deficiency
7 Congenital Factor X Deficiency
8 Congenital Factor XIII Deficiency
9 Other (Please specify): [TEXT RESPONSE]

{DESIGN: GRID C4_1-C4_14}

C4. Have any of your blood relatives (a parent, brother or sister, child, grandparent, aunt, uncle, or first cousin) ever had any of the following conditions?

1 Yes
0 No

C4_1 Arthritis
C4_2 Asthma
C4_3 Cancer
C4_4 Chronic kidney disease
C4_5 Diabetes
C4_6 Eye conditions
C4_7 Gastrointestinal (GI) conditions
C4_8 Heart conditions
C4_9 High cholesterol
C4_10 Lupus
C4_11 Mental illness/psychiatric conditions
C4_12 Neurological conditions (e.g., Alzheimer's disease, ALS, Multiple sclerosis, Parkinson's disease)
C4_13 Psoriasis
C4_14 Bleeding disorder

{PRG: SHOW C5 GRID IF ANY OF C4_1 - C4_14 = 1, OTHERWISE SKIP TO E1}
{DESIGN: GRID C5_1-C5_15}
{PRG: SELECT ALL THAT APPLY}

C5. Which of your blood relatives (a parent, brother or sister, child, grandparent, aunt, uncle, or first cousin) have ever had any of the following conditions? (*Please select all that apply.*)

1 A parent
2 A brother or sister
3 A child
4 A grandparent
5 An aunt, uncle, or first cousin

{PRG: SHOW C5_1 IF C4_1 = 1}	C5_1.	Arthritis
{PRG: SHOW C5_2 IF C4_2 = 1}	C5_2.	Asthma
{PRG: SHOW C5_3 IF C4_3 = 1}	C5_3.	Cancer
{PRG: SHOW C5_4 IF C4_4 = 1}	C5_4.	Chronic kidney disease
{PRG: SHOW C5_5 IF C4_5 = 1}	C5_5.	Diabetes
{PRG: SHOW C5_6 IF C4_6 = 1}	C5_6.	Eye conditions
{PRG: SHOW C5_7 IF C4_7 = 1}	C5_7.	Gastrointestinal (GI) conditions
{PRG: SHOW C5_8 IF C4_8 = 1}	C5_8.	Heart conditions
{PRG: SHOW C5_9 IF C4_9 = 1}	C5_9.	High cholesterol
{PRG: SHOW C5_10 IF C4_10 = 1}	C5_10.	Lupus
{PRG: SHOW C5_11 IF C4_11 = 1}	C5_11.	Mental illness/psychiatric conditions
{PRG: SHOW C5_12 IF C4_12 = 1}	C5_12.	Neurological conditions (e.g., Alzheimer's disease, ALS, Multiple sclerosis, Parkinson's disease)
{PRG: SHOW C5_13 IF C4_13 = 1}	C5_13.	Psoriasis
{PRG: SHOW C5_14 IF C4_14 = 1}	C5_14.	Bleeding disorder

{NOTE: DK SHOULD NOT BE MUTUALLY EXCLUSIVE IN FOLLOWING GRIDS}

{PRG: SHOW C5_1a_1 - C5_1a_5 GRID IF C5_1= 1-5, OTHERWISE SKIP TO FILTER BEFORE C5_3a_1- C5_3a_5 GRID}

{DESIGN: GRID C5_1a_1 - C5_1a_5}
{PRG: C5_1a_1 - C5_1a_5 SELECT ALL THAT APPLY}

Please select the type(s) of {PRG: IF PRE_1=1 DISPLAY "**arthritis**"; OTHERWISE DISPLAY "**arthritis/immune disorders**"} that each of the following relatives has/had. *(Please select all that apply.)*

1 Osteoarthritis ("wear and tear" on joints)
2 Rheumatoid arthritis (joint swelling and stiffness)
3 Other
99 I don't know

{PRG: SHOW C5_1a_1 IF C5_1=1}	C5_1a_1. A parent
{PRG: SHOW C5_1a_2 IF C5_1=2}	C5_1a_2. A brother or sister
{PRG: SHOW C5_1a_3 IF C5_1=3}	C5_1a_3. A child
{PRG: SHOW C5_1a_4 IF C5_1=4}	C5_1a_4. A grandparent
{PRG: SHOW C5_1a_5 IF C5_1=5}	C5_1a_5. An aunt, uncle, or first cousin

---

{PRG: SHOW C5_3a_1 - C5_3a_5 GRID IF C5_3= 1-5, OTHERWISE SKIP TO FILTER BEFORE C5_5a_1- C5_5a_5 GRID}

{DESIGN: GRID C5_3a_1 - C5_3a_5}
{PRG: C5_3a_1 - C5_3a_5 SELECT ALL THAT APPLY}

Please select the type(s) of **cancer** that each of the following relatives has/had. *(Please select all that apply.)*

1	Breast cancer
2	Colorectal cancer
3	Esophageal cancer
4	Leukemia
5	Lung cancer
6	Prostate cancer
7	Skin cancer (Melanoma)
8	Stomach cancer
9	Other
99	I don't know

{PRG: SHOW C5_3a_1 IF C5_3=1}	C5_3a_1. A parent
{PRG: SHOW C5_3a_2 IF C5_3=2}	C5_3a_2. A brother or sister
{PRG: SHOW C5_3a_3 IF C5_3=3}	C5_3a_3. A child
{PRG: SHOW C5_3a_4 IF C5_3=4}	C5_3a_4. A grandparent
{PRG: SHOW C5_3a_5 IF C5_3=5}	C5_3a_5. An aunt, uncle, or first cousin

---

{PRG: SHOW C5_5a_1 - C5_5a_5 GRID IF C5_5 = 1-5, OTHERWISE SKIP TO FILTER BEFORE C5_6a_1- C5_6a_5 GRID}

{DESIGN: GRID C5_5a_1 - C5_5a_5}
{PRG: C5_5a_1 - C5_5a_5 SELECT ALL THAT APPLY}

Please select the type(s) of **diabetes** that each of the following relatives has/had. *(Please select all that apply.)*

1	Type 1 (insulin dependent, juvenile onset)
2	Type 2 (non-insulin dependent)
99	I don't know

{PRG: SHOW C5_5a_1 IF C5_5=1}	C5_5a_1. A parent
{PRG: SHOW C5_5a_2 IF C5_5=2}	C5_5a_2. A brother or sister
{PRG: SHOW C5_5a_3 IF C5_5=3}	C5_5a_3. A child
{PRG: SHOW C5_5a_4 IF C5_5=4}	C5_5a_4. A grandparent
{PRG: SHOW C5_5a_5 IF C5_5=5}	C5_5a_5. An aunt, uncle, or first cousin

---

{PRG: SHOW C5_6a_1 - C5_6a_5 GRID IF C5_6= 1-5, OTHERWISE SKIP TO FILTER BEFORE C5_7a_1- C5_7a_5 GRID}

{DESIGN: GRID C5_6a_1 - C5_6a_5}
{PRG: C5_6a_1 - C5_6a_5 SELECT ALL THAT APPLY}

Please select the type(s) of **eye conditions** that each of the following relatives has/had. *(Please select all that apply.)*

1 Age-related macular degeneration
2 Glaucoma
3 Cataracts
4 Other
99 I don't know

{PRG: SHOW C5_6a_1 IF C5_6=1}	C5_6a_1. A parent
{PRG: SHOW C5_6a_2 IF C5_6=2}	C5_6a_2. A brother or sister
{PRG: SHOW C5_6a_3 IF C5_6=3}	C5_6a_3. A child
{PRG: SHOW C5_6a_4 IF C5_6=4}	C5_6a_4. A grandparent
{PRG: SHOW C5_6a_5 IF C5_6=5}	C5_6a_5. An aunt, uncle, or first cousin

---

{PRG: SHOW C5_7a_1 - C5_7a_5 GRID IF C5_7 = 1-5, OTHERWISE SKIP TO FILTER BEFORE C5_8a_1- C5_8a_5 GRID}

{DESIGN: GRID C5_7a_1 - C5_7a_5}
{PRG: C5_7a_1 - C5_7a_5 SELECT ALL THAT APPLY}

Please select the type(s) of **gastrointestinal (GI) conditions** that each of the following relatives has/had. *(Please select all that apply.)*

1 Celiac disease
2 Crohn's disease
3 Ulcerative colitis
4 Other
99 I don't know

{PRG: SHOW C5_7a_1 IF C5_7=1}	C5_7a_1. A parent
{PRG: SHOW C5_7a_2 IF C5_7=2}	C5_7a_2. A brother or sister
{PRG: SHOW C5_7a_3 IF C5_7=3}	C5_7a_3. A child
{PRG: SHOW C5_7a_4 IF C5_7=4}	C5_7a_4. A grandparent
{PRG: SHOW C5_7a_5 IF C7_4=5}	C5_7a_5. An aunt, uncle, or first cousin

---

{PRG: SHOW C5_8a_1 - C5_8a_5 GRID IF C5_8 = 1-5, OTHERWISE SKIP TO FILTER BEFORE C5_11a_1- C5_11a_5 GRID}

{DESIGN: GRID C5_8a_1 - C5_8a_5}
{PRG: C5_8a_1 - C5_8a_5 SELECT ALL THAT APPLY}

Please select the type(s) of **heart conditions** that each of the following relatives has/had. *(Please select all that apply.)*

1 Irregular heartbeat (Atrial fibrillation)
2 Coronary artery disease
3 Peripheral arterial disease
4 Blood clotting (Venous thromboembolism)
5 Other
99 I don't know

{PRG: SHOW C5_8a_1 IF C5_8=1} C5_8a_1. A parent
{PRG: SHOW C5_8a_2 IF C5_8=2} C5_8a_2. A brother or sister
{PRG: SHOW C5_8a_3 IF C5_8=3} C5_8a_3. A child
{PRG: SHOW C5_8a_4 IF C5_8=4} C5_8a_4. A grandparent
{PRG: SHOW C5_8a_5 IF C5_8=5} C5_8a_5. An aunt, uncle, or first cousin

---

{PRG: SHOW C5_11a_1 - C5_11a_5 GRID IF C5_11= 1-5, OTHERWISE SKIP TO FILTER BEFORE C5_12a_1 - C5_12a_5 GRID}

{DESIGN: GRID C5_11a_1 - C5_11a_5}
{PRG: C5_11a_1 - C5_11a_5 SELECT ALL THAT APPLY}

Please select the type(s) of **mental illness/psychiatric conditions** that each of the following relatives has/had. *(Please select all that apply.)*

3 Anxiety disorder
1 Bipolar disorder
2 Depression
4 Other
99 I don't know

{PRG: SHOW C5_11a_1 IF C5_11=1} C5_11a_1. A parent
{PRG: SHOW C5_11a_2 IF C5_11=2} C5_11a_2. A brother or sister
{PRG: SHOW C5_11a_3 IF C5_11=3} C5_11a_3. A child
{PRG: SHOW C5_11a_4 IF C5_11=4} C5_11a_4. A grandparent
{PRG: SHOW C5_11a_5 IF C5_11=5} C5_11a_5. An aunt, uncle, or first cousin

---

{PRG: SHOW C5_12a_1 - C5_12a_5 GRID IF C5_12= 1-5, OTHERWISE SKIP TO E1}

{DESIGN: GRID C5_12a_1 - C5_12a_5}
{PRG: C5_12a_1 - C5_12a_5 SELECT ALL THAT APPLY}

Please select the type(s) of **neurological conditions** that each of the following relatives has/had. *(Please select all that apply.)*

1 Alzheimer's disease
2 ALS (Lou Gehrig's disease)
3 Multiple sclerosis
4 Parkinson's disease
5 Other
99 I don't know

{PRG: SHOW C5_12a_1 IF C5_12=1} C5_12a_1. A parent
{PRG: SHOW C5_12a_2 IF C5_12=2} C5_12a_2. A brother or sister
{PRG: SHOW C5_12a_3 IF C5_12=3} C5_12a_3. A child
{PRG: SHOW C5_12a_4 IF C5_12=4} C5_12a_4. A grandparent
{PRG: SHOW C5_12a_5 IF C5_12=5} C5_12a_5. An aunt, uncle, or first cousin

---

{PRG: SHOW C5_14a_1 - C5_14a_5 GRID IF C5_14= 1-5, OTHERWISE SKIP TO E1}

{DESIGN: GRID C5_14a_1 - C5_14a_5}
{PRG: C5_14a_1 - C5_14a_5 SELECT ALL THAT APPLY}

Please select the type(s) of bleeding disorders that each of the following relatives has/had. *(Please select all that apply.)*

1 Von Willebrand Disease
2 Hemophilia A (Factor VIII Deficiency)
3 Hemophilia B (Congenital Factor IX Deficiency)
4 Congenital Fibrinogen (Factor I) Deficiency
5 Congenital Prothrombin (Factor II) Deficiency
6 Congenital Factor VII Deficiency
7 Congenital Factor X Deficiency
8 Congenital Factor XIII Deficiency
9 Other
99 I don't know

{PRG: SHOW C5_14a_1 IF C5_14=1} C5_14a_1. A parent
{PRG: SHOW C5_14a_2 IF C5_14=2} C5_14a_2. A brother or sister
{PRG: SHOW C5_12a_3 IF C5_14=3} C5_14a_3. A child
{PRG: SHOW C5_14a_4 IF C5_14=4} C5_14a_4. A grandparent
{PRG: SHOW C5_14a_5 IF C5_14=5} C5_14a_5. An aunt, uncle, or first cousin

---

{DESIGN: GRID E1_1-E_12}

E1. Compared to the average person of your age, what would you say your chances are of developing these conditions?

1 Much lower than average
2 Lower than average
3 Average
4 Higher than average
5 Much higher than average
6 I already have this condition

E1_1. Alzheimer's disease
{PRG: SHOW E1_2 IF DEM4b=1 OR 3 OR 4}
E1_2. Breast cancer
{PRG: SHOWE1_3 IF DEM4b=2 OR 3 OR 4}
E1_3. Prostate cancer
E1_4. Colorectal cancer
E1_5. Lung cancer
E1_6. Diabetes
E1_7. Heart disease (Coronary artery disease)
E1_8. Parkinson's disease

{DESIGN: GRID D1a-D1f}

Now we are going to ask you some questions about yourself and how you have been feeling.

D1. *Please read the following statements and for each one select the response that best applies to you.*

Over the **past two weeks**, how often have you:

1 Not at all
2 Several days
3 More than half of the days
4 Nearly every day

D1a. Felt nervous, anxious, or on edge?
D1b. Been unable to stop or control worrying?
D1c. Felt calm and peaceful?
D1d. Been a happy person?
D1e. Had little interest or pleasure in doing things?
D1f. Felt down, depressed, or hopeless?

E2. You will find below a series of statements that describe how people may react to the uncertainties of life. Please use the scale below to describe to what extent each item is characteristic of you.

1 Not at all characteristic of me 1
2 2
3 3
4 4
5 Entirely characteristic of me 5

E2_1. Unforeseen events upset me greatly.
E2_2. It frustrates me not having all the information I need.
E2_3. Uncertainty keeps me from living a full life.
E2_4. One should always look ahead so as to avoid surprises.
E2_5. A small, unforeseen event can spoil everything, even with the best planning.
E2_6. When it's time to act, uncertainty paralyses me.
E2_7. When I am uncertain, I can't function very well.
E2_8. I always want to know what the future has in store for me.
E2_9. I can't stand being taken by surprise.
E2_10. The smallest doubt can stop me from acting.
E2_11. I should be able to organize everything in advance.
E2_12. I must get away from all uncertain situations.

K13. Please select the statement that best describes the role you prefer to take in dealing with your health care:

1 I prefer to make the final decision about which treatment I will receive.
2 I prefer to make the final decision about my treatment after seriously considering my doctor's opinion.
3 I prefer that my doctor and I share responsibility for deciding which treatment is best for me.
4 I prefer that my doctor make the final decision about which treatment I will receive, but seriously consider my opinion.
5 I prefer to leave all decisions regarding my treatment to my doctor.

{PRG NOTE: SECTION HEADER 8: Wrap-Up}

Invitae Participant Note {PRG: DO NOT SHOW}
{PRG: SHOW ON ALL PAGES IF PRE_1=9 (INVITAE)}
Invitae Participants: In this survey, ***genome*** refers to ***genetic***, ***genome sequencing*** refers to ***genetic testing***, and ***genome reports*** refer to ***genetic test reports***.

You are almost done with the survey! Here is the final set of questions.

S2D. As part of this study, you will be invited to a follow-up survey a few months after receiving your personal genome sequencing results. Your will also be invited to participate in annual follow-up surveys.

May we also contact you about opportunities to participate in future research related to the Personal Genome Sequencing Outcomes Study (e.g., other surveys or interviews)?

1 Yes
0 No

S2E. May we contact you about opportunities to speak with the media about your experience of participating in the Personal Genome Sequencing Outcomes Study?

1 Yes
0 No

{PRG: S2G IS EXEMPT FROM SOFT PROMPT}

S2GA. Is there anything else you would like to share about your experience of participating in {RESPONSE:PRE_0}?

[OPEN ENDED RESPONSE]

S2G. Is there anything else you would like to share about your experience of participating in the Personal Genome Sequencing Outcomes Study thus far?

[OPEN ENDED RESPONSE]

{PRG: SUBMIT SURVEY B AS COMPLETE AND PASS TO SURVEY C}

{PRG: SOFT PROMPT ON DEM6 SCREEN – PROMPT IS SET TO TRIGGER IF DEM6A,C,D,F OR BOTH DEM7A & B ARE BLANK}

DEM6. We hope to complete a follow-up survey with you after you receive your personal genome sequencing results. To ensure that we can locate you then, we would like to capture your mailing address, so that we may send you a letter invitation.

Please provide us with your mailing address:

DEM6a. Street 1:	[TEXT RESPONSE]
DEM6b. Street 2:	[TEXT RESPONSE]
DEM6c. City:	[TEXT RESPONSE]
DEM6d. State/Province:	[TEXT RESPONSE]
DEM6f. Zip/Postal Code:	[TEXT RESPONSE] {PRG: LIMIT TO 10 CHARACTERS}
DEM6e. Country:	[TEXT RESPONSE]
DEM7a. Cell Phone:	[TEXT RESPONSE]
DEM7b. Home or Office Phone:	[TEXT RESPONSE]

DEM6g. Other/International Address Information: [OPEN END RESPONSE]

We have recorded your email as {INSERT DEM2}. Please provide us with an alternative (i.e., home, school, work) that we may use in case we are not able to reach you at your primary email.

DEM7c. Alternative Email: [EMAIL ADDRESS]

PeopleSeq Consortium
Post-disclosure Survey

PEOPLESEQ POST-DISCLOSURE QUESTIONNAIRE SPECIFICATIONS
MASTER

Short URL to direct towards production survey	https://www.ssgresearch.com/peopleseq
Support email address to include in header	peopleseq@ssgresearch.com
Support phone number to include in header (if needed)	

Logo to use if other than SSG logo
Please list network location of other logo to use: GDrive/S15002 PeopleSeq 2015/DEFLOG.gif

---

Mandatoriness (check the appropriate setting)

X All questions are optional unless otherwise noted
All questions are optional with a soft prompt included if no answer is provided
[OTHER SPECIFY FIELDS SHOULD ALSO HAVE SOFT PROMPTS]
All questions are mandatory

Please provide text to use for Mandatoriness prompt if being used (Default text to use is provided below):

GENERAL SOFT PROMPT:
We noticed that you did not answer a question on the previous page. It is important to us that we get a complete set of responses from you. Please return to the previous page by clicking "Previous" and select an answer for each question. If you would rather not select an answer, you may instead continue to the next page by clicking "Next."

OTHER SPECIFY SOFT PROMPT:

You selected 'Other' but did not specify your answer. Please return to the last question by clicking "Previous" and type in your specific answer. If you would rather not specify an answer, you may instead continue to the next page by clicking "Next."

Section Headers

Section Label	Section Start Page #
1. **About You**	4
2. **Your Decision**	5
3. **Your Genome Results Report: Information**	7
4. **Your Genome Results Report: Attitudes**	27
5. **Your Entire Genome Sequence**	31
6. **General Attitudes Towards Genomics**	33
7. **A Few More Questions About You**	34
8. **Wrap-Up**	42

Survey Title to Appear in Header (appears above the section header bar)

The Personal Genome Sequencing Outcomes Study

Welcome Page text (please modify the following as needed)

Welcome to the Personal Genome Sequencing Outcomes Study!

This is one of the first large-scale systematic studies to examine the experiences, attitudes, and outcomes of healthy adults who have chosen to pursue personal genome sequencing. Our hope is that the knowledge gained through this study will play an integral role in shaping the development of genome sequencing policy and its integration into health care.

{PRG: IF AUTO-LOGIN PROTOCOL IS BEING USED, INSERT THE FOLLOWING TEXT}
Please click Start Survey to begin!

Resume Page Text (please modify the following as needed)

Thank you for returning to the survey. Please click “resume” to begin where you last left off…

Terminate Text (please modify the following as needed)

Thank you for considering participation in this study. Participation requires that you provide consent. We will not contact you again about this study. If you believe you received this message in error, please contact us at the email in the upper right corner of the screen so that we may reset your case.

End Page Text

Thank you for your participation! We will email you with a link to the next survey in a year’s time.

For information about the Personal Genome Sequencing Outcomes Study, including contact details, please refer to the study website at: http://www.genomes2people.org/the---peopleseq---study/ {PRG: SET LINK TO OPEN IN NEW WINDOW}

Results of the Personal Genome Sequencing Outcomes Study will be posted on the study website as they become available.

We really appreciate the time you have taken to complete this survey. You will now be entered into a sweepstakes drawing for an Apple Watch upon completion of this study, and we will be in touch to let you know if you are the winner.

You may now close your browser.

Survey Title to Appear in Header (appears above the section header bar)

Personal Genome Sequencing Outcomes Study

GENERAL PROGRAMMING NOTES

For confidentiality purposes, this questionnaire will be programmed as three separate surveys. Survey A will be the consent form, some basic contact information, and a few demographics. Survey B will be the main body of the questionnaire. And Survey C will be asking for additional contact information.

Please pass all demographics collected in Survey A into Survey B and store them in a calculation there – to facilitate data processing/review. Do NOT pass identifiable data, except for DATSTAT_ALTPID.

All emphasis should be programmed in black, all caps text instead of lowercase blue text.

Lines indicate page breaks throughout the survey and show which should questions appear together.

PRELOADS

PRE_1 Genome Sequencing Program

1 UYG
2 PGP
3 HealthSeq
4 CEO Genome Project
5 MD/PhD Genome Project
6 SOAPS
7 Pioneer 100
8. UVM UYG
9. Illumina
10 Hudson Alpha

{NOTE: PRE_0 IS A CALCULATION DRIVEN OFF OF PRE_1 - **IT SHOULD NOT BE PRELOADED}**
PRE_0 Genome Sequencing Program (with Acronym)

1 Understand Your Genome (UYG)
2 The Harvard Personal Genome Project (PGP)
3 the Whole Genome Sequencing in the Clinical Setting (HealthSeq) Study at Mount Sinai
4 CEO Genome Project
5 MD/PhD Genome Project
6 UNLV Survey of Opinions about Personalized-Medicine Services (SOAPS)
7 Pioneer 100
8. UVM UYG
9. Illumina
10 Hudson Alpha

{Note: Section Header 1: About You}

Invitae Participant Note {PRG: DO NOT SHOW}
{PRG: SHOW ON ALL PAGES IF PRE_1=9 (INVITAE)}
Invitae Participants: In this survey, ***genome*** refers to ***genetic***, ***genome sequencing*** refers to ***genetic testing***, and ***genome reports*** refer to ***genetic test reports***.

SECTION INTRO: *First, we would like to ask a few questions about you.*

{PRG: OPTION 10 IS MUTUALLY EXCLUSIVE}

H1. Do you have health insurance or a health coverage plan? *(Please select all that apply.)*

1 Yes, through my employer
2 Yes, through someone else's employer
3 Yes, a plan that I or someone else buys
4 Yes, through Medicare
5 Yes, through Medicaid or Medical Assistance
6 Yes, through the military, CHAMPUS, or the VA
7 Yes, through the Indian Health Service or the Alaska Native Health Service
8 Yes, through some other source *(Please specify):* [TEXT RESPONSE]
9 No, I don't have any coverage
10 I don't know

H2-H4. Do you have…

1 Yes
0 No
99 I don't know

H2. Life insurance?
H3. Disability insurance?
H4. Long-term care insurance?

{PRG: SHOW H3a IF H3=1}

H3a. Is your disability insurance short term, long term, or both?

1 Short term
2 Long term
3 Both short and long term
99 I don't know

L1. Which of the following best applies to you? *(Please note cigarettes refer to tobacco and not electronic cigarettes.)*

1 I smoke cigarettes (including hand-rolled) every day.
2 I smoke cigarettes (including hand-rolled), but not every day.
3 I do not smoke cigarettes at all, but I do smoke tobacco of some kind (e.g., pipe or cigar).
4 I have stopped smoking completely in the last year.
5 I stopped smoking completely more than a year ago.
6 I have never been a smoker (i.e., smoked for a year or more).

{PRG: SHOW IF L1=1 OR 2, ELSE GOTO Q1}
L2. Are you seriously thinking of quitting smoking?

1 Yes, within the next 30 days
2 Yes, within the next 6 months
3 No, not thinking of quitting

{Note: Section Header 2: Your Decision}

SECTION INTRO: *In this section, we will be asking you some questions about the personal genome sequencing that you had done through {DISPLAY PRE_0}.*

Invitae Intro Note {PRG: DO NOT SHOW}
{PRG: SHOW ONLY IF PRE_1=9 (INVITAE)}

NOTE TO INVITAE PARTICIPANTS:

Some terminology used in the survey questions may differ from terms you and your health care professional have used when discussing your Invitae genetic testing and/or your test results. A note is provided at the top of each page of the survey that describes how different terms should be defined; please refer to this note if any terms in the questions are unfamiliar to you.

{PRG: SHOW IF B1_14=2 OR 3}

B1_14a. What confirmed genetic condition(s) do you have in your family?

[MEMO RESPONSE]

{DESIGN: GRID B2_1-B2_7}
{DESIGN: SAME SCREEN B2-B2_OTH}

B2. *People have different concerns about personal genome sequencing. For each of the following statements, please select the response that is most appropriate for you.*

How concerned were you about each of the following factors when deciding whether or not to pursue personal genome sequencing through {DISPLAY PRE_1}?

1 Not at all concerned
2 Somewhat concerned
3 Very concerned

B2_1. How well the results would predict my future disease risk
B2_2. The privacy of my genetic information
B2_4. The possibility that I might receive unwanted information
B2_5. The financial cost of having my genome sequenced
B2_6. The complexity of genetic variant interpretation
B2_7. The impact the results might have on my ability to obtain insurance

B2_OTH. If you were concerned about any other factors when deciding whether or not to pursue personal genome sequencing, please specify here.

[OPEN END RESPONSE]

{DESIGN: GRID F7a-F7e}

F7. *Please reflect on the decision that you made to pursue personal genome sequencing.*

To what extent do you agree or disagree with the following statements?

1 Strongly disagree
2 Somewhat disagree
3 Neither agree nor disagree
4 Somewhat agree
5 Strongly agree

F7a. It was the right decision.
F7b. I regret the decision that I made.
F7c. I would make the same decision again.
F7d. The decision did me a lot of harm.
F7e. The decision was a wise one.

F3. In general, how satisfied are you with your decision to obtain personal genome sequencing through {DISPLAY PRE_1}?

1 Not at all satisfied
2 Not very satisfied
3 Somewhat satisfied
4 Very satisfied

{Note: Section Header 3: Your Genome Results Report: Information}

Invitae Participant Note {PRG: DO NOT SHOW}
{PRG: SHOW ON ALL PAGES IF PRE_1=9 (INVITAE)}
Invitae Participants: In this survey, ***genome*** refers to ***genetic***, ***genome sequencing*** refers to ***genetic testing***, and ***genome reports*** refer to ***genetic test reports***.

TEXT. *Next, we'd like to ask you some questions about the personal genome sequencing results you received through {DISPLAY PRE_0}.*

F1. How many times have you viewed your {DISPLAY PRE_1} results since receiving them?

0 Never
1 1 time
2 2-3 times
3 4 or more times

{SHOW F2 IF F1 > 0, OTHERWISE GO TO FILTER BEFORE DC1}

F2. In total, how much time have you spent reviewing your {DISPLAY PRE_1} results?

1 Less than 1 hour
2 1-2 hours
3 2-5 hours
4 5-10 hours
5 More than 10 hours

{PRG: SHOW IF PRE_1=4 or 5; OTHERWISE GO TO FILTER BEFORE DC3}

As science advances, researchers are able to learn additional information about the link between genetic variants and diseases and health conditions. Using this new information, it is possible for geneticists to create updated personal genome reports by comparing this newly discovered information to your genomic information.

DC1. Did you receive an updated personal genome report from Dr. Caskey?

1 Yes
2 No

{DESIGN: GRID DC2_1-DC2_4}
{PRG: SHOW IF DC1=1, OTHERWISE GO TO FILTER BEFORE DC3}

DC2. In general, how useful did you feel this updated personal genome report was to you?

1 Not at all useful
2 Not very useful
3 Somewhat useful
4 Very useful

Please explain why you feel this updated personal genome report was or was not useful.

[OPEN ENDED RESPONSE]

{DESIGN: GRID DC3_1-DC3_4}

{PRG: SHOW IF DC1=2, OTHERWISE SKIP TO DC4}
{PRG: SHOW IF PRE_1=3; OTHERWISE GO TO C0}

DC3. How interested would you be in receiving an updated personal genome report?

1 Not at all interested
2 Not very interested
3 Somewhat interested
4 Very interested

DC4. If you had to pay for updated personal genome reports, how much would you be willing to pay for an updated report?

________ U.S. Dollars [TEXT RESPONSE]

DC5. How often would you be interested in receiving an updated personal genome report if there were no cost to you?

1 Never
2 Every 6 months
3 Every year
4 Every 2 years
5 Every 5 years or more

{DESIGN: GRID B6}

B6. Please answer the following questions about your personal genome sequencing results.

1 Strongly Disagree
2 Disagree
3 Neither Agree nor Disagree
4 Agree
5 Strongly Agree

B6_1. Helped explain a condition that I have
B6_2. Helped explain a family history of disease
B6_3. Reassured me that I am healthy
B6_4. Gave me information about specific diseases that I am concerned about
B6_5. Helped tailor treatment(s) to me specifically
B6_6. Will help me prevent future diseases
B6_7. Helped me learn more about the risk of passing on a disease to my children or other descendants

---

{PRG: SHOW IF B6_4=4 or 5, OTHERWISE SKIP TO C0 GRID}

B6b. Please specify which disease(s) you are concerned about:

[OPEN ENDED RESPONSE]

{PRG: Do not show if PRE_1=4 OR 5}
{PRG: ASK C13 GRID IF ANY OF C0 IS SELECTED, AND PRESENT ONLY CORRESPONDING ROWS WHERE C0=1}

C13. Please indicate whether you ***opted to receive*** each of the following types of results as part of your personal genome sequencing report (i.e., your {RESPONSE:PRE_1} results).

1 Yes
2 No

C13_1. Information about my risk of a disease or diseases
C13_2. How my body responds to drugs or medications
C13_3. My carrier status
C13_4. Information about my ancestry
C13_5. Information explaining the cause of a disease I already knew I had
C13_6. My *APOE* genotype
C13_8. Physical traits (e.g. bitter tasting type)
C13_7. [TEXT RESPONSE FROM C0_OTH]

{PRG: SHOW IF C13_6=1; OTHERWISE SKIP TO FILTER BEFORE C1a}
{NOTE: EPSILON SYMBOL – HTML CODE **ε** }

C3a. What was your reported *APOE* genotype?

1 *APOE*-ε2/ε2
2 *APOE*-ε2/ε3
3 *APOE*-ε3/ε3
4 *APOE*-ε2/ε4
5 *APOE*-ε3/ε4
6 *APOE*-ε4/ε4
7 Genotype was not confidently reported
8 I prefer not to answer
9 I can't remember

{PRG: ASK C1A GRID IF ANY OF C13=1, AND PRESENT ONLY CORRESPONDING ROWS WHERE C13=1}

C1a. How interesting did you find each type of result that you received in your personal genome sequencing report through {DISPLAY PRE_1}?

1 Not at all interesting
2 Somewhat interesting
3 Very interesting

C1a_1. Information about my risk of a disease or diseases
C1a_2. How my body responds to drugs or medications
C1a_3. My carrier status
C1a_4. Information about my ancestry
C1a_5. Information explaining the cause of a disease I already knew I had
C1a_6. My *APOE* genotype
C1a_8. Physical traits (e.g. bitter tasting type)
C1a_7. [OTHER TEXT RESPONSE]

C2A_YN. Did you receive any personal information through {RESPONSE:PRE_1} that you felt was important to you?

1 Yes
2 No
3 Not sure

{PRG: ASK C2A_GRID IF C2A_YN=1}
{PRG: SOFT PROMPT IF NONE OF THE BELOW WERE ANSWERED}

C2A_GRID. Please list between one and three of the most important pieces of personal information that you received through {RESPONSE:PRE_1}.

C2a_GRID1 [TEXT RESPONSE]
C2a_GRID2 [TEXT RESPONSE]
C2a_GRID3 [TEXT RESPONSE]

{PRG: REPEAT C2A_1-C2A_6 FOR EACH RESPONSE PROVIDED; IF NO RESPONSES PROVIDED, THEN SKIP TO A1}

The following questions relate to [RESULT 1, RESULT 2, RESULT 3].

C2A[1|2|3]_1. Is this information related to a condition that you have or may develop in the future?

1 Yes
2 No

{PRG: SHOW IF C2A[1|2|3]_1=1; OTHERWISE TERMINATE THIS LOOP}

C2A[1|2|3]_2. Have you previously received a diagnosis related to this condition?

1 Yes
2 No

{PRG: SHOW IF C2A[1|2|3]_2=1; OTHERWISE SKIP TO C2A_4}

C2A[1|2|3]_3. How was this diagnosis made?

1 Based on my {RESPONSE:PRE_1} results alone
2 Based on my {RESPONSE:PRE_1} results in combination with my medical/family history
3 Based on my {RESPONSE:PRE_1} results in combination with medical tests/procedures that were performed because of my {RESPONSE:PRE_1} results
4 Other *(Please specify):* [TEXT RESPONSE]

C2A[1|2|3]_4. What do you think your chance is of developing this condition on a scale from 0-100%?

[NUMERIC RESPONSE 0-100]%

C2A[1|2|3]_5. Compared to the average person, what do you think your chance is of developing a condition related to this result?

1 Much higher
2 Somewhat higher
3 The same
4 Somewhat lower
5 Much lower

C2A[1|2|3]_6. How concerned are you about developing a condition related to this result?

1 Extremely concerned
2 Very concerned
3 Moderately concerned
4 Slightly concerned
5 Not at all concerned

A1. Have you discussed your {DISPLAY PRE_1} results with anyone new in the past year?

1 Yes
0 No

{PRG: SHOW A1_A IF A1 = 0 OTHERWISE GOTO FILTER BEFORE A2}

A1_A. Why have you **NOT** discussed your [DISPLAY PRE_1] results with anyone?

[OPEN ENDED RESPONSE]

{PRG: SHOW IF A1=1; OTHERWISE SKIP TO A3_A}

A2. With whom did you discuss your {DISPLAY PRE_1} results? *(Please select all that apply.)*

1 Family members
2 Friends
3 Co-workers/colleagues
4 Healthcare provider {PRG: IF PRE_1=4 OR 5, DISPLAY "(other than Dr. Caskey)"}
5 Contacts on social networking services (e.g., Facebook, Twitter)
6 Contacts on health- or disease-based social networking services (e.g., {PRG: IF PRE_1=1, DISPLAY "UYG Community,"} PatientsLikeMe, Cure Together, disease-specific patient networks)
7 Other *(Please specify):* [TEXT RESPONSE]

{PRG: SHOW A2_A IF A2 DOES NOT EQUAL 1, OTHERWISE GOTO A2_1A}

A2_A. Why have you **NOT** discussed your [DISPLAY PRE_1] results with family members? *(Please select all that apply.)*

1 I don't feel that my results are important enough to share with my family members.
2 I don't think family members are interested in my results.
3 I am concerned about how my family members might react to my results.
4 I plan to discuss my results with family members but haven't gotten around to it yet.
5 Other *(Please specify):* [TEXT RESPONSE]

{PRG: SHOW IF A2=1; OTHERWISE SKIP TO A3_A}
{PRG: SELECT ALL THAT APPLY}

A2_1a. With which family member(s) did you talk about your {DISPLAY PRE_1} results? *(Please select all that apply.)*

1 Spouse/significant other
2 Children
3 Brothers or sisters
4 Parents
5 Other relatives *(Please specify):* [TEXT RESPONSE]

{PRG: SHOW IF A2 DOES NOT=4 OR A1=0; OTHERWISE GO TO FILTER BEFORE A3_C}

A3_a. Why have you **NOT** discussed your [DISPLAY PRE_1] results with a healthcare provider {PRG: IF PRE_1=4 OR 5, DISPLAY “(other than Dr. Caskey)”}? *(Please select all that apply.)*

1 I have concerns about my genome sequencing results being placed in my medical record.
2 I don’t feel that my results are important enough to share with a healthcare provider.
3 I plan to discuss my results with a healthcare provider but haven’t gotten around to it yet.
4 I am confident in my ability to understand my results without the aid of a healthcare provider.
5 I do not think my healthcare provider is knowledgeable enough about genomic sequencing to use my results in my medical care.
6 I did not receive any results that required medical follow-up.
7 Other *(Please specify):* [TEXT RESPONSE]

{PRG: SHOW IF A2=4; OTHERWISE GO TO A7}

A3_c. With what type of healthcare provider(s) did you consult about your [DISPLAY PRE_1] results? *(Please select all that apply.)*

1 Primary care provider
2 Genetics specialist (e.g., genetic counselor or clinical geneticist)
3 Gastroenterologist
4 Anesthesiologist
5 Nutritionist
6 Obstetrician/gynecologist
7 Oncologist
8 Ophthalmologist
9 Physician assistant, nurse, or medical assistant
10 Reproductive endocrinologist
11 Surgeon
12 Pediatrician/child’s physician
13 Other specialist/healthcare provider *(Please specify):* [TEXT RESPONSE]

{DESIGN: A3_D & A3_E ON SAME PAGE}

A3_d. In what way(s) did you consult with your healthcare provider(s) about your [DISPLAY PRE_1} results? *(Please select all that apply.)*

1 Office visit
2 Telephone
3 Email
4 Other *(Please specify):* [TEXT RESPONSE]

A3_e. How much time did you spend with your healthcare provider(s) discussing your results in total?

1 Less than 30 minutes
2 Between 30 minutes and 1 hour
3 Between 1 and 2 hours
4 More than 2 hours

{DESIGN: GRID A3_F-A3_G}

How willing was/were your healthcare provider(s) to…

1 Not at all willing
2 Somewhat willing
3 Very willing

A3_f. …discuss the meaning of your {DISPLAY PRE_1} results?
A3_g. …use your [DISPLAY PRE_1] results in your medical care?

A3_OE. Comments:

[OPEN RESPONSE]

A3_h. Did the interpretation(s) of your results provided by your healthcare provider(s) differ from the interpretation provided by [DISPLAY PRE_1]?

1 Yes
0 No

{PRG: SHOW A3_HOPEN IF A3_H=1}

A3_HOPEN. How did the interpretations differ?

[OPEN RESPONSE]

{DESIGN: SHOW A3_I AND A3_J ON SAME PAGE}

A3_i. How satisfied were you with your discussion(s) of your {DISPLAY PRE_1} results with your healthcare provider(s)?

1 Not at all satisfied
2 Not very satisfied
3 Somewhat satisfied
4 Very satisfied

A3_j. Please explain why you were or were not satisfied with your discussion(s) of your [DISPLAY PRE_1] results with your healthcare provider(s).

[OPEN RESPONSE]

A7. Have your {DISPLAY PRE_1} results prompted you to seek out more information about health or medical topics related to your results?

1 Yes
0 No

{PRG: SAME SCREEN A7a-A7b}
{PRG: SHOW A7a-A7b IF A7=1; OTHERWISE SKIP TO A8}

A7a. What types of information have your {DISPLAY PRE_1} results prompted you to seek out?

[OPEN END RESPONSE]

A7b. Where did you get the information?

[OPEN END RESPONSE]

A8. Have your {DISPLAY PRE_1} results prompted you to **make an appointment** with any healthcare provider(s)?

2 Yes, I have already made an appointment.
1 I plan to make an appointment.
0 No, I do not plan to make an appointment.

{PRG: SHOW IF A8=1 or 2; OTHERWISE SKIP TO FILTER BEFORE A9a}

A8a. What type of healthcare provider(s) have your {DISPLAY PRE_1} results prompted you to make an appointment with? *(Please select all that apply.)*

1 Primary care provider
2 Genetics specialist (e.g., genetic counselor, clinical geneticist)
3 Gastroenterologist
4 Anesthesiologist
5 Nutritionist
6 Obstetrician/gynecologist
7 Oncologist
8 Ophthalmologist
9 Physician assistant, nurse, or medical assistant
10 Reproductive endocrinologist
11 Surgeon
12 Pediatrician/child's physician
13 Other specialist/healthcare provider *(Please specify):* [TEXT RESPONSE]

A8b. What was it about your [DISPLAY PRE_1] results that prompted you to make an appointment with any healthcare provider(s)? *(Please select all that apply.)*

1 There were results that I did not understand.
2 There was a result that required medical follow-up.
3 I wanted my results placed in my medical records.
4 Other *(Please specify):* [TEXT RESPONSE]

{PRG: SHOW A9a-A9b IF A2=4, OTHERWISE SKIP TO B1}
{PRG: SAME SCREEN A9a – A9b}

A9a. Which healthcare provider did you discuss your {DISPLAY PRE_1} results with the most?

[OPEN END RESPONSE]

Thinking about this healthcare provider with whom you discussed your {DISPLAY PRE_1} results the most, please indicate how much you agree or disagree with the following statement.

A9b. I believe that this healthcare provider understands genetics well enough to advise me on the implications of my {DISPLAY PRE_1} results for my health.

1 Strongly disagree
2 Somewhat disagree
3 Neither agree nor disagree
4 Somewhat agree
5 Strongly agree

B1. As a result of seeing your {DISPLAY PRE_1} results, have you spoken with a healthcare provider about any other tests, medical exams, or procedures?

1 Yes
0 No

{PRG: SHOW B1A IF B1=1, OTHERWISE GO TO B2}

B1a. What kind of tests, medical exams, or procedures have you spoken with a healthcare provider about as a result of seeing your {DISPLAY PRE_1} results? *(Please select all that apply.)*

1 One or more genetic tests to confirm a DNA sequence variant
2 One or more medical exams or procedures (other than genetic testing) to screen or test for a specific disease/condition
3 A whole body scan (i.e., an MRI in which images are taken of the whole body, not just one part)
4 Other *(Please specify):* [TEXT RESPONSE]

{PRG: SHOW B1B IF B1A = 1, OTHERWISE GO TO FILTER BEFORE B1C}

B1B. Please specify the genetic test(s) to confirm a DNA sequence variant that you spoke with a healthcare provider about as a result of seeing your {RESPONSE:PRE_1} results.

[MEMO RESPONSE]

{PRG: SHOW B1C IF B1A = 2, OTHERWISE GO TO B2}

B1C. Please specify the medical exams or procedures you spoke with a healthcare provider about as a result of seeing your {RESPONSE:PRE_1} results.

[MEMO RESPONSE]

B2. As a result of seeing your {DISPLAY PRE_1} results, have you **had** any tests, medical exams, or procedures?

1 Yes
0 No

{PRG: SHOW B2A IF B2=1, OTHERWISE GO TO C1}

B2a. What kind of tests, medical exams, or procedures have you **had** as a result of seeing your {DISPLAY PRE_1} results? (*Please select all that apply.)*

1 One or more genetic tests to confirm a DNA sequence variant
2 One or more medical exams or procedures (other than genetic testing) to screen or test for a specific disease/condition
3 A whole body scan (i.e., an MRI in which images are taken of the whole body, not just one part)
4 Other *(Please specify):* [TEXT RESPONSE]

{PRG: SHOW B2D & B2C IF B2A = 1, OTHERWISE GO TO FILTER BEFORE B2E}

B2d. Please specify the genetic test(s) to confirm a DNA sequence variant that you had as a result of seeing your {RESPONSE:PRE_1} results.

[MEMO RESPONSE]

B2c. Were the results of your genetic test(s) to confirm a DNA sequence variant consistent or inconsistent with your [DISPLAY PRE_1] results?

1 Consistent
2 Inconsistent
3 Results are still pending

{PRG: SHOW B2E IF B2A = 2, OTHERWISE GO TO C1}

B2e. Please specify the medical exams or procedures that you had as a result of seeing your {RESPONSE:PRE_1} results.

[MEMO RESPONSE]

TEXT: *Now we would like to ask you a few questions about your results and various health screens.*

C1. Blood cholesterol is a fatty substance found in the blood. Blood can be taken and used to determine your cholesterol level.

Because of your {DISPLAY PRE_1} results, have you had a blood test to check your cholesterol?

1 Yes
0 No
99 I don’t know

C2. A blood sugar test is a blood test that measures your blood glucose or blood sugar.

Because of your {DISPLAY PRE_1} results, have you had your blood sugar tested?

1 Yes
0 No
99 I don't know

C3_a. A colonoscopy/sigmoidoscopy is when a tube is inserted in the rectum to view the bowel for signs of cancer or other health problems. In this exam, all or part of the colon is checked. Anesthesia or pain medication is usually required.

Because of your {DISPLAY PRE_1} results, have you had a colonoscopy or sigmoidoscopy?

1 Yes
0 No
99 I don't know

C3_b. A fecal occult blood test (FOBT) or fecal immunochemical test (FIT) are two other tests used to screen for signs of colorectal cancer. For these tests, a stool sample is collected and checked for the presence of blood. There is no invasive testing involved, and no anesthesia or pain medications are required.

Because of your [DISPLAY PRE_1] results, have you had a FOBT or FIT test?

1 Yes
0 No
99 I don't know

C4. Because of your {DISPLAY PRE_1} results, have you had any tests in which a physician or healthcare provider looked for signs of heart disease, for example, an EKG (electrocardiogram) or a stress test?

1 Yes
0 No
99 I don't know

{PRG: SHOW C5-C7 IF DEM4b=1 OR 3 OR 4; OTHERWISE SKIP TO FILTER BEFORE C8}

C5. A mammogram is an x-ray of each breast to look for early signs of breast cancer.

Because of your {DISPLAY PRE_1} results, have you had a mammogram?

1 Yes
0 No
99 I don't know
2 {SHOW if DEM4b=3 or 4} N/A – Not applicable

C5a. MRI scans use magnets and radio waves instead of x-rays to produce very detailed, cross-sectional images of the body. MRI scans can take a long time-often up to an hour. You have to lie inside a narrow tube. For breast imaging, doctors inject a dye into a small vein in the arm before or during the exam.

Because of your {DISPLAY PRE_1} results, have you had a breast MRI scan?

1 Yes
0 No
99 I don't know
2 {SHOW if DEM4b=3 or 4} N/A – Not applicable

C6. A clinical breast exam is when a physician, nurse, or other healthcare provider feels your breasts for lumps. Because of your {DISPLAY PRE_1} results, have you had a clinical breast exam?

1 Yes
0 No
99 I don't know
2 {SHOW if DEM4b=3 or 4} N/A – Not applicable

C7. A Pap test, also called a Pap smear, is when a physician or other healthcare provider uses a special stick or brush to take a few cells from inside and around the cervix.

Because of your {DISPLAY PRE_1} results, have you had a Pap test?

1 Yes
0 No
99 I don't know
2 {SHOW if DEM4b=3 or 4} N/A – Not applicable

{PRG: SHOW IF DEM4b=2 OR 3 OR 4; OTHERWISE SKIP TO C12}

C8. A Prostate-Specific Antigen test, also called a PSA test, is a blood test used to check men for prostate cancer. Because of your {DISPLAY PRE_1} results, have you had a PSA test?

1 Yes
0 No
99 I don't know
2 {SHOW if DEM4b=3 or 4} N/A – Not applicable

C8a. A digital rectal exam (DRE) is when a physician or other healthcare provider inserts a gloved finger into the rectum to examine the prostate for irregularities in size, shape, or texture.

Because of your [DISPLAY PRE_1] results, have you had a DRE?

1 Yes
0 No
99 I don't know
2 {SHOW if DEM4b=3 or 4} N/A – Not applicable

C12. Because of your [DISPLAY PRE_1] results, have you had skin cancer (melanoma) screening (e.g., a physical examination of your skin to check for signs of cancer)?

1 Yes
0 No
99 I don't know

C9. Because of your {DISPLAY PRE_1} results, have you had any blood tests, imaging studies, or exams in which a physician or healthcare provider looked for signs of cancer, **other than those already listed above**?

1 Yes
0 No
99 I don't know

{PRG: SHOW C9a IF C9 = 1 OTHERWISE GO TO C11}

C9a. What other cancer screening tests/exams have you had because of your [DISPLAY PRE_1] results?

[MEMO RESPONSE]

C11. In total, how many medical visits with a physician or other healthcare provider {PRG: IF PRE_1=4 OR 5, DISPLAY "(other than Dr. Caskey)"} have you had as a direct result of receiving your {RESPONSE:PRE_1} results (in other words, visits you would <u>not</u> have had if you had <u>not</u> had personal genome sequencing)? *(If you are unsure, please provide your best estimate.)*

[NUMERIC RANGE 0-50] visits

G1_1a. Because of your {DISPLAY PRE_1} results, have you made any changes to your **diet**?

1 Yes
2 No
3 I don't know

{DESIGN: G1_1Y AND GRID G1_1B-G1_1D ON SAME PAGE}
{PRG: SHOW G1_1Y AND GRID G1_1B-G1_1D IF G1_1A=1, OTHERWISE SKIP TO G1_2A}

G1_1Y. What changes have you made to your **diet** because of your {DISPLAY PRE_1} results?

1 I have been eating more healthily.
2 I have been eating less healthily.
3 Other *(Please specify):* [TEXT RESPONSE]

G1_1. Was this change to your **diet**…

1 Yes
0 No

G1_1b. Self-motivated?
G1_1c. Recommended by a healthcare provider?
G1_1d. Recommended by [DISPLAY PRE_1]?

G1_2a. Because of your {DISPLAY PRE_1} results, have you made any changes to your **exercise routine or physical activity levels**?

1 Yes
2 No
3 I don't know

{DESIGN: G1_2Y AND GRID G1_2B-G1_2E ON SAME PAGE}
{PRG: SHOW G1_2Y AND GRID G1_2B-G1_2E IF G1_2A=1}

G1_2Y. What changes have you made to your **exercise routine or physical activity levels** because of your {DISPLAY PRE_1} results?

1 I have increased the amount I exercise.
2 I have decreased the amount I exercise.
3 Other *(Please specify):* [TEXT RESPONSE]

G1_2. Was this change to your **exercise routine or physical activity levels**…

1 Yes
0 No

G1_2b. Self-motivated?
G1_2c. Recommended by a healthcare provider?
G1_2d. Recommended by [DISPLAY PRE_1]?

G4. Because of your {DISPLAY PRE_1} results, have you made any changes to your **use of medications (including prescription and non-prescription) and/or supplements**?

1 Yes
2 No
3 I don't know

{PRG: SHOW G3 SERIES IF G4=1, OTHERWISE SKIP TO G2}

G3. How many changes have you made to any of your **medications (including prescription and non-prescription) and/or supplements** as a result of seeing your {DISPLAY PRE_1} results?

[NUMERIC RESPONSE 1-50] changes

{DESIGN: GRID G3A_1-G3A_3}
{PRG: FOR 0 < G3 <3, SHOW CORRESPONDING AMOUNT OF ROWS, IF G3 >3, ONLY SHOW 3 ROWS}

G3a. Please list the {PRG: IF G3>3, DISPLAY, "**three** most important"} medications/supplements that you made changes to.

G3a_1 [TEXT RESPONSE]
G3a_2 [TEXT RESPONSE]
G3a_3 [TEXT RESPONSE]

{DESIGN: G3B_1 –G3B_3}
{PRG: ONLY DISPLAY ROWS BELOW WHICH WERE ANSWERED IN G3A}

G3b. What type of medication/supplement is this?

1 Prescription medication
2 Non-prescription medication
3 Nutritional supplement
4 Alternative medicine
5 Other

G3b_1. {RESPONSE: G3a_1}
G3b_2. {RESPONSE: G3a_2}
G3b_3. {RESPONSE: G3a_3}

{DESIGN: G3C_1 –G3C_3}
{PRG: ONLY DISPLAY ROWS BELOW WHICH WERE ANSWERED IN G3A}

G3c. What changes did you make to this medication/supplement?

1 Stopped taking this medication/supplement
2 Lowered the dosage of this medication/supplement
3 Increased the dosage of this medication/supplement
4 Switched from this medication/supplement to another medication/supplement
5 Other

G3c_1. {RESPONSE: G3a_1}
G3c_2. {RESPONSE: G3a_2}
G3c_3. {RESPONSE: G3a_3}

{DESIGN: G3D_1-G3D_3}
{PRG: ONLY DISPLAY ROWS BELOW WHICH WERE ANSWERED IN G3A}

G3d. Did you consult with a healthcare provider prior to making changes to these medications/supplements?

1 Yes
0 No

G3d_1. {RESPONSE: G3a_1}
G3d_2. {RESPONSE: G3a_2}
G3d_3. {RESPONSE: G3a_3}

{PRG: INCLUDE G3E_INTRO – G3E_3 ON SAME PAGE.}
{PRG: ONLY DISPLAY ROWS BELOW WHICH WERE ANSWERED IN G3A}

G3e intro. We would like to know a bit more about why your {DISPLAY PRE_1} results prompted you to make this change to this medication/supplement. Please write any further information about this in the box below.

G3e_1. {RESPONSE: G3a_1} [MEMO]
G3e_2. {RESPONSE: G3a_2} [MEMO]
G3e_3. {RESPONSE: G3a_3} [MEMO]

G2. Do you think you will use your {DISPLAY PRE_1} results to guide your **future** use of medications/supplements?

1 Yes
0 No
99 I don't know

{PRG: SHOW THE FOLLOWING QUESTION IF L1=4 OR 5}

L1A. Earlier in the survey, you indicated that you used to smoke but that you quit smoking a while ago. Was your decision to quit smoking because of your {DISPLAY PRE_1} results?

1 Yes
2 No, I quit smoking before I received my {DISPLAY PRE_1} results.
3 No, I quit smoking after I received my {DISPLAY PRE_1} results, but my decision to quit smoking was not motivated by my {DISPLAY PRE_1} results.
4 I don't know

{PRG: SHOW IF L1A=1; OTHERWISE SKIP TO FILTER BEFORE L2A}
{DESIGN: GRID L1B_a-L1B_d}

L1B. Was your decision to quit smoking…

1 Yes
0 No

L1B_a. Self-motivated?
L1B_b. Recommended by a healthcare provider?
L1B_c. Recommended by [DISPLAY PRE_1]?

{PRG: SHOW IF L2=1 or 2}

L2a. Earlier in the survey, you indicated that you are planning to quit smoking. Is your plan to quit smoking because of your {DISPLAY PRE_1} results?

1 Yes
2 No
3 I don't know

{PRG: SHOW L2B IF L2A=1; OTHERWISE SKIP TO G1_3A}
{DESIGN: GRID L2B_a-L2B_d}

L2B. Is your plan to quit smoking…

1 Yes
0 No

L2B_a. Self-motivated?
L2B_b. Recommended by a healthcare provider?
L2B_c. Recommended by [DISPLAY PRE_1]?

G1_3a. Because of your {DISPLAY PRE_1} results, have you made changes to **any other aspects of your lifestyle or health behavior**, other than those already mentioned above?

1 Yes
2 No
3 I don't know

{DESIGN: G1_3Y AND GRID G1_3B-G1_3E ON SAME PAGE}
{PRG: SHOW G1_3Y AND GRID G1_3B-G1_3E IF G1_3A=1}
{DESIGN: GRID G1_3b-G1_3e}

G1_3Y. What **other aspects of your lifestyle or health behavior** have you changed because of your {DISPLAY PRE_1} results?

[TEXT RESPONSE]

G1_3. Was this change…

1 Yes
0 No

G1_3b. Self-motivated?
G1_3c. Recommended by a healthcare provider?
G1_3d. Recommended by [DISPLAY PRE_1]?

{PRG: IF H1= 9 OR 10, AND ALL H2-H4=0 OR 99, JUMP TO M2}

M1. Because of your {DISPLAY PRE_1} results, have you made any changes to your insurance coverage (i.e., health, life, long-term care, disability)?

1 Yes
0 No
2 Choose not to answer
99 Not sure

{PRG: SHOW M1A IF M1=1; OTHERWISE SKIP TO M1B}
{PRG: M1A SELECT ALL THAT APPLY}

M1a. To which insurance coverage did you make changes? *(Please select all that apply.)*

{PRG: SHOW IF H1≠9 OR 10}
1 Health insurance
{PRG: SHOW IF H2≠0 OR 99}
2 Life insurance
{PRG: SHOW IF H4≠0 OR 99}
3 Long-term care insurance
{PRG: SHOW IF H3≠0 OR 99}
4 Disability

{PRG: SHOW IF M1A=1; OTHERWISE SKIP TO FILTER BEFORE M1A_2}
{PRG: M1A_1 SELECT ALL THAT APPLY}

M1a_1. What changes did you make to your health insurance coverage? *(Please select all that apply.)*

1 Stopped coverage
2 Decreased existing coverage
3 Added coverage
4 Increased existing coverage
5 Other *(Please specify)*: [TEXT RESPONSE]

{PRG: SHOW IF M1A=2; OTHERWISE SKIP TO FILTER BEFORE M1A_3}
{PRG: M1A_2 SELECT ALL THAT APPLY}

M1a_2. What changes did you make to your life insurance coverage? *(Please select all that apply.)*

1 Stopped coverage
2 Decreased existing coverage
3 Added coverage
4 Increased existing coverage
5 Other *(Please specify)*: [TEXT RESPONSE]

{PRG: SHOW IF M1A=3; OTHERWISE SKIP TO FILTER BEFORE M1A_4}
{PRG: M1A_3 SELECT ALL THAT APPLY}

M1a_3. What changes did you make to your long-term care insurance coverage? *(Please select all that apply.)*

1 Stopped coverage
2 Decreased existing coverage
3 Added coverage
4 Increased existing coverage
5 Other *(Please specify)*: [TEXT RESPONSE]

{PRG: SHOW IF M1A=4; OTHERWISE SKIP TO FILTER BEFORE M1B}
{PRG: M1A_4 SELECT ALL THAT APPLY}

M1a_4. What changes did you make to your disability insurance coverage? *(Please select all that apply.)*

1 Stopped coverage
2 Decreased existing coverage
3 Added coverage
4 Increased existing coverage
5 Other *(Please specify)*: [TEXT RESPONSE]

M1b. Do you plan to make any {PRG: IF M1=1 DISPLAY “other”} changes to your insurance coverage because of your {DISPLAY PRE_1} results?

1 Yes
0 No
2 Choose not to answer
99 Not sure

{PRG: SHOW IF M1B=1; OTHERWISE SKIP TO M2}

M1b_1. What {PRG: IF M1=1 DISPLAY "other"} changes do you plan to make to your insurance coverage? *(Please select all that apply.)*

1	Stop coverage
2	Decrease existing coverage
3	Add coverage
4	Increase existing coverage
5	Other *(Please specify)*: [TEXT RESPONSE]

M2. Because of your {DISPLAY PRE_1} results, have you made any changes to your financial or retirement plans?

1	Yes
0	No
2	Choose not to answer
99	Not sure

{PRG: SHOW IF M2=1; OTHERWISE SKIP TO FILTER BEFORE M2B}

M2a. What changes have you made to your financial or retirement plans?

[OPEN END RESPONSE]

M2b. Do you plan to make any {PRG: IF M2=1 DISPLAY "other"} changes to your financial or retirement plans because of your {DISPLAY PRE_1} results?

1	Yes
0	No
2	Choose not to answer
99	Not sure

{PRG: SHOW M2B_1 IF M2B =1, OTHERWISE SKIP TO M3}

M2b_1. What {PRG: IF M2=1 DISPLAY "other"} changes do you plan to make to your financial or retirement plans?

[OPEN END RESPONSE]

M3. Because of your {DISPLAY PRE_1} results, have you made any changes related to other advanced planning (e.g., will, advance directives, power of attorney)?

1	Yes
0	No
2	Choose not to answer
99	Not sure

{PRG: SHOW IF M3=1; OTHERWISE SKIP TO FILTER BEFORE M3B}

M3a. What changes have you made related to advanced planning?

[OPEN END RESPONSE]

M3b. Do you plan to make any {PRG: IF M3=1 DISPLAY “other”} changes to your advanced planning because of your {DISPLAY PRE_1} results?

1	Yes
0	No
2	Choose not to answer
99	Not sure

{PRG: SHOW IF M3b=1; OTHERWISE SKIP TO F5}
{PRG: M3B_1 IS EXEMPT FROM SOFT PROMPT}

M3b_1. What {PRG: IF M3=1 DISPLAY “other”} changes do you plan to make to your advanced planning?

[OPEN END RESPONSE]

{Note: Section Header 4: Your Genome Results Report: Attitudes}

Invitae Participant Note {PRG: DO NOT SHOW}
{PRG: SHOW ON ALL PAGES IF PRE_1=9 (INVITAE)}
Invitae Participants: In this survey, ***genome*** refers to ***genetic***, ***genome sequencing*** refers to ***genetic testing***, and ***genome reports*** refer to ***genetic test reports***.

{DESIGN: GRID F5a-F5e}

F5. To what extent do you agree or disagree with the following statements?

1 Strongly disagree
2 Somewhat disagree
3 Neither agree nor disagree
4 Somewhat agree
5 Strongly agree

F5a. The information I received about my genome will influence how I manage my health in the future.
F5b. Having personal genome sequencing made me feel like I have more control over my health.
F5d. Having personal genome sequencing helped me to get a better perspective on my health status.
F5e. What I learned from my personal genome sequencing will help reduce my chances of getting sick.

{DESIGN: GRID F6A-F6P}
{PRG: ONLY SHOW F6D IF PRE_1=1; OTHERWISE DO NOT INCLUDE}
{PRG: ONLY SHOW F6H IF PRE_1=2; OTHERWISE DO NOT INCLUDE}
{PRG: ONLY SHOW F6M & F6Q IF K8=7; OTHERWISE DO NOT INCLUDE}

F6. To what extent do you agree or disagree with the following statements?

1 Strongly disagree
2 Somewhat disagree
3 Neither agree nor disagree
4 Somewhat agree
5 Strongly agree

F6a. I am confident in the quality and accuracy of my {DISPLAY PRE_1} results.
F6i. Through [DISPLAY PRE_1], I learned something to improve my health that I didn't know before.
F6j. I am disappointed that my {DISPLAY PRE_1} results did not tell me more information.
F6k. I found my {DISPLAY PRE_1} results interesting.
F6l. I found the information from {DISPLAY PRE_1} to be fun and entertaining.
F6d. I feel that I got what I paid for through {RESPONSE: PRE_1}.
F6h. I would have utilized in-person genetic counseling services had they been available through {RESPONSE:PRE_1}.
F6c. The educational materials provided by {RESPONSE:PRE_1} were helpful.
F6m. The knowledge I gained about genome sequencing through {RESPONSE:PRE_1} has influenced my medical practice.
F6q. The knowledge I gained about genome sequencing through {RESPONSE:PRE_1} has influenced my clinical research.

{PRG: SHOW IF F6m= 4 OR 5; OTHERWISE SKIP TO B4}

F6m_1. How has the knowledge you gained about genome sequencing through {RESPONSE:PRE_1} influenced your professional practice? (*Please select all that apply.)*

1 It has made me MORE likely to consider genome sequencing for one or more of my patients.
2 It has made me LESS likely to consider genome sequencing for one or more of my patients.
3 It has helped me better understand the benefits of the technology.
4 It has helped me better understand the limitations of the technology.
5 It has helped me better understand the risks of the technology.
6 It has affected my research methods or priorities.
7 It has changed my views on professional guidelines and/or regulations.
8 Other *(Please specify):* [TEXT RESPONSE]

{PRG: SHOW IF F6M_1=6; OTHERWISE SKIP TO FILTER BEFORE F6_M3}

F6_m2. How has the knowledge you gained about genome sequencing through this process affected your research methods or priorities?

[OPEN END RESPONSE]

{PRG: SHOW IF F6M_1=7; OTHERWISE SKIP TO B4}

F6_m3. How has the knowledge you gained about genome sequencing through this process changed your views on professional guidelines and/or regulations?

[OPEN END RESPONSE]

B4. *Please read the following questions and for each one select the response that best applies to you:*

Do you feel the information you have received so far through your personal genome sequencing results with {DISPLAY PRE_1} will…

1 No, definitely not
2 No, probably not
3 Yes, probably
4 Yes, definitely

B4b. …influence what treatment you receive for current or future medical problems?
B4c. …influence decisions you make about your medical care?
B4d. …influence your reproductive decisions?
B4e. …influence what medications you take?
B4f. …influence your end of life planning (e.g., whether you get or change your advanced directive/living will)?

M5. How much do you agree or disagree with the following statements?

1 Strongly disagree
3 Somewhat disagree
4 Neither agree nor disagree
5 Somewhat agree
7 Strongly agree

M5a. I am confident that I understand my {RESPONSE:PRE_1} results.
M5b. It has been easy for me to get information about what my {RESPONSE:PRE_1} results mean.
M5c. I understand what my {RESPONSE:PRE_1} results mean for my health.
M5d. I understand how the DNA changes identified in my {RESPONSE:PRE_1} results may influence my disease risk.
M5e. I understand how my own genetic make-up might affect my disease risk.
M5f. I am able to explain to others what my {RESPONSE:PRE_1} results mean for my health.

{PRG: SHOW IF K3a>0}

INTRO: *The next questions are about some feelings you may have had before or after receiving your personal genome sequencing results.*

PG1. Do you think you may have passed on any genetic diseases or conditions to your children and/or grandchildren?

1 Yes
0 No
2 I don't know

{PRG: SHOW PG9 IF PG1=1, OTHERWISE SKIP TO B5}

PG9. How guilty do you feel about possibly passing on genetic risk to your children or grandchildren?

1 Not at all guilty
2 Not very guilty
3 Somewhat guilty
4 Very guilty
5 Not applicable

{PRG: SHOW PG10 IF PG9=3 OR 4, OTHERWISE SKIP TO B5}

PG10. Was this feeling of guilt prompted by your {RESPONSE:PRE_1} results?

1 Yes
0 No

{PRG: SHOW PG11 IF PG10=1, OTHERWISE SKIP TO B5}

PG11. What about your {RESPONSE:PRE_1} results caused you to feel guilty?

[OPEN END RESPONSE]

{PRG: USE LIKERT SCALE FORMAT FOR B5-B6 AND PLACE ON THE SAME PAGE}

B5. On a scale of 1 to 10, how useful do you think your personal genome sequencing results through {DISPLAY PRE_1} are to you **now**?

1 Not at all useful – 10 Extremely useful

B6. On a scale of 1 to 10, how useful do you think your personal genome sequencing results through {DISPLAY PRE_1} will be to you **in the future**?

1 Not at all useful – 10 Extremely useful

{DESIGN: SHOW GRID F4 AND F4B ON SAME PAGE}
{DESIGN: GRID F4}

F4. In general, how valuable do you feel your personal genome sequencing experience with {DISPLAY PRE_1} was to you?

1 Not at all valuable
2 Not very valuable
3 Somewhat valuable
4 Very valuable

F4b. Please explain why you feel the personal genome sequencing experience with {DISPLAY PRE_1} was or was not valuable.

[OPEN END RESPONSE]

{NOTE: SECTION HEADER 5: Your Entire Genome Sequence}

Invitae Participant Note {PRG: DO NOT SHOW}
{PRG: SHOW ON ALL PAGES IF PRE_1=9 (INVITAE)}
Invitae Participants: In this survey, ***genome*** refers to ***genetic***, ***genome sequencing*** refers to ***genetic testing***, and ***genome reports*** refer to ***genetic test reports***.

INTRO: *In the following section, we are interested in your views on your entire genome sequence, rather than the results report or interpretation. Your "entire genome sequence" is the list of around 3 billion As, Cs, Gs and Ts (or the proportion of this that was successfully sequenced in your case) that make up your genome.*

DS1. In general, how comfortable are you with the idea of sharing your entire genome sequence (not just the report or interpretation)?

1 Very comfortable
2 Somewhat comfortable
3 Somewhat uncomfortable
4 Very uncomfortable

{DESIGN: GRID DS2}

DS2. Would you be willing to share your entire genome sequence…

1 Yes
2 No
3 Not sure

DS2a. …with scientific researchers, as long as your identity remained anonymous?
DS2b. …with scientific researchers, with your identity attached to it?
DS2c. …publicly, as long as your identity remained anonymous?
DS2d. …publicly, with your identity attached to it?

DS3. If someone had access to your entire genome sequence (not just the report or interpretation), and nothing else, how easy or difficult do you think it would be for them to discover your identity?

1 Very easy
2 Quite easy
3 Quite difficult
4 Very difficult
5 Impossible

A10. Through {DISPLAY PRE_1}, do you have access to your entire genome sequence (not just the report or interpretation)?

1 Yes
2 No
3 I don't know

{PRG: SHOW IF A10=2 OR 3; OTHERWISE SKIP TO FILTER BEFORE A12}
{DESIGN: DISPLAY A11A AND A11B ON SAME SCREEN}

A11a. If it were available, would you be interested in receiving your entire genome sequence (not just the report or interpretation)?

1 Yes
2 No

A11b. Please explain why you would or would not like to receive your entire genome sequence.

[TEXT RESPONSE]

{PRG: SHOW IF A10=1; OTHERWISE SKIP TO F6}

A12. Have you explored your entire genome sequence (not just the report or interpretation) on your own?

1 Yes
2 No

{PRG: SHOW A12A IF A12=2}

A12a. Why have you **NOT** explored your entire genome sequence (not just the report or interpretation) on your own? *(Please select all that apply.)*

1 I have not needed it for anything.
2 I would like to, but have not had the time yet.
3 I am not technically able to do so.
4 I am not interested in doing so on my own.
5 Other *(Please specify):* [TEXT RESPONSE]

{PRG: SHOW DS4 IF A12=1, ELSE GO TO F6}

DS4. Have you conducted or asked others to conduct any additional interpretations of your entire genome sequence?

1 Yes
2 No
3 I do not have access to this type of data
4 I do not know

PRG: SHOW DS4A AND DS4C IF DS4=1, OTHERWISE SKIP TO F6}

DS4a. How much do you trust these additional interpretations?

1 Not at all
2 Not very much
3 Moderately
4 Completely

DS4c. What were these additional interpretations and how will you use them?

[OPEN RESPONSE]

{Note: Section Header 6: General Attitudes Towards Genomics}

Invitae Participant Note {PRG: DO NOT SHOW}
{PRG: SHOW ON ALL PAGES IF PRE_1=9 (INVITAE)}
Invitae Participants: In this survey, ***genome*** refers to ***genetic***, ***genome sequencing*** refers to ***genetic testing***, and ***genome reports*** refer to ***genetic test reports***.

Intro: *In this section, we are interested in learning about your attitudes and thoughts about genome sequencing in general.*

{PRG: GRID F6E – F6P}

F6. Please indicate whether you agree or disagree with each of the following statements.

1 Strongly disagree
2 Somewhat disagree
3 Neither agree nor disagree
4 Somewhat agree
5 Strongly agree

F6e. Health insurance should cover personal genome sequencing.
F6f. Personal genomic information should be part of a standard medical record.
F6g. Parents should be able to get personal genome sequencing for their children if they want to.
F6n. The government should put more effort into regulating personal genome sequencing.
F6o. Personal genome sequencing should only be available to people through their doctor.
F6p. People have a right to access their own personal genomic information without going through a healthcare provider.

M4. Please indicate whether you agree or disagree with each of the following statements.

1 Strongly disagree
2 Somewhat disagree
3 Neither agree nor disagree
4 Somewhat agree
5 Strongly agree

M4a. Once a variant in a gene that affects a person's risk of a disease is found, that disease can always be prevented or cured.
M4b. A healthcare provider can tell a person their exact chance of developing a disease based on the results from genome sequencing.
M4c. Scientists know how all variants of genes will affect a person's chances of developing diseases.
M4d. Even if a person has a variant in a gene that affects their risk of disease, they may not develop that disease.
M4e. Genome sequencing is a routine test that most people can have through their physician's office.
M4f. Genome sequencing may find variants in a person's genes that they can pass on to their children.
M4g. Genome sequencing may give a person information about their chances of developing several different diseases.
M4h. Genome sequencing may find variants in a person's genes that will increase their chance of developing a disease in their lifetime.
M4i. Genome sequencing may find variants in a person's genes that will decrease their chance of developing a disease in their lifetime.
M4j. Genome sequencing may find variants in a person's genes that may determine how they respond to certain medicines.
M4k. A person's health habits, such as diet and exercise, can affect whether or not their genes cause diseases such as heart disease and cancer.

{Note: Section Header 7: A Few More Questions About You}

Invitae Participant Note {PRG: DO NOT SHOW}
{PRG: SHOW ON ALL PAGES IF PRE_1=9 (INVITAE)}
Invitae Participants: In this survey, ***genome*** refers to ***genetic***, ***genome sequencing*** refers to ***genetic testing***, and ***genome reports*** refer to ***genetic test reports***.

INTRO: *In this section, we are interested in learning a bit more about you, your health, and the health of your family.*

K12. Would you say that in general your health is:

1 Excellent
2 Very good
3 Good
4 Fair
5 Poor

{DESIGN: GRID C3_1 TO C3_14}

C3. Has a healthcare provider ever told you that you have (or had) any of the following medical conditions?

1 Yes
0 No

C3_1. Arthritis
C3_2. Asthma
C3_3. Cancer
C3_4. Chronic kidney disease
C3_5. Diabetes
C3_6. Eye conditions
C3_7. Gastrointestinal (GI) conditions
C3_8. Heart conditions
C3_9. High cholesterol
C3_10. Lupus
C3_11. Mental illness/psychiatric conditions
C3_12. Neurological conditions (e.g., Alzheimer’s disease, Multiple sclerosis, Parkinson’s disease)
C3_13. Psoriasis
C3_14. Bleeding disorder

{DESIGN: GRID C4_1-C4_14}

C4. Have any of your blood relatives (a parent, brother or sister, child, grandparent, aunt, uncle, or first cousin) ever had any of the following conditions?

1 Yes
0 No

C4_1 Arthritis
C4_2 Asthma
C4_3 Cancer
C4_4 Chronic kidney disease
C4_5 Diabetes
C4_6 Eye conditions
C4_7 Gastrointestinal (GI) conditions
C4_8 Heart conditions
C4_9 High cholesterol
C4_10 Lupus
C4_11 Mental illness/psychiatric conditions
C4_12 Neurological conditions (e.g., Alzheimer's disease, ALS, Multiple sclerosis, Parkinson's disease)
C4_13 Psoriasis
C4_14 Bleeding disorder

{PRG: SHOW C5 GRID IF ANY OF C4_1 - C4_14 = 1, OTHERWISE SKIP TO E1}
{DESIGN: GRID C5_1-C5_15}
{PRG: SELECT ALL THAT APPLY}

C5. Which of your blood relatives (a parent, brother or sister, child, grandparent, aunt, uncle, or first cousin) have ever had any of the following conditions? (*Please select all that apply.*)

1 A parent
2 A brother or sister
3 A child
4 A grandparent
5 An aunt, uncle, or first cousin

{PRG: SHOW C5_1 IF C4_1 = 1}	C5_1.	Arthritis
{PRG: SHOW C5_2 IF C4_2 = 1}	C5_2.	Asthma
{PRG: SHOW C5_3 IF C4_3 = 1}	C5_3.	Cancer
{PRG: SHOW C5_4 IF C4_4 = 1}	C5_4.	Chronic kidney disease
{PRG: SHOW C5_5 IF C4_5 = 1}	C5_5.	Diabetes
{PRG: SHOW C5_6 IF C4_6 = 1}	C5_6.	Eye conditions
{PRG: SHOW C5_7 IF C4_7 = 1}	C5_7.	Gastrointestinal (GI) conditions
{PRG: SHOW C5_8 IF C4_8 = 1}	C5_8.	Heart conditions
{PRG: SHOW C5_9 IF C4_9 = 1}	C5_9.	High cholesterol
{PRG: SHOW C5_10 IF C4_10 = 1}	C5_10.	Lupus
{PRG: SHOW C5_11 IF C4_11 = 1}	C5_11.	Mental illness/psychiatric conditions
{PRG: SHOW C5_12 IF C4_12 = 1}	C5_12.	Neurological conditions (e.g., Alzheimer's disease, ALS, Multiple sclerosis, Parkinson's disease)
{PRG: SHOW C5_13 IF C4_13 = 1}	C5_13.	Psoriasis
{PRG: SHOW C5_14 IF C4_14 = 1}	C5_14.	Bleeding disorder

{Note: DK should NOT be mutually exclusive in following grids}

{PRG: SHOW C5_1a_1 - C5_1a_5 GRID IF C5_1= 1-5, OTHERWISE SKIP TO FILTER BEFORE C5_3a_1- C5_3a_5 GRID}

{DESIGN: GRID C5_1a_1 - C5_1a_5}
{PRG: C5_1a_1 - C5_1a_5 SELECT ALL THAT APPLY}

Please select the type(s) of {PRG: IF PRE_1=1 DISPLAY "**arthritis**"; OTHERWISE DISPLAY "**arthritis/immune disorders**"} that each of the following relatives has/had. *(Please select all that apply.)*

1 Osteoarthritis ("wear and tear" on joints)
2 Rheumatoid arthritis (joint swelling and stiffness)
3 Other
99 I don't know

{PRG: SHOW C5_1a_1 IF C5_1=1} C5_1a_1. A parent
{PRG: SHOW C5_1a_2 IF C5_1=2} C5_1a_2. A brother or sister
{PRG: SHOW C5_1a_3 IF C5_1=3} C5_1a_3. A child
{PRG: SHOW C5_1a_4 IF C5_1=4} C5_1a_4. A grandparent
{PRG: SHOW C5_1a_5 IF C5_1=5} C5_1a_5. An aunt, uncle, or first cousin

---

{PRG: SHOW C5_3a_1 - C5_3a_5 GRID IF C5_3= 1-5, OTHERWISE SKIP TO FILTER BEFORE C5_5a_1- C5_5a_5 GRID}

{DESIGN: GRID C5_3a_1 - C5_3a_5}
{PRG: C5_3a_1 - C5_3a_5 SELECT ALL THAT APPLY}

Please select the type(s) of **cancer** that each of the following relatives has/had. *(Please select all that apply.)*

1 Breast cancer
2 Colorectal cancer
3 Esophageal cancer
4 Leukemia
5 Lung cancer
6 Prostate cancer
7 Skin cancer (Melanoma)
8 Stomach cancer
9 Other
99 I don't know

{PRG: SHOW C5_3a_1 IF C5_3=1} C5_3a_1. A parent
{PRG: SHOW C5_3a_2 IF C5_3=2} C5_3a_2. A brother or sister
{PRG: SHOW C5_3a_3 IF C5_3=3} C5_3a_3. A child
{PRG: SHOW C5_3a_4 IF C5_3=4} C5_3a_4. A grandparent
{PRG: SHOW C5_3a_5 IF C5_3=5} C5_3a_5. An aunt, uncle, or first cousin

---

{PRG: SHOW C5_5a_1 - C5_5a_5 GRID IF C5_5 = 1-5, OTHERWISE SKIP TO FILTER BEFORE C5_6a_1- C5_6a_5 GRID}

{DESIGN: GRID C5_5a_1 - C5_5a_5}
{PRG: C5_5a_1 - C5_5a_5 SELECT ALL THAT APPLY}

Please select the type(s) of **diabetes** that each of the following relatives has/had. *(Please select all that apply.)*

1 Type 1 (insulin dependent, juvenile onset)
2 Type 2 (non-insulin dependent)
99 I don't know

{PRG: SHOW C5_5a_1 IF C5_5=1} C5_5a_1. A parent
{PRG: SHOW C5_5a_2 IF C5_5=2} C5_5a_2. A brother or sister
{PRG: SHOW C5_5a_3 IF C5_5=3} C5_5a_3. A child
{PRG: SHOW C5_5a_4 IF C5_5=4} C5_5a_4. A grandparent
{PRG: SHOW C5_5a_5 IF C5_5=5} C5_5a_5. An aunt, uncle, or first cousin

---

{PRG: SHOW C5_6a_1 - C5_6a_5 GRID IF C5_6= 1-5, OTHERWISE SKIP TO FILTER BEFORE C5_7a_1- C5_7a_5 GRID}

{DESIGN: GRID C5_6a_1 - C5_6a_5}
{PRG: C5_6a_1 - C5_6a_5 SELECT ALL THAT APPLY}

Please select the type(s) of **eye conditions** that each of the following relatives has/had. *(Please select all that apply.)*

1 Age-related macular degeneration
2 Glaucoma
3 Cataracts
4 Other
99 I don't know

{PRG: SHOW C5_6a_1 IF C5_6=1} C5_6a_1. A parent
{PRG: SHOW C5_6a_2 IF C5_6=2} C5_6a_2. A brother or sister
{PRG: SHOW C5_6a_3 IF C5_6=3} C5_6a_3. A child
{PRG: SHOW C5_6a_4 IF C5_6=4} C5_6a_4. A grandparent
{PRG: SHOW C5_6a_5 IF C5_6=5} C5_6a_5. An aunt, uncle, or first cousin

---

{PRG: SHOW C5_7a_1 - C5_7a_5 GRID IF C5_7 = 1-5, OTHERWISE SKIP TO FILTER BEFORE C5_8a_1- C5_8a_5 GRID}

{DESIGN: GRID C5_7a_1 - C5_7a_5}
{PRG: C5_7a_1 - C5_7a_5 SELECT ALL THAT APPLY}

Please select the type(s) of **gastrointestinal (GI) conditions** that each of the following relatives has/had. *(Select all that apply.)*

1 Celiac disease
2 Crohn's disease
3 Ulcerative colitis
4 Other
99 I don't know

{PRG: SHOW C5_7a_1 IF C5_7=1} C5_7a_1. A parent
{PRG: SHOW C5_7a_2 IF C5_7=2} C5_7a_2. A brother or sister
{PRG: SHOW C5_7a_3 IF C5_7=3} C5_7a_3. A child
{PRG: SHOW C5_7a_4 IF C5_7=4} C5_7a_4. A grandparent
{PRG: SHOW C5_7a_5 IF C7_4=5} C5_7a_5. An aunt, uncle, or first cousin

---

{PRG: SHOW C5_8a_1 - C5_8a_5 GRID IF C5_8 = 1-5, OTHERWISE SKIP TO FILTER BEFORE C5_11a_1- C5_11a_5 GRID}

{DESIGN: GRID C5_8a_1 - C5_8a_5}
{PRG: C5_8a_1 - C5_8a_5 SELECT ALL THAT APPLY}

Please select the type(s) of **heart conditions** that each of the following relatives has/had. *(Please select all that apply.)*

1 Irregular heartbeat (Atrial fibrillation)
2 Coronary artery disease
3 Peripheral arterial disease
4 Blood clotting (Venous thromboembolism)
5 Other
99 I don't know

{PRG: SHOW C5_8a_1 IF C5_8=1} C5_8a_1. A parent
{PRG: SHOW C5_8a_2 IF C5_8=2} C5_8a_2. A brother or sister
{PRG: SHOW C5_8a_3 IF C5_8=3} C5_8a_3. A child
{PRG: SHOW C5_8a_4 IF C5_8=4} C5_8a_4. A grandparent
{PRG: SHOW C5_8a_5 IF C5_8=5} C5_8a_5. An aunt, uncle, or first cousin

---

{PRG: SHOW C5_11a_1 - C5_11a_5 GRID IF C5_11= 1-5, OTHERWISE SKIP TO FILTER BEFORE C5_12a_1 - C5_12a_5 GRID}

{DESIGN: GRID C5_11a_1 - C5_11a_5}
{PRG: C5_11a_1 - C5_11a_5 SELECT ALL THAT APPLY}

Please select the type(s) of **mental illness/psychiatric conditions** that each of the following relatives has/had. *(Please select all that apply.)*

3 Anxiety disorder
1 Bipolar disorder
2 Depression
4 Other
99 I don't know

{PRG: SHOW C5_11a_1 IF C5_11=1} C5_11a_1. A parent
{PRG: SHOW C5_11a_2 IF C5_11=2} C5_11a_2. A brother or sister
{PRG: SHOW C5_11a_3 IF C5_11=3} C5_11a_3. A child
{PRG: SHOW C5_11a_4 IF C5_11=4} C5_11a_4. A grandparent
{PRG: SHOW C5_11a_5 IF C5_11=5} C5_11a_5. An aunt, uncle, or first cousin

---

{PRG: SHOW C5_12a_1 - C5_12a_5 GRID IF C5_12= 1-5, OTHERWISE SKIP TO E1}

{DESIGN: GRID C5_12a_1 - C5_12a_5}
{PRG: C5_12a_1 - C5_12a_5 SELECT ALL THAT APPLY}

Please select the type(s) of **neurological conditions** that each of the following relatives has/had. *(Please select all that apply.)*

1 Alzheimer's disease
2 ALS (Lou Gehrig's disease)
3 Multiple sclerosis
4 Parkinson's disease
5 Other
99 I don't know

{PRG: SHOW C5_12a_1 IF C5_12=1} C5_12a_1. A parent
{PRG: SHOW C5_12a_2 IF C5_12=2} C5_12a_2. A brother or sister
{PRG: SHOW C5_12a_3 IF C5_12=3} C5_12a_3. A child
{PRG: SHOW C5_12a_4 IF C5_12=4} C5_12a_4. A grandparent
{PRG: SHOW C5_12a_5 IF C5_12=5} C5_12a_5. An aunt, uncle, or first cousin

---

{PRG: SHOW C5_14a_1 - C5_14a_5 GRID IF C5_14= 1-5, OTHERWISE SKIP TO E1}

{DESIGN: GRID C5_14a_1 - C5_14a_5}
{PRG: C5_14a_1 - C5_14a_5 SELECT ALL THAT APPLY}

Please select the type(s) of bleeding disorders that each of the following relatives has/had. *(Please select all that apply.)*

1 Von Willebrand Disease
2 Hemophilia A (Factor VIII Deficiency)
3 Hemophilia B (Congenital Factor IX Deficiency)
4 Congenital Fibrinogen (Factor I) Deficiency
5 Congenital Prothrombin (Factor II) Deficiency
6 Congenital Factor VII Deficiency
7 Congenital Factor X Deficiency
8 Congenital Factor XIII Deficiency
9 Other
99 I don't know

{PRG: SHOW C5_14a_1 IF C5_14=1} C5_14a_1. A parent
{PRG: SHOW C5_14a_2 IF C5_14=2} C5_14a_2. A brother or sister
{PRG: SHOW C5_12a_3 IF C5_14=3} C5_14a_3. A child
{PRG: SHOW C5_14a_4 IF C5_14=4} C5_14a_4. A grandparent
{PRG: SHOW C5_14a_5 IF C5_14=5} C5_14a_5. An aunt, uncle, or first cousin

---

{DESIGN: GRID E1_1-E_12}

E1. Compared to the average person of your age, what would you say your chances are of developing these conditions?

1 Much lower than average
2 Lower than average
3 Average
4 Higher than average
5 Much higher than average
6 I already have this condition

E1_1. Alzheimer's disease
{PRG: SHOW E1_2 IF DEM4b=1 OR 3 OR 4}
E1_2. Breast cancer
{PRG: SHOWE1_3 IF DEM4b=2 OR 3 OR 4}
E1_3. Prostate cancer
E1_4. Colorectal cancer
E1_5. Lung cancer
E1_6. Diabetes
E1_7. Heart disease (Coronary artery disease)
E1_8. Parkinson's disease

{DESIGN: GRID D1a-D1f}

Now we are going to ask you some questions about yourself and how you have been feeling.

D1. *Please read the following statements and for each one select the response that best applies to you.*

Over the **past two weeks**, how often have you:

1 Not at all
2 Several days
3 More than half of the days
4 Nearly every day

D1a. Felt nervous, anxious, or on edge?
D1b. Been unable to stop or control worrying?
D1c. Felt calm and peaceful?
D1d. Been a happy person?
D1e. Had little interest or pleasure in doing things?
D1f. Felt down, depressed, or hopeless?

E2. You will find below a series of statements that describe how people may react to the uncertainties of life. Please use the scale below to describe to what extent each item is characteristic of you.

1 Not at all characteristic of me 1
2 2
3 3
4 4
5 Entirely characteristic of me 5

E2_1. Unforeseen events upset me greatly.
E2_2. It frustrates me not having all the information I need.
E2_3. Uncertainty keeps me from living a full life.
E2_4. One should always look ahead so as to avoid surprises.
E2_5. A small, unforeseen event can spoil everything, even with the best planning.
E2_6. When it's time to act, uncertainty paralyses me.
E2_7. When I am uncertain, I can't function very well.
E2_8. I always want to know what the future has in store for me.
E2_9. I can't stand being taken by surprise.
E2_10. The smallest doubt can stop me from acting.
E2_11. I should be able to organize everything in advance.
E2_12. I must get away from all uncertain situations.

K13. Please select the statement that best describes the role you prefer to take in dealing with your health care:

1 I prefer to make the final decision about which treatment I will receive.
2 I prefer to make the final decision about my treatment after seriously considering my doctor's opinion.
3 I prefer that my doctor and I share responsibility for deciding which treatment is best for me.
4 I prefer that my doctor make the final decision about which treatment I will receive, but seriously consider my opinion.
5 I prefer to leave all decisions regarding my treatment to my doctor.

{NOTE: SECTION HEADER 8: Wrap-Up}

Invitae Participant Note {PRG: DO NOT SHOW}
{PRG: SHOW ON ALL PAGES IF PRE_1=9 (INVITAE)}
Invitae Participants: In this survey, ***genome*** refers to ***genetic***, ***genome sequencing*** refers to ***genetic testing***, and ***genome reports*** refer to ***genetic test reports***.

You are almost done with the survey! Here is the final set of questions.

{PRG: S2G IS EXEMPT FROM SOFT PROMPT}

S2GA. Is there anything else you would like to share about your experience of participating in {RESPONSE:PRE_0}?

[OPEN ENDED RESPONSE]

S2G. Is there anything else you would like to share about your experience of participating in the Personal Genome Sequencing Outcomes Study thus far?

[OPEN ENDED RESPONSE]

{PRG: SHOW IF PRE_1=2, OTHERWISE SKIP TO DEM6}

DEM2b. What is your PGP ID (huID)?
This identifier is visible in the top right of your account when you're logged in to my.pgp-hms.org. Sharing this will share with our study any current or future genome and health data you publicly share in your PGP profile.

[TEXT RESPONSE validate with regex: ^[hH][uU][0-9A-Fa-f]{6}$]

{PRG: ADD FOLLOWING AS SOFT PROMPT WITH RESPONSE CATEGORIES}

DEM2b_PROMPT. An important aspect of this study is to be able to link your survey results with your personal genome sequencing results. Doing this will give us insight into how people use personal genomic information and how genome sequencing may (or may not) influence their lives. While we can use your survey response without linking it, providing this ID now will allow us to maximize the benefit of this research.

Again, this identifier is visible in the top right of your account when you’re logged in to my.pgp-hms.org. You will not be able to make this link with this study at a later time.

Please indicate your wishes below:

1 No thank you, I choose not to provide my PGP ID (huID)
2 My PGP ID (huID) is [TEXT RESPONSE]
3 I allow this study team to obtain my PGP ID (huID) and research report from The Harvard Personal Genome Project (PGP)

{PRG: SHOW IF PRE_1=1; OTHERWISE SKIP TO DEM6}

CONSENT FOR RELEASE OF GENOME REPORTS:

Thank you for contributing to this research study.

We would like to be able to link your responses to your clinical test reports and MyGenome Application provided to you by Illumina, Inc.

By sharing your clinical test report and granting access to your genome through the MyGenome Application, you will help researchers learn more about how individuals understand and think about their genetic results. Sharing this information will give us access to your genome through the MyGenome Application, but is not required to participate in the survey portion of this research.

SHARE1. **Please indicate if you would be willing to share your clinical test report and your access code for the MyGenome Application by selecting ONE of the following two options...**

1 I authorize Illumina, Inc. to provide a copy of my clinical test report to the study team at Brigham and Women's Hospital.
0 I do NOT authorize Illumina, Inc. to provide a copy of my clinical test report to the study team at Brigham and Women's Hospital.

{PRG: SHOW SHARE2 IF SHARE1=1}

SHARE2: Thank you for your willingness to share your clinical report. To verify your permission, please enter your PG number below. Your PG number is located in the header of your clinical test report. *(If your report or your PG Number is not available, please continue to the next question and we will contact you separately for verification.)*

PG0000 [PGNUM: TEXT RESPONSE, REQUIRE 4 CHARACTERS] - [PGSUFFIX: POPLIST, OPTIONS: 1=BLD, 2=DNA]

{PRG: SHOW IF PRE_1=3; OTHERWISE SKIP TO DEM6}

Thank you for contributing to this research study.

We would like to be able to link your responses to your personal genome sequencing results report provided to you by the Mount Sinai study team.

By sharing your personal genome sequencing results report you will help researchers learn more about how individuals understand and think about their personal genomic information. Sharing this information is not required to participate in the survey portion of this research.

HEALTHSEQ_SHARE1. **Please indicate if you would be willing to have your research results report shared by selecting ONE of the following two options...**

1 I authorize Mount Sinai to provide a copy of my personal genome sequencing results report to the study team at Brigham and Women's Hospital.
0 I do NOT authorize Mount Sinai to provide a copy of my personal genome sequencing results report to the study team at Brigham and Women's Hospital.

{PRG: SHOW DEM8 INTRO AND DEM8 ON SAME PAGE}

DEM8 INTRO. We hope to complete a follow-up survey with you in approximately one year. To ensure that we can locate you then, we would like to verify your mailing address, so that we may send you a letter invitation.

The mailing address we currently have on file for you is:

[DISPLAY ADDRESS]

DEM8. Is the above address correct?

1 Yes, that is my current mailing address.
2 No, I would like to edit my mailing address.

{PRG: SUBMIT SURVEY B AS COMPLETE AND PASS TO SURVEY C}

{PRG: SOFT PROMPT ON DEM6 SCREEN – PROMPT IS SET TO TRIGGER IF DEM6A,C,D,F OR BOTH DEM7A & B ARE BLANK}

DEM6. Please provide us with your mailing address:

DEM6a. Street 1: [TEXT RESPONSE]
DEM6b. Street 2: [TEXT RESPONSE]
DEM6c. City: [TEXT RESPONSE]
DEM6d. State/Province: [TEXT RESPONSE]
DEM6f. Zip/Postal Code: [TEXT RESPONSE; PRG: LIMIT TO 10 CHARACTERS]
DEM6e. Country: [TEXT RESPONSE]

DEM7a. Cell Phone: [TEXT RESPONSE]
DEM7b. Home or Office Phone: [TEXT RESPONSE]

DEM6g. Other/International Address Information: [OPEN END RESPONSE]

We have recorded your email as {INSERT DEM2}. Please provide us with an alternative (i.e., home, school, work) that we may use in case we are not able to reach you at your primary email.

DEM7c. Alternative Email: [EMAIL ADDRESS]

PeopleSeq Consortium
Catch-up Survey

PEOPLESEQ PRE-POST DISCLOSURE (a.k.a., CATCH-UP) QUESTIONNAIRE SPECIFICATIONS MASTER

Short URL to direct towards production survey	https://www.ssgresearch.com/peopleseq
Support email address to include in header	peopleseq@ssgresearch.com
Support phone number to include in header (if needed)	

Logo to use if other than SSG logo
Please list network location of other logo to use: GDrive/S15002 PeopleSeq 2015/DEFLOG.gif

---

Mandatoriness (check the appropriate setting)

X All questions are optional unless otherwise noted
All questions are optional with a soft prompt included if no answer is provided
[OTHER SPECIFY FIELDS SHOULD ALSO HAVE SOFT PROMPTS]
All questions are mandatory

Please provide text to use for Mandatoriness prompt if being used (Default text to use is provided below):

GENERAL SOFT PROMPT:

We noticed that you did not answer a question on the previous page. It is important to us that we get a complete set of responses from you. Please return to the previous page by clicking "Previous" and select an answer for each question. If you would rather not select an answer, you may instead continue to the next page by clicking "Next."

OTHER SPECIFY SOFT PROMPT:

You selected 'Other' but did not specify your answer. Please return to the last question by clicking "Previous" and type in your specific answer. If you would rather not specify an answer, you may instead continue to the next page by clicking "Next."

Section Headers

Section Label	Section Start Page #
1. **About You**	5
2. **Your Decision**	12
3. **Your Genome Results Report: Information**	16
4. **Your Genome Results Report: Attitudes**	35
5. **Your Entire Genome Sequence**	39
6. **General Attitudes Towards Genomics**	41
7. **A Few More Questions About You**	42
8. **Wrap-Up**	54

Survey Title to Appear in Header (appears above the section header bar)

The Personal Genome Sequencing Outcomes Study

Welcome Page Text (modify the following as needed)

Welcome to the Personal Genome Sequencing Outcomes Study!

This is one of the first large-scale systematic studies to examine the experiences, attitudes, and outcomes of healthy adults who have chosen to pursue personal genome sequencing. Our hope is that the knowledge gained through this study will play an integral role in shaping the development of genome sequencing policy and its integration into health care.

This is the first survey for this study!

{PRG: IF AUTO-LOGIN PROTOCOL IS BEING USED, INSERT THE FOLLOWING TEXT}
Please click Start Survey to begin!

Resume Page Text (modify the following as needed)

Thank you for returning to the survey. Please click “resume” to begin where you last left off…

Terminate Text (modify the following as needed)

Thank you for considering participation in this study. Participation requires that you provide consent. We will not contact you again about this study. If you believe you received this message in error, please contact us at the email in the upper right corner of the screen so that we may reset your case.

End Page Text

Thank you for your participation! We will email you with a link to the next survey in a year’s time.

For information about the Personal Genome Sequencing Outcomes Study, including contact details, please refer to the study website at: http://www.genomes2people.org/the-peopleseq-study/ {PRG: Set link to open in new window.}

Results of the Personal Genome Sequencing Outcomes Study will be posted on the study website as they become available.

We really appreciate the time you have taken to complete this survey. You will now be entered into a sweepstakes drawing for an Apple Watch, and we will be in touch to let you know if you are the winner.

You may now close your browser.

Survey Title Appearing in Browser Window

Personal Genome Sequencing Outcomes Study

GENERAL PROGRAMMING NOTES

For confidentiality purposes, this questionnaire will be programmed as three separate surveys. Survey A will be the consent form, some basic contact information, and a few demographics. Survey B will be the main body of the questionnaire. And Survey C will be asking for additional contact information.

Please pass all demographics collected in Survey A into Survey B and store them in a calculation there – to facilitate data processing/review. Do NOT pass identifiable data, except for DATSTAT_ALTPID.

All emphasis should be programmed in black, all caps text instead of lowercase blue text.

Lines indicate page breaks throughout the survey and show which should questions appear together.

PRELOADS

PRE_1 Genome Sequencing Program

1 UYG
2 PGP
3 HealthSeq
4 CEO Genome Project
5 MD/PhD Genome Project
6 SOAPS
7 Pioneer 100
8. UVM UYG
9. Invitae
10 Hudson Alpha

{NOTE: PRE_0 IS A CALCULATION DRIVEN OFF OF PRE_1 - **IT SHOULD NOT BE PRELOADED}**
PRE_0 Genome Sequencing Program (with Acronym)

1 Understand Your Genome (UYG)
2 The Harvard Personal Genome Project (PGP)
3 the Whole Genome Sequencing in the Clinical Setting (HealthSeq) Study at Mount Sinai
4 CEO Genome Project
5 MD/PhD Genome Project
6 UNLV Survey of Opinions about Personalized-Medicine Services (SOAPS)
7 Pioneer 100
8. UVM UYG
9. Invitae
10 Hudson Alpha

{PRG: START SURVEY A}

CONSENT

{NOTE: Center first two lines on screen; NO scrolling text box for consent; add PDF link at top of screen (separate PDFs for each company)}

Welcome to the Personal Genome Sequencing Outcomes Study!

Please read this consent form, and then make one of the choices below.

INSERT CONSENT LANGUAGE HERE {PRG: Final Consent language coming after initial programming. Consent form will dynamically change depending on the value of PRE_1}

{NOTE: Add space between consent options}
{PRG: TIMESTAMP UPON QCONSENT SUBMISSION}
{PRG: QCONSENT REQUIRED}

QCONSENT. Please read the consent form above, and then select one of the choices below. Please enter your first and last name below as your signature confirming your selection, and then click "Next" to proceed.

1 I have read this consent form, and I agree to the study procedures described above. I confirm that I am 18 years or older. I confirm that I have signed up for personal genome sequencing through {DISPLAY PRE_0}, and I have received an email inviting me to participate in this research study.
2 I do NOT wish to participate in this study.

DEM1C: Title

2 Dr.
3 Ms.
4 Mrs.
5 Mr.
6 Miss
7 Mx.
8 Hon

{DEM1A & DEM1B ARE REQUIRED IF QCONSENT=1}

DEM1A: First Name

DEM1B: Last Name

{PRG: If QConsent=2, submit case as TERMINATE. DO NOT Send Thank You Email noted on next page}

{Note: Section Header 1: About You}

SECTION INTRO: *First, we would like to ask a few questions about you to help us analyze the results of the survey, and so that we can contact you again for future surveys. Please note that the first five questions are required in order to participate. We will only use your email address to contact you as part of this study; we will never use this information for any other purpose.*

{PRG: SAME SCREEN DEM2-DEM5}
{PRG: MAKE DEM2-DEM5 REQUIRED – ADD RED STAR TO INDICATE REQUIREMENT}
{PRG: VALIDATE EMAIL ADDRESSES CONTAINS @ and}

DEM2. What is your email address? *(Please enter in the format email@address.com)*

Email address: [EMAIL ADDRESS]

DEM3. What year were you born in?

[NUMERIC 1894-1997]

DEM4a. Which gender do you identify as?

1 Female
2 Male
3 Other *(Please specify)*: [TEXT RESPONSE]

DEM4b. What was your anatomical sex at birth?
1 Female
2 Male
3 Intersex or other *(Please specify):* [TEXT RESPONSE]
4 Choose not to answer

DEM5. Where do you currently live?

1 Within the United States
2 Outside the United States *(Please specify city and country)*: [TEXT RESPONSE]

Invitae Participant Note {PRG: DO NOT SHOW}
{PRG: SHOW ON ALL PAGES IF PRE_1=9 (INVITAE)}
Invitae Participants: In this survey, ***genome*** refers to ***genetic***, ***genome sequencing*** refers to ***genetic testing***, and ***genome reports*** refer to ***genetic test reports***.

FQ1. Have you received your personal genome sequencing results from {DISPLAY PRE_0}?
1 Yes
2 No

{PRG: SHOW FQ2 IF FQ1=1, OTHERWISE SKIP TO SURVEY B}

FQ2. When did you first receive your {RESPONSE:PRE_1} results (i.e., your personal genome sequencing results report)? *(If you are unsure, please provide your best estimate.)*

AD1_MONTH: Drop-Down: (1=Jan through 12=Dec)
AD1_YEAR: Drop-Down: (2000 through 2017)

{SUBMIT SURVEY A AND EMAIL ALERT} Send email to R thanking them for agreeing to participate and giving them a direct link to access the main survey instrument. This is to allow Rs to leave main survey and return later if desired. Include link to a copy of the consent form.

TEXT. *Next, we would like to ask a few more questions about you.*

Invitae Intro Note {PRG: DO NOT SHOW}
{PRG: SHOW ONLY IF PRE_1=9 (INVITAE)}

NOTE TO INVITAE PARTICIPANTS:

Some terminology used in the survey questions may differ from terms you and your health care professional have used when discussing your Invitae genetic testing and/or your test results. A note is provided at the top of each page of the survey that describes how different terms should be defined; please refer to this note if any terms in the questions are unfamiliar to you.

{PRG: K2 USE GENERAL SOFT PROMPT}

K2. What is your marital status?

1 Married
2 Widowed
3 Divorced
4 Separated
5 Never married

{PRG: SHOW K2A IF K2=2,3,4,5}

K2a. Are you currently living with a boyfriend/girlfriend or spouse/partner?

1 Yes
2 No

{PRG: K1 USE GENERAL SOFT PROMPT}

K1. Are you adopted?

1 Yes
0 No
2 Not sure

{PRG: K3 USE GENERAL SOFT PROMPT}

K3. How many children (including both biological and non-biological) do you have? *(Enter "0" if you have none.)*

[NUMERIC ENTRY 0-30]

{PRG: SHOW K3a IF K3>0, OTHERWISE SKIP TO K5}
{PRG: K3a USE GENERAL SOFT PROMPT}

K3a. How many biological children do you have? *(Enter "0" if you have none.)*

[NUMERIC ENTRY 0-30]

{PRG: SHOW K3b if K3a>0, OTHERWISE SKIP TO K5}
{PRG: K3b USE GENERAL SOFT PROMPT}

K3b. Are any of your biological children less than 18 years old?

1 Yes
0 No

{PRG: SHOW INVITAE_1 ONLY IF PRE_1=9 (INVITAE); OTHERWISE GO TO FILTERS BEFORE K5}

INVITAE_1. Including yourself, how many people are in your **nuclear family**? *A nuclear family is a partnered adult couple or a single adult and all dependent children who live together at the same address.*

[DISCRETE NUMERIC RESPONSE 1-100]

INVITAE_2. Including yourself, how many people are in your **extended family**? *An extended family includes a nuclear family plus grandparents, aunts, uncles and/or other relatives who live together in the same household.*

[DISCRETE NUMERIC RESPONSE 1-100]

INVITAE_3. Do most of your family members who do not live in your household, reside within one hour's drive of your home?

1 Yes
2 No
3 Not sure

INVITAE_4. Do you typically share your personal health information with any other individuals in your nuclear or extended family?

1 Yes
2 No

{PRG: IF INVITAE_4=1, INVITAE K3b_3a; OTHERWISE GO TO K5}

INVITAE_5. With whom do you typically share your personal health information? *(Select all that apply.)*

1 Spouse/partner
2 Children
3 Parents
4 Other relatives
5 Other (Specify): [TEXT RESPONSE]

{PRG: K5 - SELECT ALL THAT APPLY}
{PRG: K5 USE GENERAL SOFT PROMPT}
{PRG: K5.8.TEXT USE OTHER SPECIFY SOFT PROMPT}
{DESIGN: K5/K4 ON SAME PAGE}

K5. Which one or more of the following would you say is your race? (*Please select all that apply*.)

1 American Indian or Native Alaskan
2 Black or African American
3 East Asian
4 Hawaiian or other Pacific Islander
6 South Asian
7 White or Caucasian
8 Other *(Please specify):* [TEXT RESPONSE]

{PRG: K4 USE GENERAL SOFT PROMPT}

K4. Do you consider yourself Hispanic or Latina/o?

1 Yes
0 No

{PRG: SHOW QUESTION NOTE ON TOP OF PAGE IF PRE_1=9 (INVITAE)}
QUESTION NOTE: Due to the topic of the PeopleSeq study, this survey includes some questions that are of a sensitive nature. We greatly appreciate your open and honest responses to all questions - all data remains strictly confidential and anonymous; however, you are not required to respond to all questions.

K10. How religious do you consider yourself to be?

1 Not at all religious
2 Not very religious
3 Somewhat religious
4 Very religious

K11. Which of the following best describes your religion or worldview?

1 Agnostic
2 Atheist
3 Buddhist
4 Christian
5 Hindu
6 Jewish
7 Muslim
8 Protestant
9 Other *(Please specify):* [TEXT RESPONSE]

{PRG: K6 USE GENERAL SOFT PROMPT}

K6. What is the highest level of education you have completed?

1	Never attended school
2	Grade school (grades 1 to 8)
3	Some high school (grades 9 to 12)
4	High school graduate or GED
5	Post high school training other than college (vocational, technical, or other types of training)
6	Some college
7	Associate's degree
8	Bachelor's degree or equivalent
9	Some graduate school
10	Master's degree (MS, MBA, MFA, etc.)
11	Some doctoral work
12	Doctoral or other professional degree (MD, PhD, JD, or other)

{PRG: K7 USE GENERAL SOFT PROMPT}
{PRG: K7 SELECT ALL THAT APPLY}

K7. What is your current work situation? *(Please select all that apply.)*

1	Working full-time
2	Working part-time
3	Temporarily laid off, sick leave, or maternity leave
4	Retired
5	Self-employed
6	Looking for work, unemployed
7	Disabled, permanently or temporarily
8	Homemaker
9	Student

{PRG: K8 USE GENERAL SOFT PROMPT}
{PRG: K8.11.TEXT USE OTHER SPECIFY SOFT PROMPT}

K8. In which field do you now or did you most recently work? *(Please select all that apply.)*

1	Business, Financial, Management, Sales, and Related Occupations
2	Computer, Engineering, and Mathematical Science
3	Life, Physical, and Social Science
4	Legal
5	Education, Training, and Library
6	Arts, Design, Entertainment, Sports, and Media
7	Healthcare Provider or Clinical Researcher
8	Office and Administrative Support
9	Construction, Maintenance, and Natural Resources
10	Production and Transportation
11	Other *(Please specify):* [TEXT RESPONSE]

{PRG: SHOW IF K8=7; OTHERWISE GOTO K9}
{DESIGN: SHOW K8a & K8b ON SAME PAGE}
{PRG: K8a USE GENERAL SOFT PROMPT}
{PRG: K8a.9.TEXT USE OTHER SPECIFY SOFT PROMPT}

K8a. What is your educational background? *(Please select all that apply.)*

1 MD
2 PysD
3 PharmD
4 PhD
5 Master's in Genetic Counseling
6 Registered Nurse
7 Nurse Practitioner
8 Physician Assistant
9 Other *(Please specify):* [TEXT RESPONSE]

{PRG: K8b USE GENERAL SOFT PROMPT}
{PRG: K8b.11.TEXT USE OTHER SPECIFY SOFT PROMPT}

K8b. What is your primary clinical specialty?

1 Primary care
2 Genetics specialist (e.g., genetic counselor, clinical geneticist)
3 Anesthesiology
4 Nutrition
5 Obstetrics/gynecology
6 Oncology
7 Internal medicine
8 Surgery
9 Pediatrics
10 I do not have a specialty
11 Other specialty *(Please specify):* [TEXT RESPONSE]

K9. What is your household's total combined income during the past 12 months? *(This includes money from pensions, social security payments, jobs, net income from business, farm or rent, dividends, interest, and any other income received by family members who are 15 years of age or older. Options listed are in U.S. Dollars.)*

1 Less than $20,000
2 $20,000 – $39,999
3 $40,000 – $69,999
4 $70,000 – $99,999
5 $100,000 – $199,999
6 $200,000 – $500,000
7 More than $500,000

{PRG: OPTION 10 IS MUTUALLY EXCLUSIVE}

H1. Do you have health insurance or a health coverage plan? *(Please select all that apply.)*

1	Yes, through my employer
2	Yes, through someone else's employer
3	Yes, a plan that I or someone else buys
4	Yes, through Medicare
5	Yes, through Medicaid or Medical Assistance
6	Yes, through the military, CHAMPUS, or the VA
7	Yes, through the Indian Health Service or the Alaska Native Health Service
11	Yes, through other national or government sponsored health service
8	Yes, through some other source *(Please specify):* [TEXT RESPONSE]
9	No, I don't have any coverage
10	I don't know

H2-H4. Do you have…

1	Yes
0	No
99	I don't know

H2. Life insurance?
H3. Disability insurance?
H4. Long-term care insurance?

{PRG: SHOW H3a IF H3=1}

H3a. Is your disability insurance short term, long term, or both?

1	Short term
2	Long term
3	Both short and long term
99	I don't know

L1. Which of the following best applies to you? *(Please note cigarettes refer to tobacco and not electronic cigarettes.)*

1	I smoke cigarettes (including hand-rolled) every day.
2	I smoke cigarettes (including hand-rolled), but not every day.
3	I do not smoke cigarettes at all, but I do smoke tobacco of some kind (e.g., pipe or cigar).
4	I have stopped smoking completely in the last year.
5	I stopped smoking completely more than a year ago.
6	I have never been a smoker (i.e., smoked for a year or more).

{PRG: SHOW IF L1=1 OR 2, ELSE GOTO Q1}

L2. Are you seriously thinking of quitting smoking?

1	Yes, within the next 30 days
2	Yes, within the next 6 months
3	No, not thinking of quitting

{Note: Section Header 2: Your Decision}

SECTION INTRO: *In this section, we will be asking you some questions about the personal genome sequencing that you had done through {DISPLAY PRE_0}.*

Invitae Participant Note {PRG: DO NOT SHOW}
{PRG: SHOW ON ALL PAGES IF PRE_1=9 (INVITAE)}
Invitae Participants: In this survey, ***genome*** refers to ***genetic***, ***genome sequencing*** refers to ***genetic testing***, and ***genome reports*** refer to ***genetic test reports***.

First, please think about the process you went through when deciding whether or not to pursue personal genome sequencing through {DISPLAY PRE_1}.

Q1. How long did it take you to decide to pursue personal genome sequencing through {DISPLAY PRE_1}?

1 One day or less
2 More than one day but less than a week
3 Between one and two weeks
4 Several weeks (more than two weeks but less than a month)
5 Between one and two months
6 More than two months
7 Not sure

Q2. When making your decision, did you talk with anyone about whether or not to pursue personal genome sequencing through {DISPLAY PRE_1}?

1 Yes
0 No

{PRG: SHOW Q2a IF Q2=1; OTHERWISE SKIP TO Q6}
{PRG: SELECT ALL THAT APPLY}

Q2a. Whom did you talk to about whether or not to pursue personal genome sequencing through {DISPLAY PRE_1}? *(Please select all that apply.)*

1 Family members
2 Friends
3 Co-workers/colleagues
4 Primary care provider
5 Genetics specialist (e.g., genetic counselor or clinical geneticist)
6 Other healthcare provider(s)
7 Representative from [DISPLAY PRE_1]
8 Other *(Please specify)*: [TEXT RESPONSE]

{PRG: IF PRE_1=9 (INVITAE), THEN AUTOFILL MD1=1; DO NOT SHOW RESPONSE OPTIONS ON SCREEN AFTER PREFILL}

MD1. Were you required to have a physician order this personal genome sequencing through {DISPLAY PRE_1} for you?

1 Yes
2 No
3 I don't know

{PRG: SHOW MD2 IF MD1=1, OTHERWISE SKIP TO FILTER BEFORE B3b}

MD2. How willing was the physician to order this personal genome sequencing through {DISPLAY PRE_1} for you?

1 Not at all willing
2 Somewhat willing
3 Very willing

Comments: [OPEN RESPONSE]

{PRG: SHOW B3b IF Q2a=4 OR 5 OR 6, OTHERWISE SKIP TO Q6}
{PRG: IF PRE_1=9 (INVITAE), THEN AUTOFILL B3b=1; DO NOT SHOW RESPONSE OPTIONS ON SCREEN AFTER PREFILL }

B3b. Were you required to consult with a healthcare provider before you received your personal genome sequencing results from {DISPLAY PRE_1}?

1 Yes
2 No
3 I don't know

Q6. Did you pay for the personal genome sequencing you will receive through {DISPLAY PRE_1}?

1 Yes
2 No

{PRG: SHOW IF Q6=1; OTHERWISE GOTO B1}

Q6a. How much did you pay for the personal genome sequencing you will receive through {DISPLAY PRE_1}? *(Please estimate your genetic testing cost in in U.S. Dollars.)*

[NUMERIC RANGE 1-99999] U.S. Dollars

{DESIGN: GRID B1_1- B1_9}

B1. *People pursue personal genome sequencing for many different reasons. For each of the following statements listed here and on the next page, please select the response that is most appropriate for you.*

How important were the following factors in your decision to pursue personal genome sequencing through {DISPLAY PRE_1}?

1 Not at all important
2 Somewhat important
3 Very important
4 Not applicable

B1_1. Curiosity about my genetic make-up
B1_2. Interest in finding out about my personal disease risk
B1_3. Interest in finding out what I can do to improve my health
B1_4. {PRG: Do not show if PRE_1=4 OR 5} Interest in finding out about my personal response to medications
B1_5. Desire to plan for the future
B1_6. General interest in genetics
B1_7. It seemed like it would be fun and entertaining
B1_19. Desire to participate in research to help others
B1_8. {PRG: Show if PRE_1=2 OR 4} Desire to contribute my genome sequence data to research
B1_9. {PRG: Show if PRE_1=2} Desire to learn about my genetic make-up without going through a physician
B1_18. {PRG: Do not show if PRE_1=4 OR 5 **OR 9**} Interest in my ancestry

---

{DESIGN: GRID B1_10- B1_17}
{DESIGN: B1 & B1_OTH ON SAME PAGE}

B1. *People pursue personal genome sequencing for many different reasons. For each of the following statements, please select the response that is most appropriate for you.*

How important were the following factors in your decision to pursue personal genome sequencing through {DISPLAY PRE_1}?

1 Not at all important
2 Somewhat important
3 Very important
4 Not applicable

B1_10. Other members of my family have had their genomes sequenced
B1_11. To learn more about my genetics because I lack information about my family history
B1_12. To provide disease risk information for my children
B1_13. There is a medical condition in my family that may be genetic
B1_14. There is a medical condition in my family that has been confirmed to be genetic
B1_15. {PRG: Show if K1=1} To learn more about my genetics because I am adopted
B1_16. To learn more about genome sequencing as part of my professional activities
B1_17. To contribute to the advancement of science
B1_20. Interest in learning as much about myself as possible
B1_21. It seemed like a novel opportunity

B1_OTH. If any other factors were important in your decision of whether or not to pursue personal genome sequencing through {DISPLAY PRE_1}, please specify here.

[TEXT RESPONSE] {300 CHARACTER LIMIT}

{PRG: HIDE ANY OPTION TO WHICH THE CORRESPONDING B1_ ROW = 1 or 4}

B1_MOST. Which factor was the **MOST** important in your decision to pursue personal genome sequencing through {DISPLAY PRE_1}?

1. Curiosity about my genetic make-up
2. Interest in finding out about my personal disease risk
3. Interest in finding out what I can do to improve my health
4. Interest in finding out about my personal response to medications
5. Desire to plan for the future
6. General interest in genetics
7. It seemed like it would be fun and entertaining
19. Desire to participate in research to help others
8. {PRG: Show if PRE_1=2} Desire to contribute my genome sequence data to research
9. {PRG: Show if PRE_1=2} Desire to learn about my genetic make-up without going through a physician
18. Interest in my ancestry
10. Other members of my family have had their genomes sequenced
11. To learn more about my genetics because I lack information about my family health history
12. To provide disease risk information for my children
13. There is a medical condition in my family that may be genetic
14. There is a medical condition in my family that has been confirmed to be genetic
15. {PRG: Show if K1=1} To learn more about my genetics because I am adopted
16. To learn more about genome sequencing as part of my professional activities
17. To contribute to the advancement of science
20. Interest in learning as much about myself as possible
21. It seemed like a novel opportunity
98. [TEXT RESPONSE FROM B1_OTH]

{PRG: SHOW IF B1_14=2 OR 3}

B1_14a. What confirmed genetic condition(s) do you have in your family?

[MEMO RESPONSE]

{DESIGN: GRID B2_1-B2_7}
{DESIGN: SAME SCREEN B2-B2_OTH}

B2. *People have different concerns about personal genome sequencing. For each of the following statements, please select the response that is most appropriate for you.*

How concerned were you about each of the following factors when you were deciding whether or not to pursue personal genome sequencing through {DISPLAY PRE_1}?

1 Not at all concerned
2 Somewhat concerned
3 Very concerned

B2_1. How well the results would predict my future disease risk
B2_2. The privacy of my genetic information
B2_4. The possibility that I might receive unwanted information
B2_5. The financial cost of having my genome sequenced
B2_6. The complexity of genetic variant interpretation
B2_7. The impact the results might have on my ability to obtain insurance

B2_OTH. If you were concerned about any other factors when deciding whether or not to pursue personal genome sequencing, please specify here.

[OPEN END RESPONSE]

{DESIGN: GRID F7a-F7e}

F7. *Please reflect on the decision that you made to pursue personal genome sequencing.*

To what extent do you agree or disagree with the following statements?

1 Strongly disagree
2 Somewhat disagree
3 Neither agree nor disagree
4 Somewhat agree
5 Strongly agree

F7a. It was the right decision.
F7b. I regret the decision that I made.
F7c. I would make the same decision again.
F7d. The decision did me a lot of harm.
F7e. The decision was a wise one.

F3. In general, how satisfied are you with your decision to obtain personal genome sequencing through {DISPLAY PRE_1}?

1 Not at all satisfied
2 Not very satisfied
3 Somewhat satisfied
4 Very satisfied

{Note: Section Header 3: Your Genome Results Report: Information}

Invitae Participant Note {PRG: DO NOT SHOW}
{PRG: SHOW ON ALL PAGES IF PRE_1=9 (INVITAE)}
Invitae Participants: In this survey, ***genome*** refers to ***genetic***, ***genome sequencing*** refers to ***genetic testing***, and ***genome reports*** refer to ***genetic test reports***.

TEXT. *Next, we'd like to ask you some questions about the personal genome sequencing results you received through {DISPLAY PRE_0}.*

AD1. When did you first receive your {RESPONSE:PRE_1} results (i.e., your personal genome sequencing results report)? *(If you are unsure, please provide your best estimate.)*

AD1_MONTH: Drop-Down: (1=Jan through 12=Dec)
AD1_YEAR: Drop-Down: (2000 through 2017)

F1. How many times have you viewed your {DISPLAY PRE_1} results since receiving them?

0 Never
1 1 time
2 2-3 times
3 4 or more times

{SHOW F2 IF F1 > 0, OTHERWISE GO TO FILTER BEFORE DC1}

F2. In total, how much time have you spent reviewing your {DISPLAY PRE_1} results?

1 Less than 1 hour
2 1-2 hours
3 2-5 hours
4 5-10 hours
5 More than 10 hours

{PRG: SHOW IF PRE_1=4 or 5; OTHERWISE GO TO C0}

As science advances, researchers are able to learn additional information about the link between genetic variants and diseases and health conditions. Using this new information, it is possible for geneticists to create updated personal genome reports by comparing this newly discovered information to your genomic information.

DC1. Did you receive an updated personal genome report from Dr. Caskey?

1 Yes
2 No

{DESIGN: GRID DC2_1-DC2_4}
{PRG: SHOW IF DC1=1, OTHERWISE GO TO FILTER BEFORE DC3}

DC2. In general, how useful did you feel this updated personal genome report was to you?

1 Not at all useful
2 Not very useful
3 Somewhat useful
4 Very useful

Please explain why you feel this updated personal genome report was or was not useful.

[OPEN ENDED RESPONSE]

{DESIGN: GRID DC3_1-DC3_4}
{PRG: SHOW IF DC1=2, OTHERWISE SKIP TO DC4}

DC3. How interested would you be in receiving an updated personal genome report?

1 Not at all interested
2 Not very interested
3 Somewhat interested
4 Very interested

DC4. If you had to pay for updated personal genome reports, how much would you be willing to pay for an updated report?

[TEXT RESPONSE]: __________ US Dollars

DC5. How often would you be interested in receiving an updated personal genome report if there were no cost to you?

1 Never
2 Every 6 months
3 Every year
4 Every 2 years
5 Every 5 years or more

{DESIGN: GRID C0}
{PRG: SHOW C0_8 IF PRE_1=3}
{PRG: IF PRE_1=9 (INVITAE), THEN AUTOFILL CO_3=2 AND CO_4=2 AND CO_8=2; DO NOT SHOW RESPONSE OPTIONS ON SCREEN AFTER PREFILL}

C0. Please indicate whether you ***were offered*** each of the following types of results as part of your personal genome sequencing report (i.e., your {RESPONSE:PRE_1} results).

1 Yes
2 No

C0_1. Information about my risk of a disease or diseases
C0_2. How my body responds to drugs or medications
C0_3. My carrier status
C0_4. Information about my ancestry
C0_5. Information explaining the cause of a disease I already knew I had
C0_6. My *APOE* genotype
C0_8. Physical traits (e.g. bitter tasting type)
C0_7. Other

{PRG: SHOW C0_OTH IF C0.7=1; OTHERWISE SKIP TO C13}

C0_OTH. What other types of results were you offered as part of your personal genome sequencing report?

[TEXT RESPONSE] {300 CHARACTER LIMIT}

{PRG: DO NOT SHOW IF PRE_1=4 OR 5}
{PRG: ASK C13 GRID IF ANY OF C0 IS SELECTED, AND PRESENT ONLY CORRESPONDING ROWS WHERE C0=1}
{PRG: IF PRE_1=9 (INVITAE), THEN AUTOFILL C13_3=2 AND C13_4=2 AND C13_7=2; DO NOT SHOW RESPONSE OPTIONS ON SCREEN AFTER PREFILL}

C13. Please indicate whether you ***opted to receive*** each of the following types of results as part of your personal genome sequencing report (i.e., your {RESPONSE:PRE_1} results).

1 Yes
2 No

C13_1. Information about my risk of a disease or diseases
C13_2. How my body responds to drugs or medications
C13_3. My carrier status
C13_4. Information about my ancestry
C13_5. Information explaining the cause of a disease I already knew I had
C13_6. My *APOE* genotype
C13_8. Physical traits (e.g. bitter tasting type)
C13_7. [TEXT RESPONSE FROM C0_OTH]

{PRG: SHOW IF C13_6=1; OTHERWISE SKIP TO FILTER BEFORE C1A}
{NOTE: EPSILON SYMBOL – HTML CODE **ε**}

C3a. What was your reported *APOE* genotype?

1 *APOE*-ε2/ε2
2 *APOE*-ε2/ε3
3 *APOE*-ε3/ε3
4 *APOE*-ε2/ε4
5 *APOE*-ε3/ε4
6 *APOE*-ε4/ε4
7 Genotype was not confidently reported
8 I prefer not to answer
9 I can't remember

{PRG: ASK C1A GRID IF ANY OF C13=1, AND PRESENT ONLY CORRESPONDING ROWS WHERE C13=1}

C1a. How interesting did you find each type of result that you received in your personal genome sequencing report through {DISPLAY PRE_1}?

1 Not at all interesting
2 Somewhat interesting
3 Very interesting

C1a_1. Information about my risk of a disease or diseases
C1a_2. How my body responds to drugs or medications
C1a_3. My carrier status
C1a_4. Information about my ancestry
C1a_5. Information explaining the cause of a disease I already knew I had
C1a_6. My *APOE* genotype
C1a_8. Physical traits (e.g. bitter tasting type)
C1a_7. [OTHER TEXT RESPONSE]

C2A_YN. Did you receive any personal information through {RESPONSE:PRE_1} that you felt was important to you?

1 Yes
2 No
3 Not sure

{PRG: ASK C2A_GRID IF C2A_YN=1}
{PRG: SOFT PROMPT IF NONE OF THE BELOW WERE ANSWERED}

C2A_GRID. Please list between one and three of the most important pieces of personal information that you received through {RESPONSE:PRE_1}.

C2a_GRID1 [TEXT RESPONSE]
C2a_GRID2 [TEXT RESPONSE]
C2a_GRID3 [TEXT RESPONSE]

{PRG: Repeat C2A_1-C2A_6 for each response provided. If no responses provided, then skip to A1}

The following questions relate to [RESULT 1, RESULT 2, RESULT 3].

C2A[1|2|3]_1. Is this information related to a condition that you have or may develop in the future?

1 Yes
2 No

{PRG: SHOW IF C2A[1|2|3]_1=1; OTHERWISE TERMINATE THIS LOOP}

C2A[1|2|3]_2. Have you previously received a diagnosis related to this condition?

1 Yes
2 No

{PRG: SHOW IF C2A[1|2|3]_2=1; OTHERWISE SKIP TO C2A_4}

C2A[1|2|3]_3. How was this diagnosis made?

1 Based on my {RESPONSE:PRE_1} results alone
2 Based on my {RESPONSE:PRE_1} results in combination with my medical/family history
3 Based on my {RESPONSE:PRE_1} results in combination with medical tests/procedures that were performed because of my {RESPONSE:PRE_1} results
4 Other *(Please specify):* [TEXT RESPONSE]

C2A[1|2|3]_4. What do you think your chance is of developing this condition on a scale from 0-100%?

[NUMERIC RESPONSE 0-100]%

C2A[1|2|3]_5. Compared to the average person, what do you think your chance is of developing a condition related to this result?

1 Much higher
2 Somewhat higher
3 The same
4 Somewhat lower
5 Much lower

C2A[1|2|3]_6. How concerned are you about developing a condition related to this result?

1 Extremely concerned
2 Very concerned
3 Moderately concerned
4 Slightly concerned
5 Not at all concerned

A1. Have you discussed your {DISPLAY PRE_1} results with anyone?

1 Yes
0 No

{PRG: SHOW A1_A IF A1 = 0 OTHERWISE GOTO FILTER BEFORE A2}

A1_A. Why have you **NOT** discussed your [DISPLAY PRE_1] results with anyone?

[OPEN ENDED RESPONSE]

{PRG: SHOW IF A1=1; OTHERWISE SKIP TO A3_A}

A2. With whom did you discuss your {DISPLAY PRE_1} results? *(Please select all that apply.)*

1 Family members
2 Friends
3 Co-workers/colleagues
4 Healthcare provider {PRG: IF PRE_1=4 OR 5, DISPLAY "(other than Dr. Caskey)"}
5 Contacts on social networking services (e.g., Facebook, Twitter)
6 Contacts on health- or disease-based social networking services (e.g., {PRG: IF PRE_1=1, DISPLAY "UYG Community,"} PatientsLikeMe, Cure Together, disease-specific patient networks)
7 Other *(Please specify):* [TEXT RESPONSE]

{PRG: SHOW A2_A IF A2 DOES NOT EQUAL 1, OTHERWISE GOTO A2_1A}

A2_A. Why have you **NOT** discussed your [DISPLAY PRE_1] results with family members? *(Please select all that apply.)*

1 I don't feel that my results are important enough to share with my family members.
2 I don't think family members are interested in my results.
3 I am concerned about how my family members might react to my results.
4 I plan to discuss my results with family members but haven't gotten around to it yet.
5 Other *(Please specify):* [TEXT RESPONSE]

{PRG: SHOW IF A2=1; OTHERWISE SKIP TO A3_A}
{PRG: SELECT ALL THAT APPLY}

A2_1a. With which family member(s) did you talk about your {DISPLAY PRE_1} results? *(Please select all that apply.)*

1 Spouse/partner
2 Children
3 Brothers or sisters
4 Parents
5 Other relatives *(Please specify):* [TEXT RESPONSE]

{PRG: SHOW IF A2 DOES NOT=4 OR A1=0; OTHERWISE GO TO FILTER BEFORE A3_C}

A3_a. Why have you **NOT** discussed your [DISPLAY PRE_1] results with a healthcare provider {PRG: IF PRE_1=4 OR 5, DISPLAY "(other than Dr. Caskey)"}? *(Please select all that apply.)*

1 I have concerns about my genome sequencing results being placed in my medical record.
2 I don't feel that my results are important enough to share with a healthcare provider.
3 I plan to discuss my results with a healthcare provider but haven't gotten around to it yet.
4 I am confident in my ability to understand my results without the aid of a healthcare provider.
5 I do not think my healthcare provider is knowledgeable enough about genomic sequencing to use my results in my medical care.
6 I did not receive any results that required medical follow-up.
7 Other *(Please specify):* [TEXT RESPONSE]

{PRG: SHOW IF A2=4; OTHERWISE GO TO A7}

A3_c. With what type of healthcare provider(s) did you consult about your [DISPLAY PRE_1] results? *(Please select all that apply.)*

1 Primary care provider
2 Genetics specialist (e.g., genetic counselor or clinical geneticist)
3 Gastroenterologist
4 Anesthesiologist
5 Nutritionist
6 Obstetrician/gynecologist
7 Oncologist
8 Ophthalmologist
9 Physician assistant, nurse, or medical assistant
10 Reproductive endocrinologist
11 Surgeon
12 Pediatrician/child's physician
13 Other specialist/healthcare provider *(Please specify):* [TEXT RESPONSE]

{DESIGN: A3_D & A3_E ON SAME PAGE}
A3_d. In what way(s) did you consult with your healthcare provider(s) about your [DISPLAY PRE_1} results? *(Please select all that apply.)*

1 Office visit
2 Telephone
3 Email
4 Other *(Please specify):* [TEXT RESPONSE]

A3_e. How much time did you spend with your healthcare provider(s) discussing your results in total?

1 Less than 30 minutes
2 Between 30 minutes and 1 hour
3 Between 1 and 2 hours
4 More than 2 hours

{DESIGN: GRID A3_F-A3_G}

How willing was/were your healthcare provider(s) to…

1 Not at all willing
2 Somewhat willing
3 Very willing

A3_f. …discuss the meaning of your {DISPLAY PRE_1} results?
A3_g. …use your [DISPLAY PRE_1] results in your medical care?

A3_OE. Comments:

[OPEN ENDED RESPONSE]

A3_h. Did the interpretation(s) of your results provided by your healthcare provider(s) differ from the interpretation provided by [DISPLAY PRE_1]?

1 Yes
0 No

{PRG: SHOW A3_HOPEN IF A3_H=1}

A3_HOPEN. How did the interpretations differ?

[OPEN RESPONSE]

{DESIGN: SHOW A3_I AND A3_J ON SAME PAGE}

A3_i. How satisfied were you with your discussion(s) of your {DISPLAY PRE_1} results with your healthcare provider(s)?

1 Not at all satisfied
2 Not very satisfied
3 Somewhat satisfied
4 Very satisfied

A3_j. Please explain why you were or were not satisfied with your discussion(s) of your [DISPLAY PRE_1] results with your healthcare provider(s).

[OPEN ENDED RESPONSE]

A7. Have your {DISPLAY PRE_1} results prompted you to seek out more information about health or medical topics related to your results?

1 Yes
0 No

{PRG: SAME SCREEN A7A-A7B}
{PRG: SHOW A7A-A7B IF A7=1; OTHERWISE SKIP TO A8}

A7a. What types of information have your {DISPLAY PRE_1} results prompted you to seek out?

[OPEN END RESPONSE]

A7b. Where did you get the information?

[OPEN END RESPONSE]

A8. Have your {DISPLAY PRE_1} results prompted you to **make an appointment** with any healthcare provider(s)?

2 Yes, I have already made an appointment.
1 I plan to make an appointment.
0 No, I do not plan to make an appointment.

{PRG: SHOW IF A8=1 OR 2; OTHERWISE SKIP TO FILTER BEFORE A9A}

A8a. What type of healthcare provider(s) have your {DISPLAY PRE_1} results prompted you to make an appointment with? *(Please select all that apply.)*

1 Primary care provider
2 Genetics specialist (e.g., genetic counselor, clinical geneticist)
3 Gastroenterologist
4 Anesthesiologist
5 Nutritionist
6 Obstetrician/gynecologist
7 Oncologist
8 Ophthalmologist
9 Physician assistant, nurse, or medical assistant
10 Reproductive endocrinologist
11 Surgeon
12 Pediatrician/child's physician
13 Other specialist/healthcare provider *(Please specify):* [TEXT RESPONSE]

A8b. What was it about your [DISPLAY PRE_1] results that prompted you to make an appointment with any healthcare provider(s)? *(Please select all that apply.)*

1 There were results that I did not understand.
2 There was a result that required medical follow-up.
3 I wanted my results placed in my medical records.
4 Other *(Please specify):* [TEXT RESPONSE]

{PRG: SHOW A9A-A9B IF A2=4, OTHERWISE SKIP TO B1}
{PRG: SAME SCREEN A9A – A9B}

A9a. Which healthcare provider did you discuss your {DISPLAY PRE_1} results with the most?

[OPEN END RESPONSE]

Thinking about this healthcare provider with whom you discussed your {DISPLAY PRE_1} results the most, please indicate how much you agree or disagree with the following statement.

A9b. I believe that this healthcare provider understands genetics well enough to advise me on the implications of my {DISPLAY PRE_1} results for my health.

1 Strongly disagree
2 Somewhat disagree
3 Neither agree nor disagree
4 Somewhat agree
5 Strongly agree

B1. As a result of seeing your {DISPLAY PRE_1} results, have you spoken with a healthcare provider about any other tests, medical exams, or procedures?

1 Yes
0 No

{PRG: SHOW B1A IF B1=1, OTHERWISE GO TO B2}

B1a. What kind of tests, medical exams, or procedures have you spoken with a healthcare provider about as a result of seeing your {DISPLAY PRE_1} results? *(Please select all that apply.)*

1 One or more genetic tests to confirm a DNA sequence variant
2 One or more medical exams or procedures (other than genetic testing) to screen or test for a specific disease/condition
3 A whole body scan (i.e., an MRI in which images are taken of the whole body, not just one part)
4 Other *(Please specify):* [TEXT RESPONSE]

{PRG: SHOW B1B IF B1A = 1, OTHERWISE GO TO FILTER BEFORE B1C}

B1B. Please specify the genetic test(s) to confirm a DNA sequence variant that you spoke with a healthcare provider about as a result of seeing your {RESPONSE:PRE_1} results.

[MEMO RESPONSE]

{PRG: SHOW B1C IF B1A = 2, OTHERWISE GO TO B2}

B1C. Please specify the medical exams or procedures you spoke with a healthcare provider about as a result of seeing your {RESPONSE:PRE_1} results.

[MEMO RESPONSE]

B2. As a result of seeing your {DISPLAY PRE_1} results, have you **had** any tests, medical exams, or procedures?

1 Yes
0 No

{PRG: SHOW B2a IF B2=1, OTHERWISE GO TO C1}

B2a. What kind of tests, medical exams, or procedures have you **had** as a result of seeing your {DISPLAY PRE_1} results? (*Please select all that apply.)*

1 One or more genetic tests to confirm a DNA sequence variant
2 One or more medical exams or procedures (other than genetic testing) to screen or test for a specific disease/condition
3 A whole body scan (i.e., an MRI in which images are taken of the whole body, not just one part)
4 Other *(Please specify):* [TEXT RESPONSE]

{PRG: SHOW B2D & B2C IF B2A = 1, OTHERWISE GO TO FILTER BEFORE B2e}

B2d. Please specify the genetic test(s) to confirm a DNA sequence variant that you had as a result of seeing your {RESPONSE:PRE_1} results.

[MEMO RESPONSE]

B2c. Were the results of your genetic test(s) to confirm a DNA sequence variant consistent or inconsistent with your [DISPLAY PRE_1] results?

1 Consistent
2 Inconsistent
3 Results are still pending

{PRG: SHOW B2E IF B2A = 2, OTHERWISE GO TO C1}

B2e. Please specify the medical exams or procedures that you had as a result of seeing your {RESPONSE:PRE_1} results.

[MEMO RESPONSE]

TEXT: *Now we would like to ask you a few questions about your results and various health screens.*

C1. Blood cholesterol is a fatty substance found in the blood. Blood can be taken and used to determine your cholesterol level.

Because of your {DISPLAY PRE_1} results, have you had a blood test to check your cholesterol?

1 Yes
0 No
99 I don't know

C2. A blood glucose test is a blood test that measures the amount of sugar in your blood.

Because of your {DISPLAY PRE_1} results, have you had your blood sugar tested?

1 Yes
0 No
99 I don't know

C3_a. A colonoscopy/sigmoidoscopy is when a tube is inserted in the rectum to view the bowel for signs of cancer or other health problems. In this exam, all or part of the colon is checked. Anesthesia or pain medication is usually required.

Because of your {DISPLAY PRE_1} results, have you had a colonoscopy or sigmoidoscopy?

1 Yes
0 No
99 I don't know

C3_b. A fecal occult blood test (FOBT) or fecal immunochemical test (FIT) are two other tests used to screen for signs of colorectal cancer. For these tests, a stool sample is collected and checked for the presence of blood. There is no invasive testing involved, and no anesthesia or pain medications are required.

Because of your [DISPLAY PRE_1] results, have you had a FOBT or FIT test?

1 Yes
0 No
99 I don't know

C4. Because of your {DISPLAY PRE_1} results, have you had any tests in which a physician or healthcare provider looked for signs of heart disease, for example, an EKG (electrocardiogram) or a stress test?

1 Yes
0 No
99 I don't know

{PRG: SHOW C5-C7 IF DEM4b=1 OR 3 OR 4 ; OTHERWISE SKIP TO FILTER BEFORE C8}

C5. A mammogram is an x-ray of each breast to look for early signs of breast cancer.

Because of your {DISPLAY PRE_1} results, have you had a mammogram?

1 Yes
0 No
99 I don't know
2 {SHOW if DEM4b=3 or 4} N/A – Not applicable

C5a. MRI scans use magnets and radio waves instead of x-rays to produce very detailed, cross-sectional images of the body. MRI scans can take a long time-often up to an hour. You have to lie inside a narrow tube. For breast imaging, doctors inject a dye into a small vein in the arm before or during the exam.

Because of your {DISPLAY PRE_1} results, have you had a breast MRI scan?

1 Yes
0 No
99 I don't know
2 {SHOW if DEM4b=3 or 4} N/A – Not applicable

C6. A clinical breast exam is when a physician, nurse, or other healthcare provider feels your breasts for lumps.

Because of your {DISPLAY PRE_1} results, have you had a clinical breast exam?

1	Yes
0	No
99	I don't know
2	{SHOW if DEM4b=3 or 4} N/A – Not applicable

C7. A Pap test, also called a Pap smear, is when a physician or other healthcare provider uses a special stick or brush to take a few cells from inside and around the cervix.

Because of your {DISPLAY PRE_1} results, have you had a Pap test?

1	Yes
0	No
99	I don't know
2	{SHOW if DEM4b=3 or 4} N/A – Not applicable

{PRG: SHOW IF DEM4b=2 OR 3 OR 4; OTHERWISE SKIP TO C12}

C8. A Prostate-Specific Antigen test, also called a PSA test, is a blood test used to check men for prostate cancer.

Because of your {DISPLAY PRE_1} results, have you had a PSA test?

1	Yes
0	No
99	I don't know
2	{SHOW if DEM4b=3 or 4} N/A – Not applicable

C8a. A digital rectal exam (DRE) is when a physician or other healthcare provider inserts a gloved finger into the rectum to examine the prostate for irregularities in size, shape, or texture.

Because of your [DISPLAY PRE_1] results, have you had a DRE?

1	Yes
0	No
99	I don't know
2	{SHOW if DEM4b=3 or 4} N/A – Not applicable

C12. Because of your [DISPLAY PRE_1] results, have you had skin cancer (melanoma) screening (e.g., a physical examination of your skin to check for signs of cancer)?

1	Yes
0	No
99	I don't know

C9. Because of your {DISPLAY PRE_1} results, have you had any blood tests, imaging studies, or exams in which a physician or healthcare provider looked for signs of cancer, **other than those already listed above**?

1 Yes
0 No
99 I don't know

{PRG: SHOW C9a IF C9 = 1 OTHERWISE GO TO C11}

C9a. What other cancer screening tests/exams have you had because of your [DISPLAY PRE_1] results?

[MEMO RESPONSE]

C11. In total, how many medical visits with a physician or other healthcare provider {PRG: IF PRE_1=4 OR 5, DISPLAY "(besides Dr. Caskey)"} have you had as a direct result of receiving your {RESPONSE:PRE_1} results (in other words, visits you would <u>not</u> have had if you had <u>not</u> had personal genome sequencing)? *(If you are unsure, please provide your best estimate.)*

[NUMERIC RANGE 0-50] visits

G1_1a. Because of your {DISPLAY PRE_1} results, have you made any changes to your **diet**?

1 Yes
2 No
3 I don't know

{DESIGN: G1_1Y AND GRID G1_1B-G1_1D ON SAME PAGE}
{PRG: SHOW G1_1Y AND GRID G1_1B-G1_1D IF G1_1A=1, OTHERWISE SKIP TO G1_2A}

G1_1Y. What changes have you made to your **diet** because of your {DISPLAY PRE_1} results?

1 I have been eating more healthily.
2 I have been eating less healthily.
3 Other *(Please specify):* [TEXT RESPONSE]

G1_1. Was this change to your **diet**…

1 Yes
0 No

G1_1b. Self-motivated?
G1_1c. Recommended by a healthcare provider?
G1_1d. Recommended by [DISPLAY PRE_1]?

G1_2a. Because of your {DISPLAY PRE_1} results, have you made any changes to your **exercise routine or physical activity levels**?

1 Yes
2 No
3 I don't know

{DESIGN: G1_2Y AND GRID G1_2B-G1_2E ON SAME PAGE}
{PRG: SHOW G1_2Y AND GRID G1_2B-G1_2E IF G1_2A=1}

G1_2Y. What changes have you made to your **exercise routine or physical activity levels** because of your {DISPLAY PRE_1} results?

1 I have increased the amount I exercise.
2 I have decreased the amount I exercise.
3 Other *(Please specify):* [TEXT RESPONSE]

G1_2. Was this change to your **exercise routine or physical activity levels**…

1 Yes
0 No

G1_2b. Self-motivated?
G1_2c. Recommended by a healthcare provider?
G1_2d. Recommended by [DISPLAY PRE_1]?

G4. Because of your {DISPLAY PRE_1} results, have you made any changes to your **use of medications (including prescription and non-prescription) and/or supplements**?

1 Yes
2 No
3 I don't know

{PRG: SHOW G3 SERIES IF G4=1, OTHERWISE SKIP TO G2}

G3. How many changes have you made to any of your **medications (including prescription and non-prescription) and/or supplements** as a result of seeing your {DISPLAY PRE_1} results?

[NUMERIC RESPONSE 1-50] changes

{DESIGN: GRID G3a_1-G3a_3}
{PRG: FOR 0 < G3 <3, SHOW CORRESPONDING AMOUNT OF ROWS, IF G3 >3, ONLY SHOW 3 ROWS}

G3a. Please list the {PRG: IF G3>3, DISPLAY, "**three** most important"} medications/supplements that you made changes to.

G3a_1. [TEXT RESPONSE]
G3a_2. [TEXT RESPONSE]
G3a_3. [TEXT RESPONSE]

{DESIGN: G3B_1 –G3B_3}
{PRG: ONLY DISPLAY ROWS BELOW WHICH WERE ANSWERED IN G3A}

G3b. What type of medication/supplement is this?

1 Prescription medication
2 Non-prescription medication
3 Nutritional supplement
4 Alternative medicine
5 Other

G3b_1. {RESPONSE: G3a_1}
G3b_2. {RESPONSE: G3a_2}
G3b_3. {RESPONSE: G3a_3}

{DESIGN: G3C_1 –G3C_3}
{PRG: ONLY DISPLAY ROWS BELOW WHICH WERE ANSWERED IN G3A}

G3c. What changes did you make to this medication/supplement?

1 Stopped taking this medication/supplement
2 Lowered the dosage of this medication/supplement
3 Increased the dosage of this medication/supplement
4 Switched from this medication/supplement to another medication/supplement
5 Other

G3c_1. {RESPONSE: G3a_1}
G3c_2. {RESPONSE: G3a_2}
G3c_3. {RESPONSE: G3a_3}

{DESIGN: G3d_1 –G3d_3}
{PRG: ONLY DISPLAY ROWS BELOW WHICH WERE ANSWERED IN G3a}

G3d. Did you consult with a healthcare provider prior to making changes to these medications/supplements?

1 Yes
0 No

G3d_1. {RESPONSE: G3a_1}
G3d_2. {RESPONSE: G3a_2}
G3d_3. {RESPONSE: G3a_3}

{PRG: INCLUDE G3E_INTRO – G3E_3 ON SAME PAGE.}
{PRG: ONLY DISPLAY ROWS BELOW WHICH WERE ANSWERED IN G3A}

G3e intro. We would like to know a bit more about why your {DISPLAY PRE_1} results prompted you to make this change to this medication/supplement. Please write any further information about this in the box below.

G3e_1. {RESPONSE: G3a_1} [MEMO]
G3e_2. {RESPONSE: G3a_2} [MEMO]
G3e_3. {RESPONSE: G3a_3} [MEMO]

G2. Do you think you will use your {DISPLAY PRE_1} results to guide your **future** use of medications/supplements?

1 Yes
0 No
99 I don't know

{PRG: SHOW THE FOLLOWING QUESTION IF L1=4 OR 5}

L1A. Earlier in the survey, you indicated that you used to smoke but that you quit smoking a while ago.

Was your decision to quit smoking because of your {DISPLAY PRE_1} results?

1 Yes
2 No, I quit smoking before I received my {DISPLAY PRE_1} results.
3 No, I quit smoking after I received my {DISPLAY PRE_1} results, but my decision to quit smoking was not motivated by my {DISPLAY PRE_1} results.
4 I don't know

{PRG: SHOW IF L1A=1; OTHERWISE SKIP TO FILTER BEFORE L2A}
{DESIGN: GRID L1B_A-L1B_D}

L1B. Was your decision to quit smoking…

1 Yes
0 No

L1B_a. Self-motivated?
L1B_b. Recommended by a healthcare provider?
L1B_c. Recommended by [DISPLAY PRE_1]?

{PRG: SHOW IF L2=1 OR 2}

L2a. Earlier in the survey, you indicated that you are planning to quit smoking.

Is your plan to quit smoking because of your {DISPLAY PRE_1} results?

1 Yes
2 No
3 I don't know

{PRG: SHOW L2B IF L2A=1; OTHERWISE SKIP TO G1_3A}
{DESIGN: GRID L2B_a-L2B_d}

L2B. Is your plan to quit smoking…

1 Yes
0 No

L2B_a. Self-motivated?
L2B_b. Recommended by a healthcare provider?
L2B_c. Recommended by [DISPLAY PRE_1]?

G1_3a. Because of your {DISPLAY PRE_1} results, have you made changes to **any other aspects of your lifestyle or health behavior**, other than those already mentioned above?

1 Yes
2 No
3 I don't know

{DESIGN: G1_3Y AND GRID G1_3B-G1_3E ON SAME PAGE}
{PRG: SHOW G1_3Y AND GRID G1_3B-G1_3E IF G1_3A=1}
{DESIGN: GRID G1_3B-G1_3E}

G1_3Y. What **other aspects of your lifestyle or health behavior** have you changed because of your {DISPLAY PRE_1} results?

[TEXT RESPONSE]

G1_3. Was this change…

1 Yes
0 No

G1_3b. Self-motivated?
G1_3c. Recommended by a healthcare provider?
G1_3d. Recommended by [DISPLAY PRE_1]?

{PRG: IF H1= 9 OR 10, AND ALL H2-H4=0 OR 99, JUMP TO M2}

M1. Because of your {DISPLAY PRE_1} results, have you made any changes to your insurance coverage (i.e., health, life, long-term care, disability)?

1 Yes
0 No
2 Choose not to answer
99 Not sure

{PRG: SHOW M1A IF M1=1; OTHERWISE SKIP TO M1B}
{PRG: M1A SELECT ALL THAT APPLY}

M1a. To which insurance coverage did you make changes? *(Please select all that apply.)*

{PRG: SHOW IF H1≠9 OR 10}
1 Health insurance
{PRG: SHOW IF H2≠0 OR 99}
2 Life insurance
{PRG: SHOW IF H4≠0 OR 99}
3 Long-term care insurance
{PRG: SHOW IF H3≠0 OR 99}
4 Disability

{PRG: SHOW IF M1A=1; OTHERWISE SKIP TO FILTER BEFORE M1A_2}
{PRG: M1A_1 SELECT ALL THAT APPLY}

M1a_1. What changes did you make to your health insurance coverage? *(Please select all that apply.)*

1 Stopped coverage
2 Decreased existing coverage
3 Added coverage
4 Increased existing coverage
5 Other *(Please specify)*: [TEXT RESPONSE]

{PRG: SHOW IF M1A=2; OTHERWISE SKIP TO FILTER BEFORE M1A_3}
{PRG: M1A_2 SELECT ALL THAT APPLY}

M1a_2. What changes did you make to your life insurance coverage? *(Please select all that apply.)*

1 Stopped coverage
2 Decreased existing coverage
3 Added coverage
4 Increased existing coverage
5 Other *(Please specify)*: [TEXT RESPONSE]

{PRG: SHOW IF M1A=3; OTHERWISE SKIP TO FILTER BEFORE M1A_4}
{PRG: M1A_3 SELECT ALL THAT APPLY}

M1a_3. What changes did you make to your long-term care insurance coverage? *(Please select all that apply.)*

1 Stopped coverage
2 Decreased existing coverage
3 Added coverage
4 Increased existing coverage
5 Other *(Please specify)*: [TEXT RESPONSE]

{PRG: SHOW IF M1A=4; OTHERWISE SKIP TO FILTER BEFORE M1B}
{PRG: M1A_4 SELECT ALL THAT APPLY}

M1a_4. What changes did you make to your disability insurance coverage? *(Please select all that apply.)*

1 Stopped coverage
2 Decreased existing coverage
3 Added coverage
4 Increased existing coverage
5 Other *(Please specify)*: [TEXT RESPONSE]

M1b. Do you plan to make any {PRG: IF M1=1 DISPLAY “other”} changes to your insurance coverage because of your {DISPLAY PRE_1} results?

1 Yes
0 No
2 Choose not to answer
99 Not sure

{PRG: SHOW IF M1B=1; OTHERWISE SKIP TO M2}

M1b_1. What {PRG: IF M1=1 DISPLAY “other”} changes do you plan to make to your insurance coverage? *(Please select all that apply.)*

1 Stop coverage
2 Decrease existing coverage
3 Add coverage
4 Increase existing coverage
5 Other *(Please specify)*: [TEXT RESPONSE]

M2. Because of your {DISPLAY PRE_1} results, have you made any changes to your financial or retirement plans?

1 Yes
0 No
2 Choose not to answer
99 Not sure

{PRG: SHOW IF M2=1; OTHERWISE SKIP TO FILTER BEFORE M2b}

M2a. What changes have you made to your financial or retirement plans?

[OPEN END RESPONSE]

M2b. Do you plan to make any {PRG: IF M2=1 DISPLAY “other”} changes to your financial or retirement plans because of your {DISPLAY PRE_1} results?

1 Yes
0 No
2 Choose not to answer
99 Not sure

{PRG: SHOW M2B_1 IF M2B =1, OTHERWISE SKIP TO M3}

M2b_1. What {PRG: IF M2=1 DISPLAY “other”} changes do you plan to make to your financial or retirement plans?

[OPEN END RESPONSE]

M3. Because of your {DISPLAY PRE_1} results, have you made any changes related to other advanced planning (e.g., will, advance directives, power of attorney)?

1 Yes
0 No
2 Choose not to answer
99 Not sure

{PRG: SHOW IF M3=1; OTHERWISE SKIP TO FILTER BEFORE M3B}

M3a. What changes have you made related to advanced planning?

[OPEN END RESPONSE]

M3b. Do you plan to make any {PRG: IF M3=1 DISPLAY "other"} changes to your advanced planning because of your {DISPLAY PRE_1} results?

1 Yes
0 No
2 Choose not to answer
99 Not sure

{PRG: SHOW IF M3B=1; OTHERWISE SKIP TO F5}
{PRG: M3B_1 IS EXEMPT FROM SOFT PROMPT}

M3b_1. What {PRG: IF M3=1 DISPLAY "other"} changes do you plan to make to your advanced planning?

[OPEN END RESPONSE]

{Note: Section Header 4: Your Genome Results Report: Attitudes}

Invitae Participant Note {PRG: DO NOT SHOW}
{PRG: SHOW ON ALL PAGES IF PRE_1=9 (INVITAE)}
Invitae Participants: In this survey, ***genome*** refers to ***genetic***, ***genome sequencing*** refers to ***genetic testing***, and ***genome reports*** refer to ***genetic test reports***.

{DESIGN: GRID F5a-F5e}

F5. To what extent do you agree or disagree with the following statements?

1 Strongly disagree
2 Somewhat disagree
3 Neither agree nor disagree
4 Somewhat agree
5 Strongly agree

F5a. The information I received about my genome will influence how I manage my health in the future.
F5b. Having personal genome sequencing made me feel like I have more control over my health.
F5d. Having personal genome sequencing helped me to get a better perspective on my health status.
F5e. What I learned from my personal genome sequencing will help reduce my chances of getting sick.

{DESIGN: GRID F6A-F6P}
{PRG: ONLY SHOW F6D IF PRE_1=1; OTHERWISE DO NOT INCLUDE}
{PRG: ONLY SHOW F6H IF PRE_1=2; OTHERWISE DO NOT INCLUDE}
{PRG: ONLY SHOW F6M & F6Q IF K8=7; OTHERWISE DO NOT INCLUDE}

F6. To what extent do you agree or disagree with the following statements?

1 Strongly disagree
2 Somewhat disagree
3 Neither agree nor disagree
4 Somewhat agree
5 Strongly agree

F6a. I am confident in the quality and accuracy of my {DISPLAY PRE_1} results.
F6i. Through [DISPLAY PRE_1], I learned something to improve my health that I didn't know before.
F6j. I am disappointed that my {DISPLAY PRE_1} results did not tell me more information.
F6k. I found my {DISPLAY PRE_1} results interesting.
F6l. I found the information from {DISPLAY PRE_1} to be fun and entertaining.
F6d. I feel that I got what I paid for through {RESPONSE: PRE_1}.
F6h. I would have utilized in-person genetic counseling services had they been available through {RESPONSE:PRE_1}.
F6c. The educational materials provided by {RESPONSE:PRE_1} were helpful.
F6m. The knowledge I gained about genome sequencing through {RESPONSE:PRE_1} has influenced my medical practice.
F6q. The knowledge I gained about genome sequencing through {RESPONSE:PRE_1} has influenced my clinical research.

{PRG: SHOW IF F6m= 4 OR 5; OTHERWISE SKIP TO B4}

F6m_1. How has the knowledge you gained about genome sequencing through {RESPONSE:PRE_1} influenced your professional practice? (*Please select all that apply.)*

1 It has made me MORE likely to consider genome sequencing for one or more of my patients.
2 It has made me LESS likely to consider genome sequencing for one or more of my patients.
3 It has helped me better understand the benefits of the technology.
4 It has helped me better understand the limitations of the technology.
5 It has helped me better understand the risks of the technology.
6 It has affected my research methods or priorities.
7 It has changed my views on professional guidelines and/or regulations.
8 Other *(Please specify):* [TEXT RESPONSE]

{PRG: SHOW IF F6M_1=6; OTHERWISE SKIP TO FILTER BEFORE F6_M3}

F6_m2. How has the knowledge you gained about genome sequencing through this process affected your research methods or priorities?

[OPEN END RESPONSE]

{PRG: SHOW IF F6M_1=7; OTHERWISE SKIP TO B4}

F6_m3. How has the knowledge you gained about genome sequencing through this process changed your views on professional guidelines and/or regulations?

[OPEN END RESPONSE]

B4. *Please read the following questions and for each one select the response that best applies to you:*

Do you feel the information you have received so far through your personal genome sequencing results with {DISPLAY PRE_1} will…

1 No, definitely not
2 No, probably not
3 Yes, probably
4 Yes, definitely

B4b. …influence what treatment you receive for current or future medical problems?
B4c. …influence decisions you make about your medical care?
B4d. …influence your reproductive decisions?
B4e. …influence what medications you take?
B4f. …influence your end of life planning (e.g., whether you get or change your advanced directive/living will)?

M5. How much do you agree or disagree with the following statements?

1 Strongly disagree
3 Somewhat disagree
4 Neither agree nor disagree
5 Somewhat agree
7 Strongly agree

M5a. I am confident that I understand my {RESPONSE:PRE_1} results.
M5b. It has been easy for me to get information about what my {RESPONSE:PRE_1} results mean.
M5c. I understand what my {RESPONSE:PRE_1} results mean for my health.
M5d. I understand how the DNA changes identified in my {RESPONSE:PRE_1} results may influence my disease risk.
M5e. I understand how my own genetic make-up might affect my disease risk.
M5f. I am able to explain to others what my {RESPONSE:PRE_1} results mean for my health.

{PRG: SHOW IF K3a>0}

INTRO: *The next questions are about some feelings you may have had before or after receiving your personal genome sequencing results.*

PG1. Do you think you may have passed on any genetic diseases or conditions to your children and/or grandchildren?

1 Yes
0 No
2 I don't know

{PRG: SHOW PG9 IF PG1=1, OTHERWISE SKIP TO B5}

PG9. How guilty do you feel about possibly passing on genetic risk to your children or grandchildren?

1 Not at all guilty
2 Not very guilty
3 Somewhat guilty
4 Very guilty
5 Not applicable

{PRG: SHOW PG10 IF PG9=3 OR 4, OTHERWISE SKIP TO B5}

PG10. Was this feeling of guilt prompted by your {RESPONSE:PRE_1} results?

1 Yes
0 No

{PRG: SHOW PG11 IF PG10=1, OTHERWISE SKIP TO B5}

PG11. What about your {RESPONSE:PRE_1} results caused you to feel guilty?

[OPEN END RESPONSE]

{PRG: USE LIKERT SCALE FORMAT FOR B5-B6 AND PLACE ON THE SAME PAGE}

B5. On a scale of 1 to 10, how useful do you think your personal genome sequencing results through {DISPLAY PRE_1} are to you **now**?

1 Not at all useful – 10 Extremely useful

B6. On a scale of 1 to 10, how useful do you think your personal genome sequencing results through {DISPLAY PRE_1} will be to you **in the future**?

1 Not at all useful – 10 Extremely useful

{DESIGN: SHOW GRID F4 AND F4B ON SAME PAGE}
{DESIGN: GRID F4}

F4. In general, how valuable do you feel your personal genome sequencing experience with {DISPLAY PRE_1} was to you?

1 Not at all valuable
2 Not very valuable
3 Somewhat valuable
4 Very valuable

F4b. Please explain why you feel the personal genome sequencing experience with {DISPLAY PRE_1} was or was not valuable.

[OPEN END RESPONSE]

{NOTE: SECTION HEADER 5: Your Entire Genome Sequence}

Invitae Participant Note {PRG: DO NOT SHOW}
{PRG: SHOW ON ALL PAGES IF PRE_1=9 (INVITAE)}
Invitae Participants: In this survey, ***genome*** refers to ***genetic***, ***genome sequencing*** refers to ***genetic testing***, and ***genome reports*** refer to ***genetic test reports***.

INTRO: *In the following section, we are interested in your views on your entire genome sequence, rather than the results report or interpretation. Your "entire genome sequence" is the list of around 3 billion As, Cs, Gs and Ts (or the proportion of this that was successfully sequenced in your case) that make up your genome.*
DS1. In general, how comfortable are you with the idea of sharing your entire genome sequence (not just the report or interpretation)?

1 Very comfortable
2 Somewhat comfortable
3 Somewhat uncomfortable
4 Very uncomfortable

{DESIGN: GRID DS2}

DS2. Would you be willing to share your entire genome sequence…

1 Yes
2 No
3 Not sure

DS2a. …with scientific researchers, as long as your identity remained anonymous?
DS2b. …with scientific researchers, with your identity attached to it?
DS2c. …publicly, as long as your identity remained anonymous?
DS2d. …publicly, with your identity attached to it?

DS3. If someone had access to your entire genome sequence (not just the report or interpretation), and nothing else, how easy or difficult do you think it would be for them to discover your identity?

1 Very easy
2 Quite easy
3 Quite difficult
4 Very difficult
5 Impossible

A10. Through {DISPLAY PRE_1}, do you have access to your entire genome sequence (not just the report or interpretation)?

1 Yes
2 No
3 I don't know

{DESIGN: DISPLAY A11A AND A11B ON SAME SCREEN}

A11a. If it were available, would you be interested in receiving your entire genome sequence (not just the report or interpretation)?

1 Yes
2 No

A11b. Please explain why you would or would not like to receive your entire genome sequence.

[TEXT RESPONSE]

{PRG: SHOW IF A10=1; OTHERWISE SKIP TO F6}

A12. Have you explored your entire genome sequence (not just the report or interpretation) on your own?

1 Yes
2 No

{PRG: SHOW A12A IF A12=2}

A12a. Why have you **NOT** explored your entire genome sequence (not just the report or interpretation) on your own? *(Please select all that apply.)*

1 I have not needed it for anything.
2 I would like to, but have not had the time yet.
3 I am not technically able to do so.
4 I am not interested in doing so on my own.
5 Other *(Please specify):* [TEXT RESPONSE]

{PRG: SHOW DS4 IF A12=1, ELSE GO TO F6}

DS4. Have you conducted or asked others to conduct any additional interpretations of your entire genome sequence?

1 Yes
2 No
3 I do not have access to this type of data
4 I do not know

PRG: SHOW DS4A AND DS4C IF DS4=1, OTHERWISE SKIP TO F6}

DS4a. How much do you trust these additional interpretations?

1 Not at all
2 Not very much
3 Moderately
4 Completely

DS4c. What were these additional interpretations and how will you use them?

[OPEN RESPONSE]

{Note: Section Header 6: General Attitudes Towards Genomics}

Invitae Participant Note {PRG: DO NOT SHOW}
{PRG: SHOW ON ALL PAGES IF PRE_1=9 (INVITAE)}
Invitae Participants: In this survey, ***genome*** refers to ***genetic***, ***genome sequencing*** refers to ***genetic testing***, and ***genome reports*** refer to ***genetic test reports***.

Intro: *In this section, we are interested in learning about your attitudes and thoughts about genome sequencing in general.*

{PRG: GRID F6E – F6P}

F6. Please indicate whether you agree or disagree with each of the following statements.

1 Strongly disagree
2 Somewhat disagree
3 Neither agree nor disagree
4 Somewhat agree
5 Strongly agree

F6e. Health insurance should cover personal genome sequencing.
F6f. Personal genomic information should be part of a standard medical record.
F6g. Parents should be able to get personal genome sequencing for their children if they want to.
F6n. The government should put more effort into regulating personal genome sequencing.
F6o. Personal genome sequencing should only be available to people through their doctor.
F6p. People have a right to access their own personal genomic information without going through a healthcare provider.

M4. Please indicate whether you agree or disagree with each of the following statements.

1 Strongly disagree
2 Somewhat disagree
3 Neither agree nor disagree
4 Somewhat agree
5 Strongly agree

M4a. Once a variant in a gene that affects a person's risk of a disease is found, that disease can always be prevented or cured.
M4b. A healthcare provider can tell a person their exact chance of developing a disease based on the results from genome sequencing.
M4c. Scientists know how all variants of genes will affect a person's chances of developing diseases.
M4d. Even if a person has a variant in a gene that affects their risk of disease, they may not develop that disease.
M4e. Genome sequencing is a routine test that most people can have through their physician's office.
M4f. Genome sequencing may find variants in a person's genes that they can pass on to their children.
M4g. Genome sequencing may give a person information about their chances of developing several different diseases.
M4h. Genome sequencing may find variants in a person's genes that will increase their chance of developing a disease in their lifetime.
M4i. Genome sequencing may find variants in a person's genes that will decrease their chance of developing a disease in their lifetime.
M4j. Genome sequencing may find variants in a person's genes that may determine how they respond to certain medicines.
M4k. A person's health habits, such as diet and exercise, can affect whether or not their genes cause diseases such as heart disease and cancer.

{Section Header 7: A Few More Questions About You}

Invitae Participant Note {PRG: DO NOT SHOW}
{PRG: SHOW ON ALL PAGES IF PRE_1=9 (INVITAE)}
Invitae Participants: In this survey, ***genome*** refers to ***genetic***, ***genome sequencing*** refers to ***genetic testing***, and ***genome reports*** refer to ***genetic test reports***.

INTRO: *In this section, we are interested in learning a bit more about you, your health, and the health of your family. First, we would like to know about any other types of genetic testing you may have had aside from personal genome sequencing through {DISPLAY PRE_1}.*

Q3. Have you ever received personal genetic testing from another source (e.g., 23andMe, carrier screening, BRCA1 or BRCA2 testing)?

1 Yes, one other source
3 Yes, multiple sources
0 No
2 I don't know

{PRG: SHOW Q3b and Q3c IF Q3=1 OR 3; OTHERWISE SKIP TO Q3d}

Q3b. Why did you receive personal genetic testing from another source? (*Please select all that apply.)*

1 Because my doctor recommended it
2 Because my partner or I were pregnant/planning to get pregnant and we wanted to know if there were any conditions or diseases we might pass on to our child
3 Because I was concerned about a condition or disease that runs in my family
4 To diagnose a condition or disease that I already have
5 Because I was curious about health and traits predicted by my genetic make-up
6 Because I was curious about my ancestry
7 To tailor medications to my genetic information
8 Other *(Please specify):* [TEXT RESPONSE]

Q3c. What type(s) of personal genetic testing have you previously received? *(Please select all that apply.)*

1 Carrier screening through a physician (e.g., testing if you are a carrier of cystic fibrosis)
2 Prenatal diagnosis through a physician (e.g., amniocentesis)
3 Testing for a specific gene or genetic condition through a physician (e.g., BRCA1 sequencing)
4 Direct-to-consumer ancestry testing (e.g., Family Tree DNA, 23andMe ancestry report)
5 Direct-to-consumer health testing (e.g., 23andMe health report)
6 Other *(Please specify):* [TEXT RESPONSE]

Q3d. Have you received personal genome sequencing from any source other than {DISPLAY PRE_1}?

1 Yes
2 No

{PRG: SHOW Q3e AND Q3f IF Q3d=1, OTHERWISE SKIP TO K12}
{PRG: DISPLAY Q3e AND Q3f ON SAME PAGE}

Q3e. From what other company or institution did you receive personal genome sequencing?

[OPEN RESPONSE]

Q3f. Please summarize any differences between these services.

[OPEN RESPONSE]

K12. Would you say that in general your health is:

1 Excellent
2 Very good
3 Good
4 Fair
5 Poor

{DISPLAY: J1A & J1B SIDE BY SIDE ON THE SCREEN}
{DISPLAY: J1 & J2 ON SAME PAGE}

J1. What is your height?

J1a. [NUMERIC RANGE 4-7] feet J1b. [NUMERIC RESPONSE, 0-11] inches

J2. What is your weight?

[NUMERIC RANGE 70-400] pounds

C10. In what calendar year did you have your last general physical check-up?

[NUMERIC RANGE 1950-2017]

{DESIGN: GRID C3_1 TO C3_14}

C3. Has a healthcare provider ever told you that you have (or had) any of the following medical conditions?

1 Yes
0 No

C3_1. Arthritis
C3_2. Asthma
C3_3. Cancer
C3_4. Chronic kidney disease
C3_5. Diabetes
C3_6. Eye conditions
C3_7. Gastrointestinal (GI) conditions
C3_8. Heart conditions
C3_9. High cholesterol
C3_10. Lupus
C3_11. Mental illness/psychiatric conditions
C3_12. Neurological conditions (e.g., Alzheimer's disease, Multiple sclerosis, Parkinson's disease)
C3_13. Psoriasis
C3_14. Bleeding disorder

{PRG: SHOW C3_1_1-13 IF ANY C3_1-C3_13=1, OTHERWISE SKIP TO FILTER BEFORE C3_1A}

C3_1_1-13. Do you **currently** have any of the following medical conditions?

1 Yes
0 No

{PRG: SHOW C3_1_1 IF C3_1=1}	C3_1_1.	Arthritis
{PRG: SHOW C3_1_2 IF C3_2=1}	C3_1_2.	Asthma
{PRG: SHOW C3_1_3 IF C3_3=1}	C3_1_3.	Cancer
{PRG: SHOW C3_1_4 IF C3_4=1}	C3_1_4.	Chronic kidney disease
{PRG: SHOW C3_1_5 IF C3_5=1}	C3_1_5.	Diabetes
{PRG: SHOW C3_1_6 IF C3_6=1}	C3_1_6.	Eye conditions
{PRG: SHOW C3_1_7 IF C3_7=1}	C3_1_7.	Gastrointestinal (GI) conditions
{PRG: SHOW C3_1_8 IF C3_8=1}	C3_1_8.	Heart conditions
{PRG: SHOW C3_1_9 IF C3_9=1}	C3_1_9.	High cholesterol
{PRG: SHOW C3_1_10 IF C3_10=1}	C3_1_10.	Lupus
{PRG: SHOW C3_1_11 IF C3_11=1}	C3_1_11.	Mental illness/psychiatric conditions
{PRG: SHOW C3_1_12 IF C3_12=1}	C3_1_12.	Neurological conditions (e.g., Alzheimer's disease, ALS, Multiple sclerosis, Parkinson's disease)
{PRG: SHOW C3_1_13 IF C3_13=1}	C3_1_13.	Psoriasis
{PRG: SHOW C3_1_14 IF C3_14=1}	C3_1_14.	Bleeding disorder

{PRG: SHOW C3_1A IF C3_1=1; OTHERWISE SKIP TO FILTER BEFORE C3_3A}

C3_1a. Which of the following types of **arthritis** has a healthcare provider told you that you have/had? (*Please select all that apply.)*

1 Osteoarthritis ("wear and tear" on joints)
2 Rheumatoid arthritis (joint swelling and stiffness)
3 Other *(Please specify):* [TEXT RESPONSE]

{PRG: SHOW C3_3A IF C3_3=1; OTHERWISE SKIP TO FILTER BEFORE C3_4A}

C3_3a. Which of the following types of **cancer** has a healthcare provider told you that you have/had? (*Please select all that apply.)*

{PRG: SHOW C3_3A.1 IF DEM4B=1 | 3 | 4}
1 Breast cancer
2 Colorectal cancer
3 Esophageal cancer
4 Leukemia
5 Lung cancer
{PRG: SHOW C3_3A.6 IF DEM4B=2 | 3 | 4}
6 Prostate cancer
7 Skin cancer (Melanoma)
8 Stomach cancer
9 Other *(Please specify):* [TEXT RESPONSE]

{PRG: SHOW C3_5A IF C3_5=1; OTHERWISE SKIP TO FILTER BEFORE C3_6A}

C3_5a. Which of the following types of **diabetes** has a healthcare provider told you that you have/had? (*Please select all that apply.)*

1 Type 1 (insulin dependent, juvenile onset)
2 Type 2 (non-insulin dependent)
3 I don't remember

{PRG: SHOW C3_6a IF C3_6=1; OTHERWISE SKIP TO FILTER BEFORE C3_7a}

C3_6a. Which of the following **eye conditions** has a healthcare provider told you that you have/had? (*Please select all that apply.)*

1 Age-related macular degeneration
2 Glaucoma
3 Cataracts
4 Other *(Please specify):* [TEXT RESPONSE]

{PRG: SHOW C3_7A IF C3_7=1; OTHERWISE SKIP TO FILTER BEFORE C3_8A}

C3_7a. Which of the following **gastrointestinal (GI) conditions** has a healthcare provider told you that you have/had? (*Please select all that apply.)*

1 Celiac disease
2 Crohn's disease
3 Ulcerative colitis
4 Other *(Please specify):* [TEXT RESPONSE]

{PRG: SHOW C3_8A IF C3_8=1; OTHERWISE SKIP TO FILTER BEFORE C3_7A}

C3_8a. Which of the following **heart conditions** has a healthcare provider told you that you have/had? (*Please select all that apply.)*

1 Irregular heartbeat (Atrial fibrillation)
2 Coronary artery disease
3 Peripheral arterial disease
4 Blood clotting (Venous thromboembolism)
5 Other *(Please specify):* [TEXT RESPONSE]

{PRG: SHOW C3_11A IF C3_11=1; OTHERWISE SKIP TO FILTER BEFORE C3_12A}

C3_11a. Which of the following **mental illness/psychiatric conditions** has a healthcare provider told you that you have/had? *(Please select all that apply.)*

3 Anxiety disorder
1 Bipolar disorder
2 Depression
4 Other *(Please specify):* [TEXT RESPONSE]

{PRG: SHOW C3_12a IF C3_12=1; OTHERWISE SKIP TO C4}

C3_12a. Which of the following **neurological conditions** has a healthcare provider told you that you have/had? (*Please select all that apply.)*

1 Alzheimer's disease
2 ALS (Lou Gehrig's disease)
3 Multiple sclerosis
4 Parkinson's disease
5 Other *(Please specify):* [TEXT RESPONSE]

{PRG: SHOW C3_14a IF C3_14=1; OTHERWISE SKIP TO C4}

C3_14a. Which of the following bleeding disorders has a healthcare provider told you that you have/had? *(Select all that apply.)*

1 Von Willebrand Disease
2 Hemophilia A (Factor VIII Deficiency)
3 Hemophilia B (Congenital Factor IX Deficiency)
4 Congenital Fibrinogen (Factor I) Deficiency
5 Congenital Prothrombin (Factor II) Deficiency
6 Congenital Factor VII Deficiency
7 Congenital Factor X Deficiency
8 Congenital Factor XIII Deficiency
9 Other (Please specify): [TEXT RESPONSE]

{DESIGN: GRID C4_1-C4_14}

C4. Have any of your blood relatives (a parent, brother or sister, child, grandparent, aunt, uncle, or first cousin) ever had any of the following conditions?

1 Yes
0 No

C4_1 Arthritis
C4_2 Asthma
C4_3 Cancer
C4_4 Chronic kidney disease
C4_5 Diabetes
C4_6 Eye conditions
C4_7 Gastrointestinal (GI) conditions
C4_8 Heart conditions
C4_9 High cholesterol
C4_10 Lupus
C4_11 Mental illness/psychiatric conditions
C4_12 Neurological conditions (e.g., Alzheimer's disease, ALS, Multiple sclerosis, Parkinson's disease)
C4_13 Psoriasis
C4_14 Bleeding disorder

{PRG: SHOW C5 GRID IF ANY OF C4_1 - C4_14 = 1, OTHERWISE SKIP TO E1}
{DESIGN: GRID C5_1-C5_15}
{PRG: SELECT ALL THAT APPLY}

C5. Which of your blood relatives (a parent, brother or sister, child, grandparent, aunt, uncle, or first cousin) have ever had any of the following conditions? (*Please select all that apply.*)

1 A parent
2 A brother or sister
3 A child
4 A grandparent
5 An aunt, uncle, or first cousin

{PRG: SHOW C5_1 IF C4_1 = 1}	C5_1.	Arthritis
{PRG: SHOW C5_2 IF C4_2 = 1}	C5_2.	Asthma
{PRG: SHOW C5_3 IF C4_3 = 1}	C5_3.	Cancer
{PRG: SHOW C5_4 IF C4_4 = 1}	C5_4.	Chronic kidney disease
{PRG: SHOW C5_5 IF C4_5 = 1}	C5_5.	Diabetes
{PRG: SHOW C5_6 IF C4_6 = 1}	C5_6.	Eye conditions
{PRG: SHOW C5_7 IF C4_7 = 1}	C5_7.	Gastrointestinal (GI) conditions
{PRG: SHOW C5_8 IF C4_8 = 1}	C5_8.	Heart conditions
{PRG: SHOW C5_9 IF C4_9 = 1}	C5_9.	High cholesterol
{PRG: SHOW C5_10 IF C4_10 = 1}	C5_10.	Lupus
{PRG: SHOW C5_11 IF C4_11 = 1}	C5_11.	Mental illness/psychiatric conditions
{PRG: SHOW C5_12 IF C4_12 = 1}	C5_12.	Neurological conditions (e.g., Alzheimer's disease, ALS, Multiple sclerosis, Parkinson's disease)
{PRG: SHOW C5_13 IF C4_13 = 1}	C5_13.	Psoriasis
{PRG: SHOW C5_14 IF C4_14 = 1}	C5_14.	Bleeding disorder

{NOTE: DK SHOULD NOT BE MUTUALLY EXCLUSIVE IN FOLLOWING GRIDS}

{PRG: SHOW C5_1a_1 - C5_1a_5 GRID IF C5_1= 1-5, OTHERWISE SKIP TO FILTER BEFORE C5_3a_1- C5_3a_5 GRID}

{DESIGN: GRID C5_1a_1 - C5_1a_5}
{PRG: C5_1a_1 - C5_1a_5 SELECT ALL THAT APPLY}

Please select the type(s) of {PRG: IF PRE_1=1 DISPLAY "**arthritis**"; OTHERWISE DISPLAY "**arthritis**/**immune disorders**"} that each of the following relatives has/had. *(Please select all that apply.)*

1 Osteoarthritis ("wear and tear" on joints)
2 Rheumatoid arthritis (joint swelling and stiffness)
3 Other
99 I don't know

{PRG: SHOW C5_1a_1 IF C5_1=1}	C5_1a_1. A parent
{PRG: SHOW C5_1a_2 IF C5_1=2}	C5_1a_2. A brother or sister
{PRG: SHOW C5_1a_3 IF C5_1=3}	C5_1a_3. A child
{PRG: SHOW C5_1a_4 IF C5_1=4}	C5_1a_4. A grandparent
{PRG: SHOW C5_1a_5 IF C5_1=5}	C5_1a_5. An aunt, uncle, or first cousin

---

{PRG: SHOW C5_3a_1 - C5_3a_5 GRID IF C5_3= 1-5, OTHERWISE SKIP TO FILTER BEFORE C5_5a_1- C5_5a_5 GRID}

{DESIGN: GRID C5_3a_1 - C5_3a_5}
{PRG: C5_3a_1 - C5_3a_5 SELECT ALL THAT APPLY}

Please select the type(s) of **cancer** that each of the following relatives has/had. *(Please select all that apply.)*

1 Breast cancer
2 Colorectal cancer
3 Esophageal cancer
4 Leukemia
5 Lung cancer
6 Prostate cancer
7 Skin cancer (Melanoma)
8 Stomach cancer
9 Other
99 I don't know

{PRG: SHOW C5_3a_1 IF C5_3=1} C5_3a_1. A parent
{PRG: SHOW C5_3a_2 IF C5_3=2} C5_3a_2. A brother or sister
{PRG: SHOW C5_3a_3 IF C5_3=3} C5_3a_3. A child
{PRG: SHOW C5_3a_4 IF C5_3=4} C5_3a_4. A grandparent
{PRG: SHOW C5_3a_5 IF C5_3=5} C5_3a_5. An aunt, uncle, or first cousin

---

{PRG: SHOW C5_5a_1 - C5_5a_5 GRID IF C5_5 = 1-5, OTHERWISE SKIP TO FILTER BEFORE C5_6a_1- C5_6a_5 GRID}

{DESIGN: GRID C5_5a_1 - C5_5a_5}
{PRG: C5_5a_1 - C5_5a_5 SELECT ALL THAT APPLY}

Please select the type(s) of **diabetes** that each of the following relatives has/had. *(Please select all that apply.)*

1 Type 1 (insulin dependent, juvenile onset)
2 Type 2 (non-insulin dependent)
99 I don't know

{PRG: SHOW C5_5a_1 IF C5_5=1} C5_5a_1. A parent
{PRG: SHOW C5_5a_2 IF C5_5=2} C5_5a_2. A brother or sister
{PRG: SHOW C5_5a_3 IF C5_5=3} C5_5a_3. A child
{PRG: SHOW C5_5a_4 IF C5_5=4} C5_5a_4. A grandparent
{PRG: SHOW C5_5a_5 IF C5_5=5} C5_5a_5. An aunt, uncle, or first cousin

---

{PRG: SHOW C5_6a_1 - C5_6a_5 GRID IF C5_6= 1-5, OTHERWISE SKIP TO FILTER BEFORE C5_7a_1- C5_7a_5 GRID}

{DESIGN: GRID C5_6a_1 - C5_6a_5}
{PRG: C5_6a_1 - C5_6a_5 SELECT ALL THAT APPLY}

Please select the type(s) of **eye conditions** that each of the following relatives has/had. *(Please select all that apply.)*

1 Age-related macular degeneration
2 Glaucoma
3 Cataracts
4 Other
99 I don't know

{PRG: SHOW C5_6a_1 IF C5_6=1} C5_6a_1. A parent
{PRG: SHOW C5_6a_2 IF C5_6=2} C5_6a_2. A brother or sister
{PRG: SHOW C5_6a_3 IF C5_6=3} C5_6a_3. A child
{PRG: SHOW C5_6a_4 IF C5_6=4} C5_6a_4. A grandparent
{PRG: SHOW C5_6a_5 IF C5_6=5} C5_6a_5. An aunt, uncle, or first cousin

---

{PRG: SHOW C5_7a_1 - C5_7a_5 GRID IF C5_7 = 1-5, OTHERWISE SKIP TO FILTER BEFORE C5_8a_1- C5_8a_5 GRID}

{DESIGN: GRID C5_7a_1 - C5_7a_5}
{PRG: C5_7a_1 - C5_7a_5 SELECT ALL THAT APPLY}

Please select the type(s) of **gastrointestinal (GI) conditions** that each of the following relatives has/had. *(Please select all that apply.)*

1 Celiac disease
2 Crohn's disease
3 Ulcerative colitis
4 Other
99 I don't know

{PRG: SHOW C5_7a_1 IF C5_7=1} C5_7a_1. A parent
{PRG: SHOW C5_7a_2 IF C5_7=2} C5_7a_2. A brother or sister
{PRG: SHOW C5_7a_3 IF C5_7=3} C5_7a_3. A child
{PRG: SHOW C5_7a_4 IF C5_7=4} C5_7a_4. A grandparent
{PRG: SHOW C5_7a_5 IF C7_4=5} C5_7a_5. An aunt, uncle, or first cousin

---

{PRG: SHOW C5_8a_1 - C5_8a_5 GRID IF C5_8 = 1-5, OTHERWISE SKIP TO FILTER BEFORE C5_11a_1- C5_11a_5 GRID}

{DESIGN: GRID C5_8a_1 - C5_8a_5}
{PRG: C5_8a_1 - C5_8a_5 SELECT ALL THAT APPLY}

Please select the type(s) of **heart conditions** that each of the following relatives has/had. *(Please select all that apply.)*

1 Irregular heartbeat (Atrial fibrillation)
2 Coronary artery disease
3 Peripheral arterial disease
4 Blood clotting (Venous thromboembolism)
5 Other
99 I don't know

{PRG: SHOW C5_8a_1 IF C5_8=1} C5_8a_1. A parent
{PRG: SHOW C5_8a_2 IF C5_8=2} C5_8a_2. A brother or sister
{PRG: SHOW C5_8a_3 IF C5_8=3} C5_8a_3. A child
{PRG: SHOW C5_8a_4 IF C5_8=4} C5_8a_4. A grandparent
{PRG: SHOW C5_8a_5 IF C5_8=5} C5_8a_5. An aunt, uncle, or first cousin

---

{PRG: SHOW C5_11a_1 - C5_11a_5 GRID IF C5_11= 1-5, OTHERWISE SKIP TO FILTER BEFORE C5_12a_1 - C5_12a_5 GRID}

{DESIGN: GRID C5_11a_1 - C5_11a_5}
{PRG: C5_11a_1 - C5_11a_5 SELECT ALL THAT APPLY}

Please select the type(s) of **mental illness/psychiatric conditions** that each of the following relatives has/had. *(Please select all that apply.)*

3 Anxiety disorder
1 Bipolar disorder
2 Depression
4 Other
99 I don't know

{PRG: SHOW C5_11a_1 IF C5_11=1} C5_11a_1. A parent
{PRG: SHOW C5_11a_2 IF C5_11=2} C5_11a_2. A brother or sister
{PRG: SHOW C5_11a_3 IF C5_11=3} C5_11a_3. A child
{PRG: SHOW C5_11a_4 IF C5_11=4} C5_11a_4. A grandparent
{PRG: SHOW C5_11a_5 IF C5_11=5} C5_11a_5. An aunt, uncle, or first cousin

---

{PRG: SHOW C5_12a_1 - C5_12a_5 GRID IF C5_12= 1-5, OTHERWISE SKIP TO E1}

{DESIGN: GRID C5_12a_1 - C5_12a_5}
{PRG: C5_12a_1 - C5_12a_5 SELECT ALL THAT APPLY}

Please select the type(s) of **neurological conditions** that each of the following relatives has/had. *(Please select all that apply.)*

1 Alzheimer's disease
2 ALS (Lou Gehrig's disease)
3 Multiple sclerosis
4 Parkinson's disease
5 Other
99 I don't know

{PRG: SHOW C5_12a_1 IF C5_12=1} C5_12a_1. A parent
{PRG: SHOW C5_12a_2 IF C5_12=2} C5_12a_2. A brother or sister
{PRG: SHOW C5_12a_3 IF C5_12=3} C5_12a_3. A child
{PRG: SHOW C5_12a_4 IF C5_12=4} C5_12a_4. A grandparent
{PRG: SHOW C5_12a_5 IF C5_12=5} C5_12a_5. An aunt, uncle, or first cousin

---

{PRG: SHOW C5_14a_1 - C5_14a_5 GRID IF C5_14= 1-5, OTHERWISE SKIP TO E1}

{DESIGN: GRID C5_14a_1 - C5_14a_5}
{PRG: C5_14a_1 - C5_14a_5 SELECT ALL THAT APPLY}

Please select the type(s) of bleeding disorders that each of the following relatives has/had. *(Please select all that apply.)*

1 Von Willebrand Disease
2 Hemophilia A (Factor VIII Deficiency)
3 Hemophilia B (Congenital Factor IX Deficiency)
4 Congenital Fibrinogen (Factor I) Deficiency
5 Congenital Prothrombin (Factor II) Deficiency
6 Congenital Factor VII Deficiency
7 Congenital Factor X Deficiency
8 Congenital Factor XIII Deficiency
9 Other
99 I don't know

{PRG: SHOW C5_14a_1 IF C5_14=1} C5_14a_1. A parent
{PRG: SHOW C5_14a_2 IF C5_14=2} C5_14a_2. A brother or sister
{PRG: SHOW C5_12a_3 IF C5_14=3} C5_14a_3. A child
{PRG: SHOW C5_14a_4 IF C5_14=4} C5_14a_4. A grandparent
{PRG: SHOW C5_14a_5 IF C5_14=5} C5_14a_5. An aunt, uncle, or first cousin

---

{DESIGN: GRID E1_1-E_12}

E1. Compared to the average person of your age, what would you say your chances are of developing these conditions?

1 Much lower than average
2 Lower than average
3 Average
4 Higher than average
5 Much higher than average
6 I already have this condition

E1_1. Alzheimer's disease
{PRG: SHOW E1_2 IF DEM4b=1 OR 3 OR 4}
E1_2. Breast cancer
{PRG: SHOWE1_3 IF DEM4b=2 OR 3 OR 4}
E1_3. Prostate cancer
E1_4. Colorectal cancer
E1_5. Lung cancer
E1_6. Diabetes
E1_7. Heart disease (Coronary artery disease)
E1_8. Parkinson's disease

{DESIGN: GRID D1a-D1f}

Now we are going to ask you some questions about yourself and how you have been feeling.

D1. *Please read the following statements and for each one select the response that best applies to you.*

Over the **past two weeks**, how often have you:

1 Not at all
2 Several days
3 More than half of the days
4 Nearly every day

D1a. Felt nervous, anxious, or on edge?
D1b. Been unable to stop or control worrying?
D1c. Felt calm and peaceful?
D1d. Been a happy person?
D1e. Had little interest or pleasure in doing things?
D1f. Felt down, depressed, or hopeless?

E2. You will find below a series of statements that describe how people may react to the uncertainties of life. Please use the scale below to describe to what extent each item is characteristic of you.

1 Not at all characteristic of me 1
2 2
3 3
4 4
5 Entirely characteristic of me 5

E2_1. Unforeseen events upset me greatly.
E2_2. It frustrates me not having all the information I need.
E2_3. Uncertainty keeps me from living a full life.
E2_4. One should always look ahead so as to avoid surprises.
E2_5. A small, unforeseen event can spoil everything, even with the best planning.
E2_6. When it’s time to act, uncertainty paralyses me.
E2_7. When I am uncertain, I can’t function very well.
E2_8. I always want to know what the future has in store for me.
E2_9. I can’t stand being taken by surprise.
E2_10. The smallest doubt can stop me from acting.
E2_11. I should be able to organize everything in advance.
E2_12. I must get away from all uncertain situations.

K13. Please select the statement that best describes the role you prefer to take in dealing with your health care:

1 I prefer to make the final decision about which treatment I will receive.
2 I prefer to make the final decision about my treatment after seriously considering my doctor’s opinion.
3 I prefer that my doctor and I share responsibility for deciding which treatment is best for me.
4 I prefer that my doctor make the final decision about which treatment I will receive, but seriously consider my opinion.
5 I prefer to leave all decisions regarding my treatment to my doctor.

{PRG NOTE: SECTION HEADER 8: Wrap-Up}

Invitae Participant Note {PRG: DO NOT SHOW}
{PRG: SHOW ON ALL PAGES IF PRE_1=9 (INVITAE)}
Invitae Participants: In this survey, ***genome*** refers to ***genetic***, ***genome sequencing*** refers to ***genetic testing***, and ***genome reports*** refer to ***genetic test reports***.

You are almost done with the survey! Here is the final set of questions.

S2D. May we contact you about opportunities to participate in future research related to the Personal Genome Sequencing Outcomes Study (e.g., other surveys or interviews)?

1 Yes
0 No

S2E. May we contact you about opportunities to speak with the media about your experience of participating in the Personal Genome Sequencing Outcomes Study?

1 Yes
0 No

{PRG: S2G IS EXEMPT FROM SOFT PROMPT}

S2GA. Is there anything else you would like to share about your experience of participating in {RESPONSE:PRE_0}?

[OPEN ENDED RESPONSE]

S2G. Is there anything else you would like to share about your experience of participating in the Personal Genome Sequencing Outcomes Study thus far?

[OPEN ENDED RESPONSE]

{PRG: SHOW IF PRE_1=2, OTHERWISE SKIP TO DEM6}

DEM2b. What is your PGP ID (huID)?

This identifier is visible in the top right of your account when you're logged in to my.pgp-hms.org. Sharing this will share with our study any current or future genome and health data you publicly share in your PGP profile.

[TEXT RESPONSE validate with regex: ^[hH][uU][0-9A-Fa-f]{6}$]

{PRG: ADD FOLLOWING AS SOFT PROMPT WITH RESPONSE CATEGORIES}

DEM2b_PROMPT. An important aspect of this study is to be able to link your survey results with your personal genome sequencing results. Doing this will give us insight into how people use personal genomic information and how genome sequencing may (or may not) influence their lives. While we can use your survey response without linking it, providing this ID now will allow us to maximize the benefit of this research.

Again, this identifier is visible in the top right of your account when you're logged in to my.pgp-hms.org. You will not be able to make this link with this study at a later time.

Please indicate your wishes below:

1 No thank you, I choose not to provide my PGP ID (huID)
2 My PGP ID (huID) is [TEXT RESPONSE]
3 I allow this study team to obtain my PGP ID (huID) and research report from The Harvard Personal Genome Project (PGP)

{PRG: SHOW IF PRE_1=1; OTHERWISE SKIP TO DEM6}

CONSENT FOR RELEASE OF GENOME REPORTS:

Thank you for contributing to this research study.

We would like to be able to link your responses to your clinical test reports and MyGenome Application provided to you by Illumina, Inc.

By sharing your clinical test report and granting access to your genome through the MyGenome Application, you will help researchers learn more about how individuals understand and think about their genetic results. Sharing this information will give us access to your genome through the MyGenome Application, but is not required to participate in the survey portion of this research.

SHARE1. **Please indicate if you would be willing to share your clinical test report and your access code for the MyGenome Application by selecting ONE of the following two options...**

1 I authorize Illumina, Inc. to provide a copy of my clinical test report to the study team at Brigham and Women's Hospital.
0 I do NOT authorize Illumina, Inc. to provide a copy of my clinical test report to the study team at Brigham and Women's Hospital.

{PRG: SHOW SHARE2 IF SHARE1=1}

SHARE2: Thank you for your willingness to share your clinical report. To verify your permission, please enter your PG number below. Your PG number is located in the header of your clinical test report. *(If your report or your PG Number is not available, please continue to the next question and we will contact you separately for verification.)*

PG0000 [PGNUM: TEXT RESPONSE, REQUIRE 4 CHARACTERS] - [PGSUFFIX: POPLIST, OPTIONS: 1=BLD, 2=DNA]

{PRG: SHOW IF PRE_1=3; OTHERWISE SKIP TO DEM6}

Thank you for contributing to this research study.

We would like to be able to link your responses to your personal genome sequencing results report provided to you by the Mount Sinai study team.

By sharing your personal genome sequencing results report you will help researchers learn more about how individuals understand and think about their personal genomic information. Sharing this information is not required to participate in the survey portion of this research.

HEALTHSEQ_SHARE1. **Please indicate if you would be willing to have your research results report shared by selecting ONE of the following two options...**

1 I authorize Mount Sinai to provide a copy of my personal genome sequencing results report to the study team at Brigham and Women's Hospital.
0 I do NOT authorize Mount Sinai to provide a copy of my personal genome sequencing results report to the study team at Brigham and Women's Hospital.

{PRG: SHOW DEM8 INTRO AND DEM8 ON SAME PAGE}

DEM8 INTRO. We hope to complete a follow-up survey with you in approximately one year. To ensure that we can locate you then, we would like to verify your mailing address, so that we may send you a letter invitation.

The mailing address we currently have on file for you is:

[DISPLAY ADDRESS]

DEM8. Is the above address correct?

1 Yes, that is my current mailing address.
2 No, I would like to edit my mailing address.

{PRG: SUBMIT SURVEY B AS COMPLETE AND PASS TO SURVEY C}

{PRG: SOFT PROMPT ON DEM6 SCREEN – PROMPT IS SET TO TRIGGER IF DEM6A,C,D,F OR BOTH DEM7A & B ARE BLANK}

DEM6. Please provide us with your mailing address:

DEM6a. Street 1: [TEXT RESPONSE]
DEM6b. Street 2: [TEXT RESPONSE]
DEM6c. City: [TEXT RESPONSE]
DEM6d. State/Province: [TEXT RESPONSE]
DEM6f. Zip/Postal Code: [TEXT RESPONSE; PRG: LIMIT TO 10 CHARACTERS]
DEM6e. Country: [TEXT RESPONSE]

DEM7a. Cell Phone: [TEXT RESPONSE]
DEM7b. Home or Office Phone: [TEXT RESPONSE]

DEM6g. Other/International Address Information: [OPEN END RESPONSE]

We have recorded your email as {INSERT DEM2}. Please provide us with an alternative (i.e., home, school, work) that we may use in case we are not able to reach you at your primary email.

DEM7c. Alternative Email: [EMAIL ADDRESS]
